# Revision of the genera *Xanthogaleruca* Laboissière, 1932 and Pyrrhalta Joannis, 1865 (Coleoptera, Chrysomelidae, Galerucinae) of Taiwan, with type designation of *Galerucella lineatipes* Takei

**DOI:** 10.3897/zookeys.1039.64740

**Published:** 2021-05-20

**Authors:** Chi-Feng Lee, Jan Bezděk

**Affiliations:** 1 Applied Zoology Division, Taiwan Agricultural Research Institute, Taichung 413, Taiwan Taiwan Agricultural Research Institute Taichung Taiwan; 2 Mendel University in Brno, Department of Zoology, Fisheries, Hydrobiology and Apiculture, Zemĕdĕlská 1, 613 00, Brno, Czech Republic Mendel University in Brno Brno Czech Republic

**Keywords:** host plant, leaf beetles, new species, new synonym, nomenclature, taxonomy, *
Tricholochmaea
*

## Abstract

The taxonomic status of *Pyrrhalta* Joannis, 1865 and allied genera *Tricholochmaea* Laboissière, 1932 and *Xanthogaleruca* Laboissière, 1934 is discussed based on the study of Taiwanese species. Tentatively, *Xanthogaleruca* and *Pyrrhalta* are regarded as valid genera while *Tricholochmaea* is a synonym of *Pyrrhalta*. Fourteen species are recognized and redescribed, including *P. gressitti* Kimoto, 1969; *P. taiwana* Kimoto, 1969; *P. viridipennis* Kimoto, 1981; *P. igai* Kimoto, 1981; *P. meifena* Kimoto, 1976; *P. maculata* Gressitt & Kimoto, 1963; *P. tsoui* Bezděk & Lee, 2019; *P. semifulva* (Jacoby, 1885); *P. discalis* Gressitt & Kimoto, 1963; *P. ishiharai* Kimoto, 1994; *P. shirozui* Kimoto, 1969; *P. kobayashii* Kimoto, 1974; *P. ohbayashii* Kimoto, 1984; and *P. takizawai* Kimoto, 1996. Taiwanese populations identified as *Xanthogaleruca aenescens* (Fairmaire) were misidentified and those are described as a new species, *X. yuae***sp. nov.***Xanthogaleruca aenescens* is redescribed for comparison. Eight additional new species of *Pyrrhalta* are described: *P. alishanensis***sp. nov.**, *P. houjayi***sp. nov.**, *P. formosanensis***sp. nov.**, *P. jungchani***sp. nov.**, *P. lui***sp. nov.**, *P. meihuai***sp. nov.**, *P. tahsiangi***sp. nov.**, and *P. wulaiensis***sp. nov.** Type specimens of *Galerucella lineatipes* Takei, 1916 were rediscovered and are designated as lectotype and paralectotype. *Galerucella lineatipes* is removed from synonymy with *G. calmariensis* (Linnaeus, 1767) and regarded as a senior synonym of *P. humeralis* (Chen, 1942), **syn. nov.** Most *Pyrrhalta* species can be classified into four species groups based on their morphological and genitalic similarity. host plants and other biological information are provided for almost all species.

## Introduction

The genus *Pyrrhalta* Joannis, 1865 is one of the most speciose genera of Galerucinae. Xue and Yang (2010) recorded 111 species and three subspecies from the Palearctic, Oriental, Australian, and Nearctic regions (cumulated species of *Pyrrhalta*, *Tricholochmaea* and *Xanthogaleruca*). Nie et al. (2017a) treated those three genera separately with 84 species of *Pyrrhalta*, 21 species and two subspecies of *Tricholochmaea*, and nine species of *Xanthogaleruca*. Six new species were described recently by Bezděk and Lee (2019). Two species were transferred from *Pyrrhalta* to *Xanthogaleruca* by Beenen and Talpur (2019).

In Taiwan, Chûjô (1962) recorded no species in his monograph. Kimoto (1969, 1974, 1976, 1981, 1984, 1994, 1996) dealt with almost all Taiwanese species as follows: three new records for *P. aenescens* (Fairmaire), *P. humeralis* (Chen), and *P. maculata* Gressitt & Kimoto, and three new species (*P. gressitti*, *P. shirozui*, and *P. taiwana*) added in 1969; *P. semifulva* Jacoby, *P. discalis* Gressitt & Kimoto, and a new species, *P. kobayashii* added in 1974; *P. aurata* (Maulik) and one new species, *P. meifena* added in 1976; two new species, *P. igai* and *P. viridipennis* added in 1981; one new species, *P. ohbayashii* was described in 1984; *P. aurata* was misidentified and described as a new species, *P. ishiharai* in 1994; and *P. takizawai*, the last new species in 1996 (Table 1). Recently, Bezděk and Lee (2019) described a new species, *P. tsoui*, while dealing with species having maculate elytra. In total, 16 species have been recorded or described from Taiwan previously.

**Table 1.** Taxonomic works on *Pyrrhalta* of Taiwan by Kimoto.

New species	Authority (reference)	New records or nomenclatural acts
*P. gressitti*, *P. shirozui*, *P. taiwana*	Kimoto, 1969	*P. aenescens* (Fairmaire), *P. humeralis* (Chen), *P. maculata* Gressitt & Kimoto
*P. kobayashii*	Kimoto, 1974	*P. semifulva* Jacoby, *P. discalis* Gressitt & Kimoto
*P. meifena*	Kimoto, 1976	*P. aurata* (Maulik)
*P. igai*, *P. viridipennis*	Kimoto, 1981	
*P. ohbayashii*	Kimoto, 1984	
*P. ishiharai*	Kimoto, 1994	*P. aurata* (Maulik): misidentification
*P. takizawai*	Kimoto, 1996	

Taxonomic status of the genera *Pyrrhalta* and its allied genera is controversial. *Tricholochmaea* Laboissière and *Xanthogaleruca* Laboissière are regarded as distinct genera by some European and American taxonomists (e.g., Silfverberg 1974; Riley et al. 2002, 2003; Beenen 2008, 2010); or synonyms with *Pyrrhalta* by Chinese and Japanese taxonomists (e.g., Kimoto and Takizawa 1997; Nie et al. 2012; Yang et al. 2015). Their taxonomic status is tentatively re-evaluated in the present paper by studying the Taiwanese species.

The Taiwan Chrysomelid Research Team (TCRT) was founded in 2005 and is composed of ten members. All of them are amateurs interested in producing a complete inventory of chrysomelid species in Taiwan. Members of the genus *Pyrrhalta* have been collected and studied, and host plants recorded. Life histories for almost all species were documented by laboratory rearing. The results of these efforts are the subject of the current paper.

## Materials and methods

For rearing studies, larvae were placed in small glass containers (diameter 142 mm × height 50 mm) with cuttings from their host plants. When mature larvae began searching for pupation sites, they were transferred to smaller plastic containers (diameter 90 mm × height 57 mm) filled with moist soil (~ 80% of container volume).

For taxonomic study, the abdomens of adults were separated from the forebodies and boiled in 10% KOH solution, followed by washing in distilled water to prepare genitalia for illustrations. The genitalia were then dissected from the abdomens, mounted on slides in glycerin, and studied and drawn using a Leica M165 stereomicroscope. For detailed examinations, a Nikon ECLIPSE 50i microscope was used.

At least three pairs from each species were examined to delimit variability of diagnostic characters. For species collected from more than one locality, at least one pair from each locality was examined. Length was measured from the anterior margin of the eye to the elytral apex, and width at the greatest width of the elytra.

Specimens studied herein are deposited at the following institutes and collections:

**BPBM**Bernice P. Bishop Museum, Hawaii, USA [James Boone];

**CAS**California Academy of Sciences, California, USA [David H. Kavanaugh];

**EUMJ**Ehime University, Matsuyama, Japan [Hiroyuki Yoshitomi];

**IZAS**Institute of Zoology, Chinese Academy of Sciences, Beijing, China [Rui-E Nie];

**JBCB** Jan Bezděk collection, Brno, Czech Republic;

**HSC** Haruki Suenaga collection, Okayama, Japan;

**KMNH**Kitakyushu Museum of Natural History and Human History, Kitakyushu, Japan [Yûsuke Minoshima];

**KUEC**Faculty of Agriculture, Kyushu University, Fukuoka, Japan [Osamu Tadauchi];

**MCZC**Museum of Comparative Zoology, Harvard University, Massachusetts, USA [Philip D. Perkins and Crystal Maier];

**MNHN**Museum National d’Histoire naturelle, Paris, France [Antoine Mantilleri];

**NHMUK**The Natural History Museum, London, UK [Michael F. Geiser, Maxwell V. L. Barclay];

**NMNS**National Museum of Natural Science, Taichung, Taiwan [Jing-Fu Tsai];

**NMPC**National Museum, Prague, Czech Republic [Lukáš Sekerka, Jiří Hájek];

**OMNH**Osaka Museum of Natural History, Osaka, Japan [Shigehiko Shiyake];

**SEHU** Laboratory for Systematic Entomology, Hokkaido University, Sapporo, Japan [Masahiro Ohara]

Exact label data are cited for all type specimens of described species; a double slash (//) divides the data on different labels and a single slash (/) divides the data in different rows. Other comments and remarks are in square brackets: [p] – preceding data are printed, [h] – preceding data are handwritten, [w] – white label, [y] – yellow label, [g] – green label, [b] – blue label, and [r] – red label.

## Taxonomic account

### Xanthogaleruca

Taxon classificationAnimaliaColeopteraChrysomelidae

Laboissière, 1934

1A06DC83-6578-5F6D-BD6F-24742453C2E4

Galerucella (Xanthogaleruca) Laboissière, 1934: 67 (type species: Chrysomela luteola Müller, 1766, by original designation); Ogloblin 1936: 100; Chûjô 1962: 38.
Pyrrhalta (Xanthogaleruca) : Wilcox, 1965: 36.
Xanthogaleruca : Silfverberg, 1974: 7; Riley et al. 2002: 655; Riley et al. 2003: 72; Beenen 2010: 455.

#### Included species.

*Xanthogaleruca aenescens* (Fairmaire, 1878), *X. yuae* sp. nov., and the additional ca. ten Palaearctic species (Beenen 2010, 2019; Nie et al. 2012, 2017a; Beenen and Talpur 2019).

#### Diagnosis.

**Figure 1.**
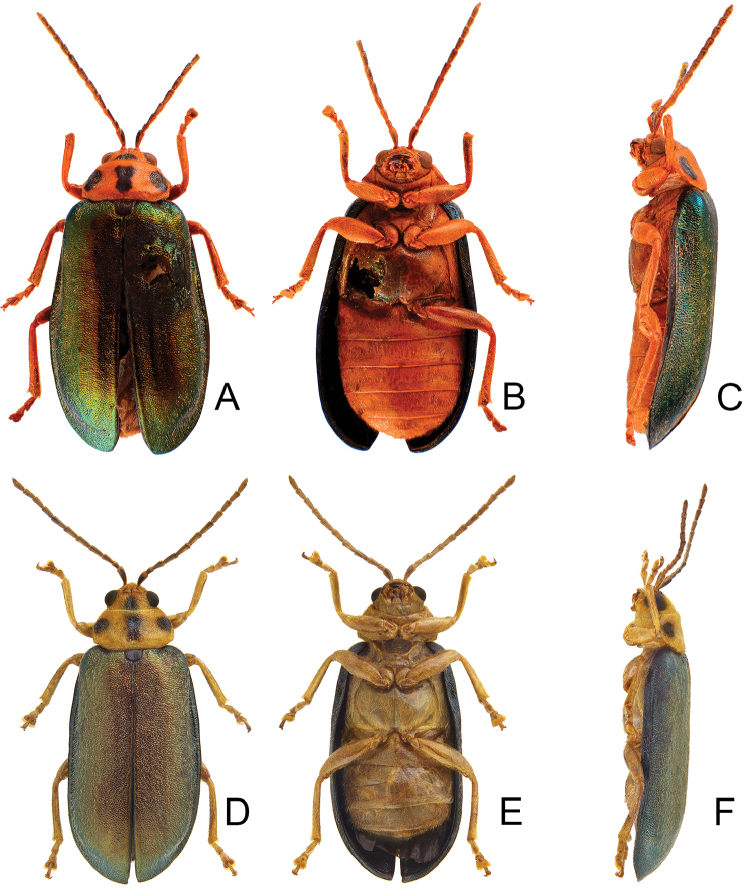
Habitus of *Xanthogaleruca aenescens* (Fairmaire) and *X. yuae* sp. nov. **A***X. aenescens*, female, dorsal view **B** ditto, ventral view **C** ditto, lateral view **D***X. yuae* sp. nov., female, dorsal view **E** ditto, ventral view **F** ditto, lateral view.

**Figure 2.**
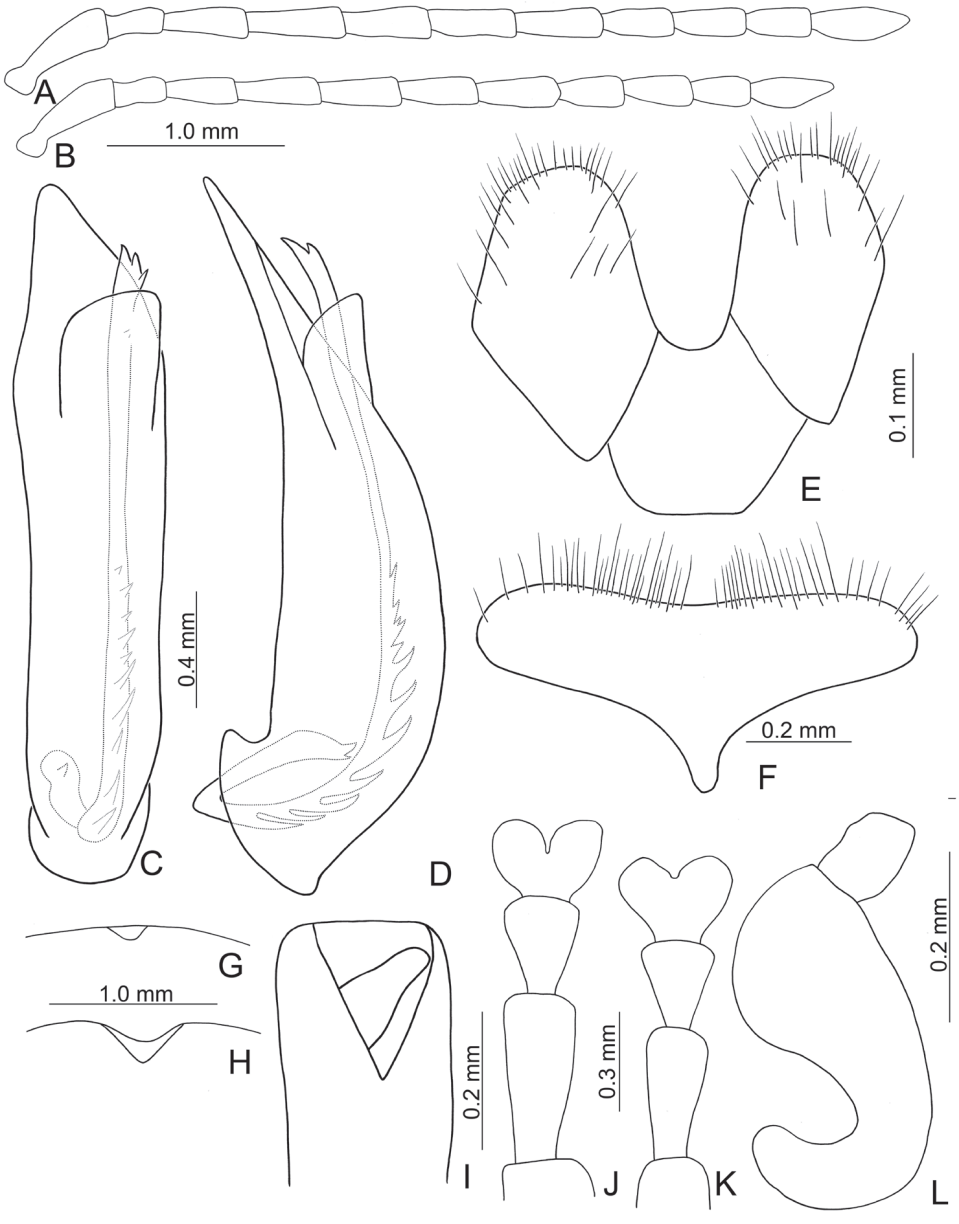
Diagnostic characters of *Xanthogaleruca aenescens* (Fairmaire) **A** antenna, male **B** antenna, female **C** aedeagus, dorsal view **D** ditto, lateral view **E** gonocoxae **F** abdominal ventrite VIII **G** abdominal ventrite V, female **H** abdominal ventrite V, male **I** apex of tibia of middle leg, male **J** tarsi of front leg, male **K** tarsi of front leg, female **L** spermatheca.

**Figure 3.**
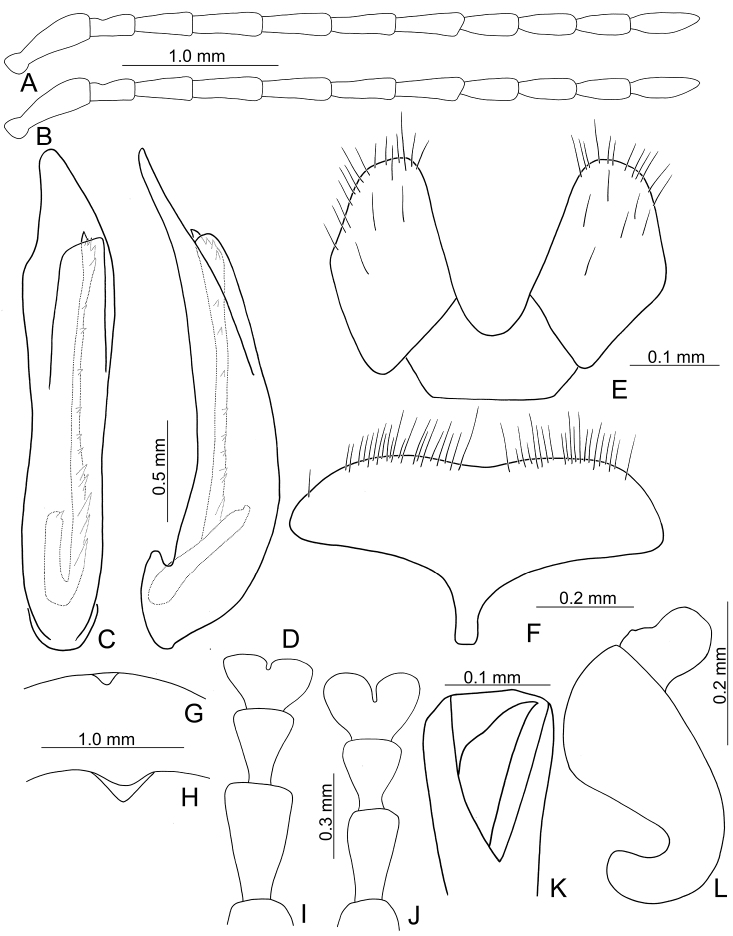
Diagnostic characters of *Xanthogaleruca yuae* sp. nov. **A** antenna, male **B** antenna, female **C** aedeagus, dorsal view **D** ditto, lateral view **E** gonocoxae **F** abdominal ventrite VIII **G** abdominal ventrite V, female **H** abdominal ventrite V, male **I** tarsi of front leg, male **J** tarsi of front leg, female **K** apex of tibia of middle leg, male **L** spermatheca.

Large sized species (7.9–9.5 mm). Antenna slender, antennomeres III–VII long (2.5–3.1 × longer than wide), VIII–X shorter. Body flattened (Fig. 1C, F). Elytra relatively narrower, 1.6–1.8 × longer than wide. Aedeagus (Figs 2C, D, 3C, D) asymmetrical; ostium covered by a more or less sclerotized membrane; endophallic sclerite composed of a single slender sclerite with base recurved, with one row of stout teeth along lateral margin. Ventrite VIII (Figs 2F, 3F) in females well sclerotized, with dense short setae along apical margin; spiculum extremely short. Gonocoxae (Figs 2E, 3E) well sclerotized and with dense short setae along apical margins. Apical margin of abdominal ventrite V with angular depression at middle in males (Figs 2H, 3H), followed by shallow notch; represented by a semicircular depression in females (Figs 2G, 3G). Mesotibia with apical spine in males (Figs 2I, 3K); but mesotarsi with tarsomere I not modified.

#### Biology.

Larvae and adults feed on leaves of *Ulmus* species and *Zelkova serrata* (Thunb.) Makino (Ulmaceae).

#### Remarks.

Tentatively we accept *Xanthogaleruca* as valid genus. Internal sclerite of aedeagus of *Xanthogaleruca* is characteristic, comb-like, and presumed to be an apomorphy (Silfverberg 1974; Beenen 2003, 2008; Matsumura et al. 2017; Beenen and Talpur 2019). Moreover, Nie et al. (2017b) showed phylogenetic distance between *Pyrrhalta* (*P. rufosanguinea* Say, 1827) and *Xanthogaleruca* (*X. maculicollis* (Motschulsky, 1853 and *X. aenescens*). See also Discussion below. In addition, larvae of *X. yuae* sp. nov. pupated on the leaves of the host plant. This differs from the habits of other Taiwanese species of *Pyrrhalta* that pupate in earthen cells.

### Xanthogaleruca aenescens

Taxon classificationAnimaliaColeopteraChrysomelidae

(Fairmaire, 1878)

7D9C2DA1-5700-5A72-9760-636B6979B405

Figs 1A–C , 2

Galeruca aenescens Fairmaire, 1878: 140 (China).
Galerucella aenescens : Fairmaire 1887: 334 (China: Beijing); Weise 1889: 569 (as synonym of Apophylia thalassina (Faldermann, 1835)); Weise 1896: 296 (note); Weise 1924: 54 (catalogue); Laboissière 1926: 58 (distinct species); Bezděk 2003: 98 (excluded from Apophylia).
Galerucella (Xanthogaleruca) aenescens : Laboissière 1934: 67; Ogloblin 1936: 100 (redescription).
Pyrrhalta aenescens : Gressitt & Kimoto, 1963: 443 (China: Jilin, Rehe, Hebei, Shandong, Jiangsu); Medvedev and Voronova 1976: 230 (Mongolia); Medvedev and Zaytsev 1978: 135 (larva); Medvedev and Roginskaya 1988: 115 (host plants); Dubeshko and Medvedev 1989: 153; Li 1992: 185 (NE China); Yang 1992: 555 (China: Hunan); Yang et al. 1997: 864 (China: Hubei); Wang and Yang 2006: 109 (China: Gansu); Xue and Yang 2010: 120 (catalogue); Nie et al. 2012: 133 (biology); Yang et al. 2015 (China: Inner Mongolia, Gansu, Shanxi, Shaanxi, Henan).
Pyrrhalta (Pyrrhalta) aenescens : Wilcox 1971: 84
Pyrrhalta (Xanthogaleruca) aenescens : Medvedev 1982: 101 (key), 261; Medvedev 1992: 579 (key).
Xanthogaleruca aenescens : Lopatin et al. 2004: 129 (catalogue); Beenen 2010: 455 (catalogue); Park et al. 2015: 388 (Korea).

#### Types.

Presumably deposited at the MNHN, but not available for study due to renovation of the roof (Antoine Mantilleri, pers. comm. 2 July 2020); it was studied by Bezděk (2003).

#### Other material.

China. Beijing: 1♂, 1♀ (TARI), Wofosi (臥佛寺), 27.IV.1961, leg. S.-Y. Wang; Hebei: 8♂, 13♀ (TARI), 保定 (= Baoding), 5.IX.1943, leg. A. Tanaka; Tianjin: 1♂, 2♀ (JBCB), Wuquing Co., Dahuanqpu wetland natural conservation, 15.VII.2010, leg. P. Kment; Manchuria (outdated name, refers to Heilongjiang, Jilin, and Liaoning): 5♂ (TARI), 4♀ (TARI), Tokuniji, 23.VII.1937, leg. M. Hanano; 2♂, 2♀ (TARI), Mt. Riutan, Tolisu, 30.V.1937, leg. M. Hanano; 1♂, 3♀ (TARI), same but with “30.VII.1939”; 1♂ (TARI), Anto, 23.VII.1933, leg. K. Nomura.

#### Redescription.

Length 8.2–9.5 mm, width 3.9–4.5 mm. Body color (Fig. 1A–C) yellowish brown; vertex with one rounded black spot at middle, antennae blackish brown but ventral discs of antennomeres IV-VI yellowish brown; pronotum with three large black spots, one spot at center, apically broadened, from basal 1/4 to apical 1/4, two spots laterally; scutellum black; elytra metallic green. Eyes relatively large, interocular space 2.29–2.56 × diameter of eye. Antennae filiform in males (Fig. 2A), length ratios of antennomeres I–XI 1.0: 0.5: 0.7: 0.8: 0.7: 0.7: 0.7: 0.6: 0.6: 0.5: 0.8, length to width ratios of antennomeres I–XI 3.0: 2.2: 2.7: 3.1: 2.7: 2.9: 2.9: 2.4: 2.5: 2.2: 3.0; similar in females (Fig. 2B), length ratios of antennomeres I–XI 1.0: 0.5: 0.6: 0.7: 0.7: 0.7: 0.7: 0.6: 0.6: 0.5: 0.8, length to width ratios of antennomeres I–XI 3.3: 2.0: 2.7: 2.9: 2.8: 2.7: 2.7: 2.1: 2.3: 2.0: 2.9. Pronotum and elytra dorso-ventrally depressed. Pronotum 2.0–2.2 × wider than long, disc with dense coarse punctures and short pubescence, with lateral depressions; lateral margins moderately rounded, apical margin slightly concave, basal margin straight. Elytra elongate, parallel-sided, 1.6–1.7 × longer than wide; disc with dense coarse punctures and short pubescence, with three indistinct longitudinal ridges, of which two near suture, one from humerus. Apical spur of tibia of middle leg short (Fig. 2I); and tarsomere I of front and middle legs not modified in either sex (Fig. 2J, K). Aedeagus (Fig. 2C, D) slender in dorsal view, 5.1 × longer than wide, sides asymmetric, gradually broadened from apex to apical 1/5, parallel from apical 1/5 to near base, apex broadly rounded; strongly curved near base in lateral view, moderately broadened from apex to middle, apex narrowly rounded; ostium covered by a more or less sclerotized membrane; primary endophallic sclerite extremely long, 0.9 × as long as aedeagus, with three apical teeth, and additional longitudinal row of erect teeth from middle to base, become smaller towards apex, one short sclerite connected with base, apex with one short tooth. Gonocoxae (Fig. 2E) transverse, both gonocoxae combined from basal basally connect, with dense short setae along apical margin or areas. Ventrite VIII (Fig. 2F) extremely transverse; disc with dense short setae along apical margin; spiculum extremely short. Receptacle of spermatheca (Fig. 2L) very swollen; pump short and strongly curved; sclerotized proximal spermathecal duct wide and short. Apical margin of abdominal ventrite V with angular depression at middle in males (Fig. 2H), followed by shallow notch; only with semicircle depression in females (Fig. 2G).

#### Host plants.

Ulmaceae: *Ulmus pumila* Linnaeus, *U. laevis* Pallas, and *U. davidiana* Planch (Nie et al. 2012).

#### Remarks.

adults of *X. aenescens* (Fairmaire) and *X. yuae* sp. nov. may be separated from those of other species in the genus by the entirely green elytra, presence of three black spots on the pronotum, elytra with fine and dense punctures. *Xanthogaleruca aenescens* differs from *X. yuae* sp. nov. by the wider aedeagus, 5.1 × longer than wide (Fig. 1C, D) (5.7 × longer than wide (Fig. 3C, D) in *X. yuae* sp. nov.), lacking teeth from near apex to middle of primary endophallic sclerite (with teeth from near apex to middle of primary endophallic sclerite in *X. yuae* sp. nov.), apex of tarsomere I of front legs uniform in both sexes (Fig. 2J, K) (broader in males of *X. yuae* sp. nov. (Fig. 3I, J)), and short apical spur on mesotibia (Fig. 2I) (long apical spur on mesotibia in *X. yuae* sp. nov. (Fig. 2K))

#### Distribution.

Russia (Far East), Mongolia, North China (Gansu, Hebei, Henan, Hunan, Inner Mongolia, Jiangsu, Jilin, Shandong, Shanxi, Shaanxi; Beenen 2010; Yang et al. 2015); Korea (Park et al. 2015).

### Xanthogaleruca yuae sp. nov.

Taxon classificationAnimaliaColeopteraChrysomelidae

D788D1B7-95B8-5112-B429-F76DE59C6532

http://zoobank.org/791BC545-8352-4100-8818-9BDFD162AB08

Figs 1D–F , 3 , 4

Pyrrhalta aenescens : Kimoto, 1969: 28 (Taiwan); Kimoto 1986: 56 (additional records in Taiwan); Kimoto and Chu 1996: 55 (catalogue); Kimoto and Takizawa 1997: 300 (key), 373; Beenen 2010: 455 (catalogue); Yang et al. 2015: 115 (catalogue).

#### Types.

***Holotype*** ♂ (TARI), Taiwan. Taoyuan: Paling (巴陵), 27.V.2009 (reared from eggs), leg. M.-H. Tsou. ***Paratypes***. 3♂, 6♀ (TARI), same data as holotype; 1♀ (TARI), same but with “25.V.2009”; 3♀ (TARI), same but with “26.V.2009”; 7♂, 8♀ (TARI), same but with “28.V.2009”; 37♂, 29♀ (TARI), same but with “29.V.2009”; 1♀ (TARI), same locality, 19.IV.2009, leg. S.-F. Yu; 1♀ (TARI), same locality, 19.VI.2010, leg. H.-J. Chen; Chiayi: 3♀ (TARI), Shounouryo (= Channaoliao, 樟腦寮), near Mt. Ari (阿里山), 14.XII.1937, leg. Y. Yano; 1♂ (TARI), Dokuritsuzan (= Tulishan, 獨立山), near Mt. Ari (阿里山), 14.XII.1937, leg. Y. Yano; Nantou: 2♂ (TARI), Lienhuachi (蓮華池), 23–26.V.1980, leg. K. S. Lin & B. H. Chen; 1♂ (KMNH), Meiyuan (眉原), 21.V.198?, leg. C.-K. Yu (S. Osawa’s Coll.), determined as *P. aenescens* by Kimoto, 1986; Taitung: 1♂ (TARI), Hsinwu (新武), 25.III.2013, leg. C.-L. Lee; 2♀ (TARI), Wulu (霧鹿), 29.III.2011, leg. C.-F. Lee; Taoyuan: 1♀ (TARI), Suleng (四稜), 9.IV.2016, leg. Y.-L. Lin; 1♀ (TARI), Tungyanshan (東眼山), 12.IV.2007, leg. S.-F. Yu.

#### Diagnosis.

Body flattened. Pronotum with three large black spots, one in middle, two laterally. Elytra metallic green

#### Description.

Length 7.9–8.8 mm, width 3.3–3.8 mm. Body color (Fig. 1D–F) yellowish brown; vertex with one rounded black spot at middle, antennae blackish brown but ventral discs of antennomeres IV–VI brown; pronotum with three large black spots, one spot at center, from apical 1/4 to basal 1/4, apically broadened, two spots laterally; scutellum black; elytra metallic green. Eyes relatively large, interocular space 2.33–2.45 × diameter of eye. Antennae filiform in males (Fig. 3A), length ratios of antennomeres I–XI 1.0: 0.5: 0.6: 0.8: 0.8: 0.7: 0.7: 0.6: 0.6: 0.6: 0.7, length to width ratios of antennomeres I–XI 3.1: 2.1: 2.5: 2.9: 3.1: 2.9: 3.0: 2.6: 2.8: 2.7: 3.4; similar in females (Fig. 3B), length ratios of antennomeres I–XI 1.0: 0.4: 0.6: 0.7: 0.7: 0.7: 0.7: 0.6: 0.6: 0.6: 0.8, length to width ratios of antennomeres I–XI 3.4: 1.9: 2.5: 3.0: 2.8: 2.8: 2.8: 2.6: 2.6: 2.5: 3.4. Pronotum and elytra dorso-ventrally depressed. Pronotum 1.9–2.0 × wider than long, disc smooth, with dense coarse punctures and short pubescence, with lateral depressions; lateral margins moderately rounded, apical margin slightly concave, basal margin straight. Elytra elongate, parallel-sided, 1.7–1.8 × longer than wide; disc smooth, with dense, fine punctures and short pubescence, with three indistinct longitudinal ridges, two near suture, one from humerus. Apical spur of tibia of middle leg elongate in males (Fig. 3K); tarsomeres I of front and middle legs apically broadened in males (Fig. 3I), less broadened in females (Fig. 3J). Aedeagus (Fig. 3C, D) slender in dorsal view, 5.8 × longer than wide, sides asymmetric, gradually broadened from apex to apical 1/4, slightly narrowed at middle, apex broadly rounded; moderately curved near base in lateral view, moderately broadened from apex to middle, apex narrowly rounded; ostium covered by a more or less sclerotized membrane; primary endophallic sclerite long, 0.7 × as long as aedeagus, with four apical teeth, and an additional longitudinal row of erect teeth from near apex to base, becoming smaller towards apex, one short sclerite connected with base, apex with one short tooth. Gonocoxae (Fig. 3E) transverse, both gonocoxae combined from basal connection, with a number of short setae along apical margin. Ventrite VIII (Fig. 3F) extremely transverse; disc with dense, short setae along apical margin; spiculum extremely short. Receptacle of spermatheca (Fig. 3L) very swollen; pump short and strongly curved; sclerotized proximal spermathecal duct wide and short. Apical margin of abdominal ventrite V with angular depression at middle in males, followed by shallow notch (Fig. 3H); represented by a semi-circular depression in females (Fig. 3G).

#### Remarks.

Adults of *X. yuae* sp. nov. and *X. aenescens* may be separated from those of other species in the genus by their entirely green elytra, presence of three black spots on the pronotum, and elytra with fine and dense punctures. *Xanthogaleruca yuae* sp. nov. differs from *X. aenescens* in having a narrower aedeagus, 5.7 × longer than wide (Fig. 3C, D) (broader aedeagus (Fig. 2C, D), 5.1 × longer than wide in *X. aenescens*), teeth from near apex to middle of primary endophallic sclerite (lacking teeth from near apex to middle of primary endophallic sclerite in *X. aenescens*), apex of tarsomere I of front legs broader in males than females (Fig. 3I, J) (apex of tarsomere I of front legs uniform in both sexes of *X. aenescens* (Fig. 2J, K)), and long apical spur on mesotibia (Fig. 3K) (short apical spur on mesotibia in *X. aenescens* (Fig. 3I)). This new species was misidentified as *Xanthogaleruca aenescens* by Kimoto (1969, 1986).

#### Host plants.

Larvae and adults feed on leaves of *Zelkova serrata* (Thunb.) Makino (Ulmaceae) (present study).

#### Biology.

**Figure 4.**
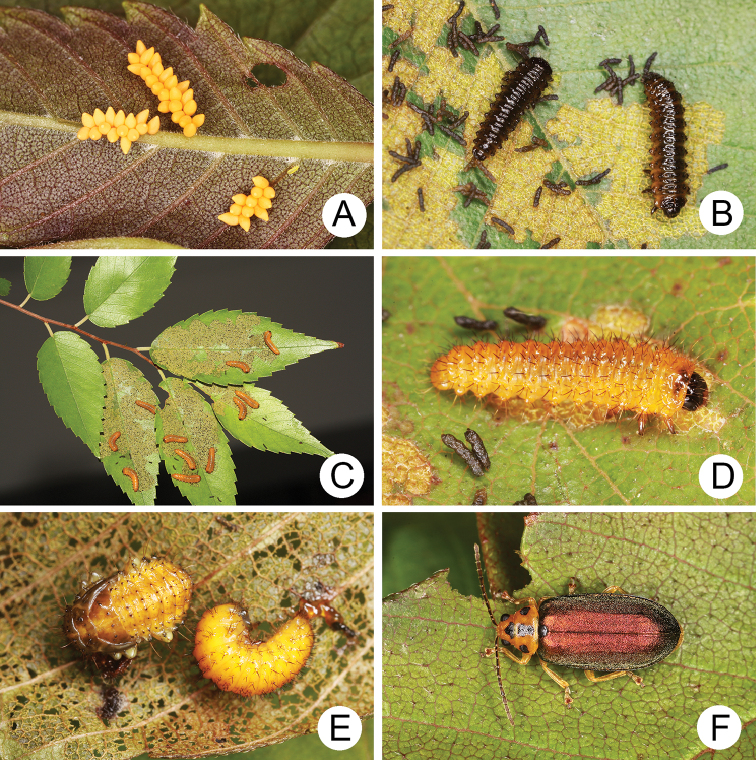
Field photographs of *Xanthogaleruca yuae* sp. nov. on host plant **A** egg masses **B** early instar larva **C** mature larvae **D** single mature larva **E** pupa and prepupa **F** adult.

*Xanthogaleruca yuae* sp. nov. populations are presumed to be univoltine. The following life cycle information is based on our (TCRT) observations made by Mr Mei-Hua Tsou (Lee and Cheng 2010). Females began to deposit an average of 10–20 eggs in two rows of a single egg mass on the undersides of leaves (Fig. 4A) during 22 April 2009. Larvae hatched in 7–8 days. Larvae fed on one side of leaves and left only one layer of tissue at the surface (Fig. 4B, C). The larval duration was 15–21 days. mature larvae (Fig. 4D) expelled an adhesive from the anus, then pupated on the undersides of leaves. Duration of the pupal stage was 8–13 days (Fig. 4E). adults were active during spring (Fig. 4F).

#### Distribution.

Widespread in lowlands of Taiwan.

#### Etymology.

Dedicated to Mrs Su-Fang Yu who was the first member of TCRT to collect specimens of this new species and rear them successfully from eggs to adults.

### Pyrrhalta

Taxon classificationAnimaliaColeopteraChrysomelidae

Joannis, 1865

B4C6B0FA-AA31-5D52-8143-009C74BA2AD3

Pyrrhalta Joannis, 1865: 82 (type species: Galeruca vibruni Paykull, 1799).
Galerucella (Pyrrhalta) : Weise, 1886: 621; Reitter 1913: 138; Ogloblin 1936: 97.
Galeruca (Pyrrhalta) : Seidlitz, 1891: 705.
Decoomanius Laboissière, 1927: 55 (type species: Decoomanius limbatus Laboissière, 1927; by monotypy). Synonymized by Kimoto 1989b: 18.
Chapalia Laboissière, 1929: 269 (type species: Chapalia jeanvoinei Laboissière, 1929; by monotypy). Synonymized by Kimoto 1989b: 18.
Lochmaea (Tricholochmaea) Laboissière, 1932: 963 (type species: Gallerucella semifulva Jacoby, 1885; by original designation). Synonymized by Gressitt and Kimoto 1963: 438.
Tricholochmaea : Chûjô & Kimoto, 1961: 169; Riley et al. 2002: 655; Riley et al. 2003: 71.
Pyrrhalta (Tricholochmaea) : Wilcox, 1965: 37; Wilcox 1971: 80.
Pyrrhalta (Pyrrhalta) : Wilcox, 1971: 84.

#### Remarks.

Weise (1886), Reitter (1913), and Ogloblin (1936) overlooked the fact that *Pyrrhalta* Joannis, 1865 has priority over *Gallerucella* Crotch, 1873. Therefore, the former cannot be a subgenus of the latter (Gressitt and Kimoto 1963). There are no reliable characters for distinguishing *Pyrrhalta* Joannis and *Tricholochmaea* Laboissière. We conclude that establishing species groups as a basis for classification, rather than retaining the generic status of *Tricholochmaea* is a better approach. *Tricholochmaea* is regarded as synonym with *Pyrrhalta* in this study.

### Pyrrhalta gressitti

Taxon classificationAnimaliaColeopteraChrysomelidae

species group

7E117EFB-91AE-567C-8F40-F41BBA93811A

#### Included species.

*Pyrrhalta gressitti* Kimoto, 1969; *P. houjayi* sp. nov.; *P. tahsiangi* sp. nov.; *P. taiwana* Kimoto, 1969; and *P. viridipennis* Kimoto, 1981.

#### Diagnosis.

Small to median sized species (3.5–7.8 mm). Antenna extremely slender, antennomeres III–VI long (3.1–4.5 × longer than wide), VII–X shorter. Body convex. Elytra relatively narrow, 1.6–1.8 × longer than wide. Aedeagus asymmetric, ostium covered by a membrane; endophallic sclerites composed of two slender sclerites (Figs 6C, D; 8C, D; 14D) except single sclerite in *P. tahsiangi* sp. nov. (Fig. 10C, D) and *P. houjayi* sp. nov. (Fig. 12C, D); primary sclerite with several fine teeth near apex (Figs 8C, D; 10C, 14C) except *P. gressitti* Kimoto (Fig. 6C, D) and *P. houjayi* sp. nov. (Fig. 12C, D). Ventrite VIII in female well sclerotized and recurved laterally, apically tapering and with cluster of setae near apex (Figs 6E, 8E, 12E, 14I) except *P. tahsiangi* sp. nov. (Fig. 10E); spiculum long. Gonocoxae apically sclerotized and longitudinally oriented, apex with four long setae (Figs 6G, 8I, 10K, 12F, 14J). Apical margin of abdominal ventrite V moderately concave medially, with deep depression at middle in males (Figs 6I, 8H, 10J, 12I, 14L); concave in females of *P. gressitti* (Fig. 6J), *P. houjayi* sp. nov. (Fig. 8G), and *P. taiwana* (Fig. 12H), or slightly depressed and with one short median internal ridge in females of *P. tahsiangi* sp. nov. (Fig. 10I) and *P. viridipennis* (Fig. 14K). Mesotibia with apical spine in males of *P. gressitti* (Fig. 6F), *P. tahsiangi* sp. nov. (Fig. 10F), and *P. viridipennis* (Fig. 14M) (lacking apical spine in others); mesotarsi with tarsomere I modified only in males of *P. tahsiangi* sp. nov. (Fig. 10H).

#### Biology.

Larvae and adults feed on leaves of *Rhododendron* species or *Vaccinium randaiense* Hayata (Ericaceae).

### Pyrrhalta gressitti

Taxon classificationAnimaliaColeopteraChrysomelidae

Kimoto, 1969

239E643F-89CD-55E9-9A5E-53604D8EF775

**Figure 5.**
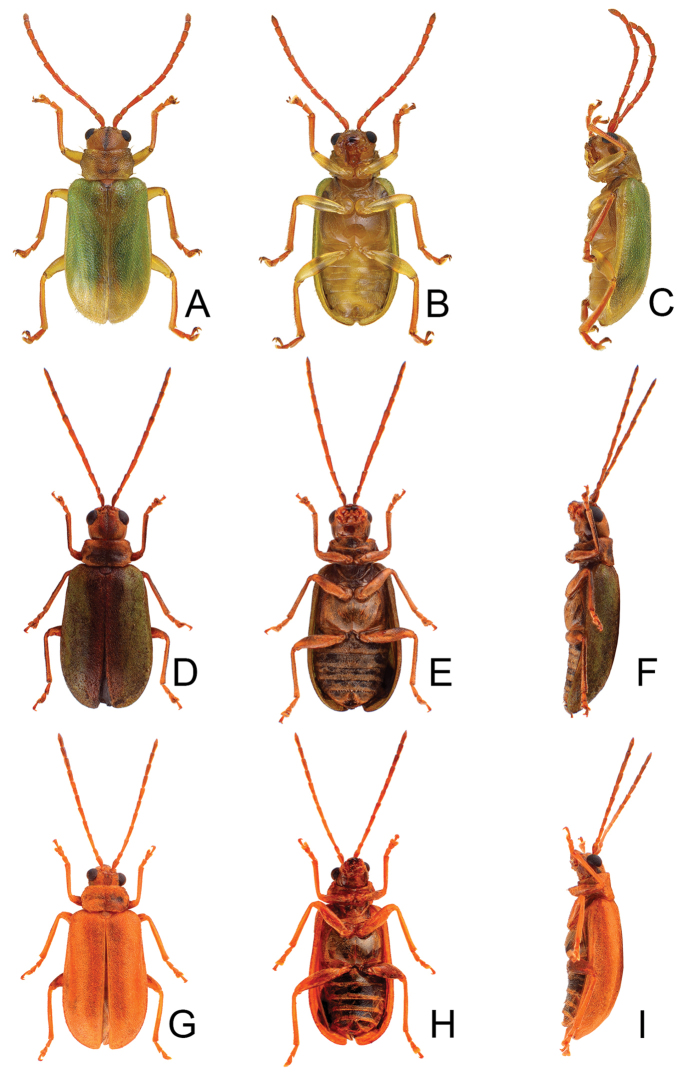
Habitus of *Pyrrhalta gressitti* Kimoto, *P. houjayi* sp. nov., and *P. tahsiangi* sp. nov. **A***P. gressitti*, male, dorsal view **B** ditto, ventral view **C** ditto, lateral view **D***P. houjayi* sp. nov., male, dorsal view **E** ditto, ventral view **F** ditto, lateral view **G***P. tahsiangi* sp. nov., male, dorsal view **H** ditto, ventral view **I** ditto, lateral view.

**Figure 6.**
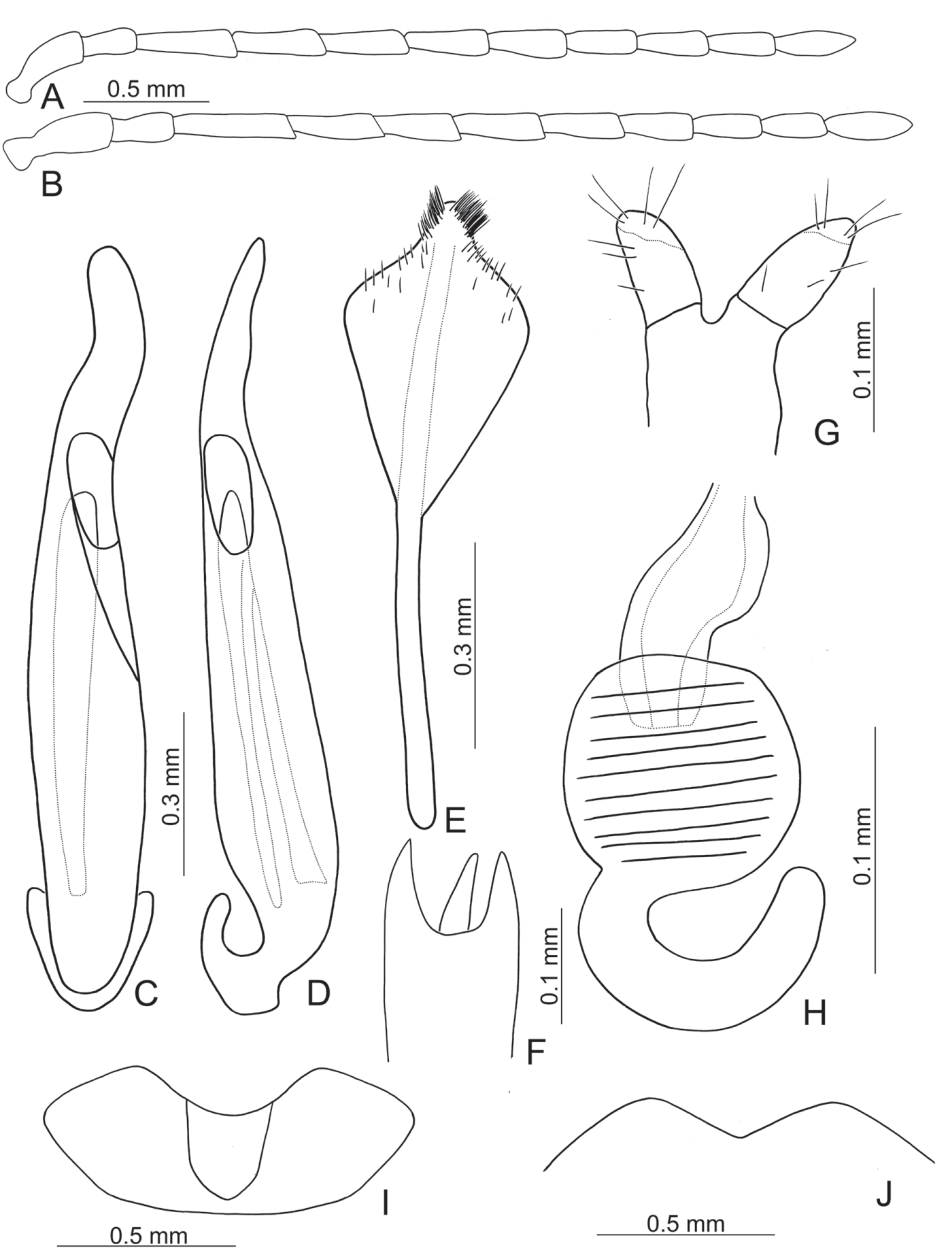
Diagnostic characters of *Pyrrhalta gressitti* Kimoto **A** antenna, male **B** antenna, female **C** aedeagus, dorsal view **D** ditto, lateral view **E** abdominal ventrite VIII **F** apex of tibia of middle leg, male **G**gonocoxae **H** spermatheca **I** abdominal ventrite V, male **J** abdominal ventrite V, female.

**Figure 7.**
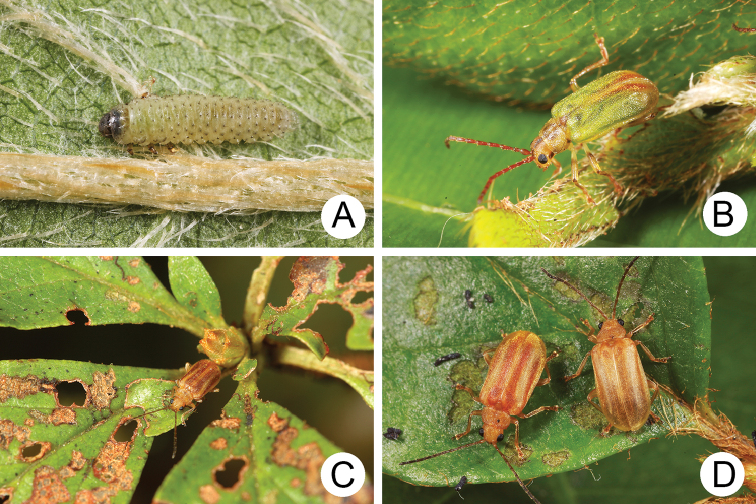
Field photographs of *Pyrrhalta gressitti* Kimoto and *P. tahsiangi* sp. nov. on host plant **A** mature larva of *P. gressitti***B***P. gressitti*, adult **C** adult of *P. tahsiangi* on *Rhododendron chilanshanense***D** adult of *P. tahsiangi* on *R. mariesii*.

Figs 5A–C , 6 , 7A, B

Pyrrhalta gressitti Kimoto, 1969: 25; Kimoto and Chu 1996: 55 (catalogue); Kimoto and Takizawa 1997: 301 (key), 373; Beenen 2010: 452 (catalogue); Xue and Yang 2010: 123 (catalogue); Yang et al. 2015: 116 (catalogue).
Pyrrhalta (Pyrrhalta) gressitti : Wilcox, 1971: 86.

#### Types.

***Holotype*** ♀ (KUEC), labeled: “(TAIWAN) / Alishan / Chiai Hsien / 27. VII. 1966 [p, w] // Pyrrhalta / gressitti / Kimoto, n. sp. [h, w] // HOLOTYPE [p, r]”. ***Paratypes***. 1 ♀ (KMNH) and 1♀ (BPBM): “(TAIWAN) / Alishan / Chiai Hsien / 27. VII. 1966 [p, w] // Pyrrhalta / gressitti / Kimoto, n. sp. [h, w] // PARATOPOTYPE [p, b]”; 1 ex. (KMNH): “(TAIWAN) / Alishan / Chiai Hsien [p] / 30 [h]. VII. 1966 [p, w] // Pyrrhalta / gressitti / Kimoto, n. sp. [h, w] // PARATYPE [p, b]”; 1♂, 1♀ (BPBM): “FORMOSA: / Arisan [阿里山] / VIII-18-1947 / J. L. Gressitt [p, w] // L. Gressitt / Collection [p, w] // Pyrrhalta / gressitti / Kimoto, n. sp. [h, w] // PARATYPE [p, b]”.

#### Other material.

Taiwan. Chiayi: 12♂, 3♀ (TARI), Alishan (阿里山), 5–9.VIII.1981, leg. L. Y. Chou & S. C. Lin; 2♀ (TARI), same locality, 17–20.VIII.1982, leg. K. C. Chou & C. C. Pan; 2♀ (NMNS), same locality, 8.IX.1989, leg. I. S. Hsu; 1♀ (NMNS), same locality, 26.IV.1990, leg. C. C. Chiang; 8♂, 5♀ (TARI), Tatachia (塔塔加), 9.VI.2009, leg. C.-F. Lee; 3♂ (TARI), same locality, 20.VII.2009, leg. H. Lee and S.-F. Yu; Kaohsiung: 1♂ (TARI), Kuanshan Wind Gap (關山啞口), 30.VII.2015, leg. C.-F. Lee; Nantou: 1♂ (NMNS), Patungkuan (八通關), 20–22.VI.1990, leg. J. T. Yang; Pingtung: 2♂, 2♀ (TARI), Peitawushan (北大武山), 24.X.2013, leg. J.-C. Chen; 1♀ (TARI), same but with “12.IX.2015”; Taitung: 15♂, 4♀ (TARI), Hsiangyang (向陽), 2.VII.2009, leg. M.-H. Tsou; 1♀ (TARI), Liyuan (栗園), 19.VI.2013, leg. C.-F. Lee; 1♂, 2♀ (TARI), same locality, 19.VI.2014, leg. J.-C. Chen; 2♀ (TARI), Motien (摩天), 23.V.2011, leg. C.-F. Lee; 2♂ (TARI), same but with “19.VI.2011”.

#### Redescription.

Length 3.9–5.4 mm, width 1.7–2.4 mm. Body color (Fig. 5A–C) yellowish brown; head with median longitudinal black stripe; antennae reddish brown; elytra green but apical 1/3 and lateral margins yellowish brown; outer sides of tibiae more or less darkened. Eyes small, interocular space 2.76–3.48 × diameter of eye. Antennae filiform in males (Fig. 6A), length ratios of antennomeres I–XI 1.0: 0.6: 1.0: 0.9: 0.9: 0.9: 0.8: 0.8: 0.7: 0.7: 0.8, length to width ratios of antennomeres I–XI 3.1: 2.1: 3.5: 3.4: 3.4: 3.3: 2.8: 3.1: 2.8: 2.9: 3.1; similar in females (Fig. 6B), length ratios of antennomeres I–XI 1.0: 0.6: 1.0: 0.8: 0.7: 0.8: 0.7: 0.6: 0.6: 0.6: 0.8, length to width ratios of antennomeres I–XI 3.0: 2.1: 4.4: 3.1: 3.0: 3.1: 3.1: 2.7: 2.7: 2.5: 3.0. Pronotum and elytra convex. Pronotum 1.8–2.0 × wider than long, disc with reticulate microsculpture; with dense, coarse punctures, and short pubescence, with median longitudinal and lateral depressions; lateral margins moderately rounded, widest at apical 1/3, apical margin slightly concave, basal margin straight; anterior and posterior setiferous punctures slightly erect. Elytra elongate and broad, parallel-sided, 1.7 × longer than wide; disc smooth, with dense, coarse punctures, and short pubescence, with one pair of indistinct longitudinal ridges between suture and humeral calli, two indistinct longitudinal ridges arising from humeral calli, inner ridges separated into two at apical 1/3. Apical spur of tibia of middle leg slender (Fig. 6F), and tarsomere I not modified in males. Aedeagus (Fig. 6C, D) slender in dorsal view, 6.3 × longer than wide, asymmetric, curved at apical 1/4, recurved at apical 1/7, broadly rounded, ostium small and located at right side, not covered by membrane; straight but strongly curved near base in lateral view, recurved at apical 1/7, apex narrowly rounded; primary endophallic sclerite elongate, 0.6 × as long as aedeagus, deeply divided in lateral view. Only apices of gonocoxae (Fig. 6G) sclerotized, with several long setae at apical and lateral areas. Ventrite VIII (Fig. 6E) well sclerotized, strongly broadened near apex, outer sides strongly curved, several short setae along apical margin and bearing a cluster of long setae near middle, spiculum long. Receptacle of spermatheca (Fig. 6H) very swollen; pump long and strongly curved; sclerotized proximal spermathecal duct wide and short. Apical margin of abdominal ventrite V moderately concave medially, with deep depression at middle in males (Fig. 6I); only concave in females (Fig. 6J).

#### Remarks.

adults of *P. gressitti* Kimoto and *P. viridipennis* Kimoto are characterized by their partly green elytra, which possess longitudinal ridges. However, *P. gressitti* can be separated from *P. viridipennis* by its smaller body sizes, 3.9–5.4 mm long (5.3–7.8 mm long in *P. viridipennis*), smooth and shining elytra, with coarse punctures (rough elytra with fine punctures in *P. viridipennis*); recurved apex of aedeagus and broadly rounded apex of primary endophallic sclerite lacking teeth (Fig. 6C, D) (curved apex of aedeagus and narrowly rounded apex of primary endophallic sclerite with teeth in *P. viridipennis* (Fig. 14C, D)); slender apical spur of tibia of middle leg in males (Fig. 6F) (small and stout apical spur of tibia of middle leg in males of *P. viridipennis* (Fig. 14M)); and moderately concave apical margin of abdominal ventrite V in females (Fig. 6J) (slightly concave apical margin of abdominal ventrite V with short internal ridge in females of *P. viridipennis* (Fig. 14L)).

#### Host plants.

Larvae and adults feed on leaves of Rhododendron rubropilosum Hayata var. rubropilosum Hayata (Ericaceae) (Fig. 7A, B).

#### Distribution.

The species is widespread at mid-altitudes (1,500–2,500 m) in southern Taiwan.

### Pyrrhalta houjayi sp. nov.

Taxon classificationAnimaliaColeopteraChrysomelidae

20A2092E-3A90-552F-886D-EE8C9035A3D2

http://zoobank.org/5DC94B2C-2EEE-40A1-9EF1-0D9457CCE01D

Figs 5D–F , 8 , 9

#### Types.

***Holotype*** 1♂ (TARI), Taiwan. Pingtung: Lilungshan (里龍山), 30.VI.2016, leg. J.-C. Chen. ***Paratypes***. 2♂♂, 3♀♀ (TARI), same data as holotype; Hsinchu: 1♂ (TARI), Talu trail (大鹿林道), 1.VIII.2015, leg. Y.-L. Lin; Kaohsiung: 1♂, 1♀ (TARI), Chungchihkuan (中之關), 1.VII.2009, leg. S.-F. Yu; 1♂, 2♀♀ (TARI), same locality, 3.VII.2009, leg. M.-H. Tsou; 1♂ (TARI), Shihshan logging trail (石山林道), 19.VIII.2008, leg. C.-T. Yao; 1♀ (NMNS), Tengchih (天池), 6–7.VII.2000, leg. M. L. Chan; Nantou: 1♀ (TARI), Fenghuangshan (鳳凰山), 10.VIII.2011, leg. M.-H. Tsou; 3♂, 5♀ (TARI), Hsitou (溪頭), 28.V.2009, leg. C.-F. Lee; 2♀ (TARI), Juiyanhsi (瑞岩溪), 16.VIII.2015, leg. S.-F. Yu; 1♂ (TARI), Kuantaoshan (關刀山), 5.VII.2013, leg. Y.-L. Lin; 1♂ (TARI), Meifeng (梅峰), 5–9.X.1980, leg. C. C. Chen & C. C. Chien; 4♂, 1♀ (TARI), same locality, 24–26.VI.1981, leg. K. S. & and W. S. Tang; 3♂, 3♀ (TARI), same locality, 15.VII.1982, leg. S. C. Lin & C. N. Lin; 1♂ (TARI), same locality, 4–7.X.1982, leg. K. C. Chou; 2♂, 1♀ (TARI), same but with “1.VI.2009”; 1♂ (TARI), Peitungyanshan (北東眼山), 3.VII.2014, leg. C.-F. Lee; 3♂, 2♀ (NHMUK), Ruei River Major Wildlife Habitat (瑞岩溪野生動物重要棲息環境), 8.VIII.2008, leg. H. Mendel & M. V. L. Barclay; 2♂ (TARI), Tsuifeng (翠峰), 25–27.VI.1981, leg. K. S. Lin & W. S. Tang; 1♀ (TARI), same locality, 1–3.VIII.1981, leg. T. Lin & W. S. Tang; Pingtung: 2♂, 4♀ (TARI), Jinshuiying (浸水營), 16.VI.2011, leg. J.-C. Chen; 1♀ (TARI), Peitawushan (北大武山), 22.IX.2012, leg. J.-C. Chen; 1♀ (TARI), Tahanshan (大漢山), 21.VII.2013, leg. Y.-T. Chung; 1♂ (TARI), same but with “30.VII.2013”; 2♀ (TARI), same but with “29.VI.2018”; 1♀ (TARI), same but with “9.IX.2018”; 1♀ (TARI), same but with “1.IV.2020”; Taichung: 1♀ (TARI), Tahsuehshan (大雪山), 6.IV.2014, leg. C.-S. Lin; Taipei: 1♀ (TARI), Tatungshan (大桶山), 10.VIII.2008, leg. M.-H. Tsao; Taitung: 1♀ (TARI), Liyuan (栗園), 19.VI.2014, leg. J.-C. Chen; 1♂, 1♀ (TARI), Motien (摩天), 5.X.2010, leg. C.-F. Lee; 1♀ (TARI), Wululintao (霧鹿林道), 24.VI.2010, leg. M.-H. Tsou; Taoyuan: 1♂, 3♀ (TARI), Lalashan (拉拉山), 22.VII.2008, leg. H.-J. Chen; 1♂, 4♀ (TARI), same locality, 2.VIII.2008, leg. M.-H. Tsao (= Tsou); 2♂, 1♀ (TARI), same but with “leg. S.-F. Yu”; 1♂, 2♀ (TARI), same locality, 7.VIII.2008, leg. H.-J. Chen; 1♂ (TARI), same locality, 30.VIII.2008, leg. M.-H. Tsao; 1♀ (TARI), same locality, 28.IV.2009, leg. H.-J. Chen; 2♂, 1♀ (TARI), same but with “29.IV.2009”; 1♂, 2♀ (TARI), same locality, 5.V.2009, leg. C.-F. Lee; 1♂ (TARI), same but with “8.V.2009”; 1♂ (TARI), same locality, 21.V.2009, leg. M.-H. Tsou; 1♂, 1♀ (TARI), same locality, 25.VI.2009, leg. S.-F. Yu; 1♂, 3♀ (TARI), same locality, 4.V.2009, leg. C.-F. Lee; 1♂ (TARI), Ssuleng (四稜), 1.VI.2012, leg. S.-F. Yu; 3♂, 4♀ (TARI), Tamanshan (塔曼山), 3.VIII.2008, leg. M.-H. Tsao.

#### Diagnosis.

Elytra smooth, lacking longitudinal ridges; green with wide reddish brown band along suture.

#### Description.

**Figure 8.**
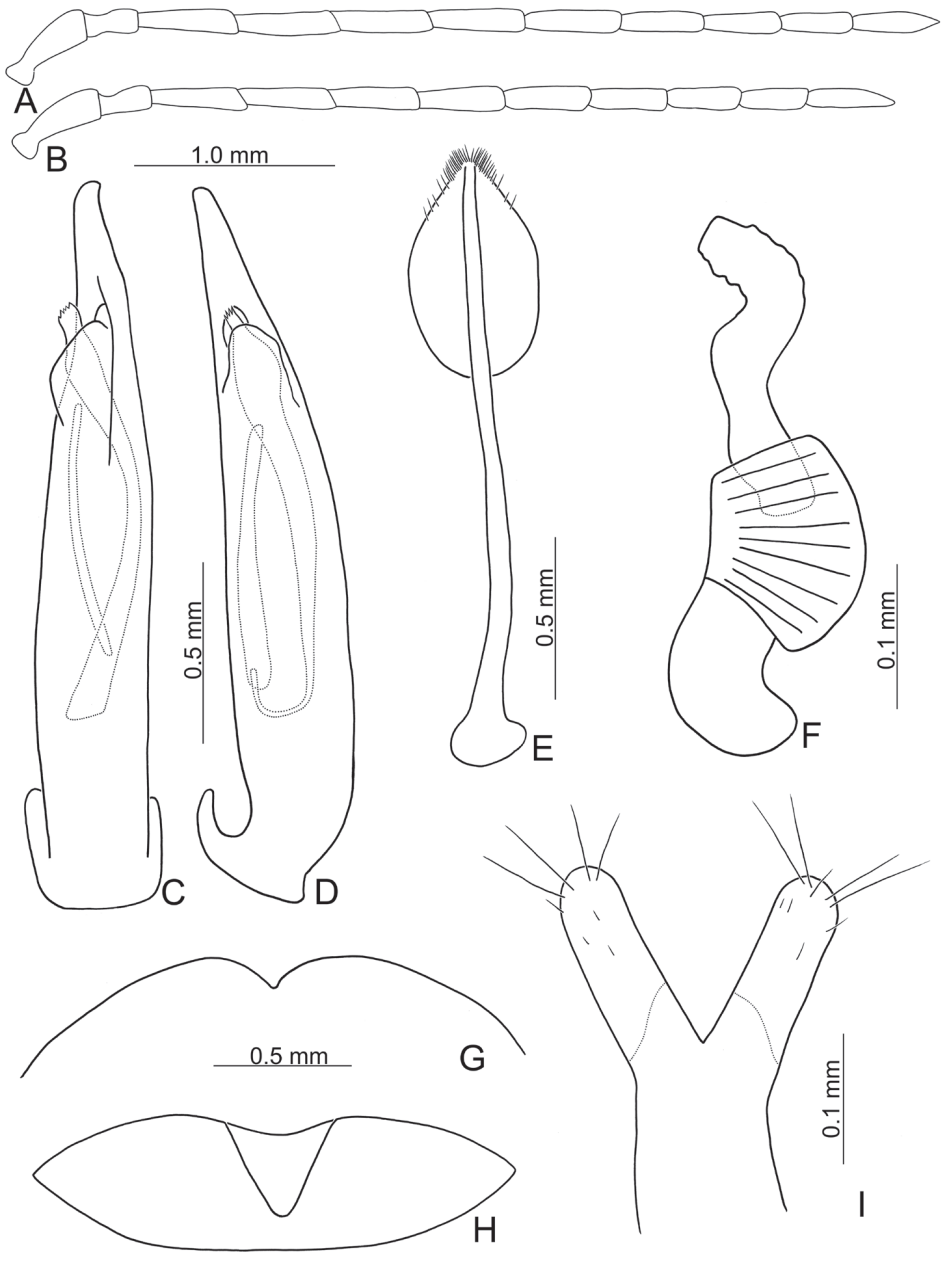
Diagnostic characters of *Pyrrhalta houjayi* sp. nov. **A** antenna, male **B** antenna, female **C** aedeagus, dorsal view **D** ditto, lateral view **E** abdominal ventrite VIII **F** spermatheca **G** abdominal ventrite V, female **H** abdominal ventrite V, male **I** gonocoxae.

Length 4.4–7.5 mm, width 2.5–3.1 mm. Body color (Fig. 5D–F) yellowish brown; head reddish, antenna dark brown; pronotum medially reddish brown; elytra green but with wide reddish brown stripe along suture; scutellum reddish brown; lateral margins of tibiae blackish brown. Eyes small, interocular space 2.20–2.60 × diameter of eye. Antennae filiform in males (Fig. 8A), length ratios of antennomeres I–XI 1.0: 0.6: 1.1: 1.2: 1.2: 1.1: 1.1: 1.0: 0.9: 0.8: 1.0, length to width ratios of antennomeres I–XI 2.7: 2.2: 3.7: 4.1: 4.3: 4.1: 4.2: 3.9: 3.6: 3.3: 4.1; similar in females (Fig. 8B), length ratios of antennomeres I–XI 1.0: 0.5: 1.0: 1.0: 1.0: 0.9: 0.9: 0.8: 0.8: 0.7: 0.9, length to width ratios of antennomeres I–XI 2.9: 2.2: 3.9: 4.2: 4.1: 3.5: 3.7: 3.9: 3.5: 3.3: 4.4. Pronotum and elytra convex. Pronotum 2.0–2.1 × wider than long, disc with reticulate microsculpture; with dense, coarse punctures, and extremely short pubescence, with median longitudinal and lateral depressions; lateral margins rounded, widest at apical 1/3, apical and basal margin slightly concave; anterior and posterior setiferous punctures erect. Elytra elongate and broad, parallel-sided, 1.8 × longer than wide; disc with reticulate microsculpture, with dense, fine punctures, and short pubescence. Apical spur of tibia of middle leg absent and tarsomere I not modified in males. Aedeagus (Fig. 8C, D) slender in dorsal view, 7.4 × longer than wide, asymmetric, curved subapically, apically narrowed from middle, apex narrowly rounded; ostium large, covered by a membrane; straight but strongly curved near base in lateral view, slightly curved at middle, apex narrowly rounded; two endophallic sclerites elongate, primary sclerite 0.7 × as long as aedeagus, apex with several fine teeth, basally recurved; secondary sclerite small, 0.5 × as long as the longer sclerite. Only apices of gonocoxae (Fig. 8I) sclerotized, elongate, with several short setae near apex, and four long setae at near apex. Ventrite VIII (Fig. 8E) well sclerotized, strongly broadened near apex, outer sides strongly curved, several short setae along apical margin and bearing cluster of long setae medially, spiculum long. Receptacle of spermatheca (Fig. 8F) slightly swollen; pump short and strongly curved; sclerotized proximal spermathecal duct wide and long. Apical margin of abdominal ventrite V slightly concave medially, with deep depression but with indistinct margin at middle in males (Fig. 8H); bearing median notch in females (Fig. 8G).

#### Variation.

Specimens from southern Taiwan possess a broader aedeagus and the broader endophallic sclerite near apex that is almost straight in lateral view.

#### Remarks.

**Figure 9.**
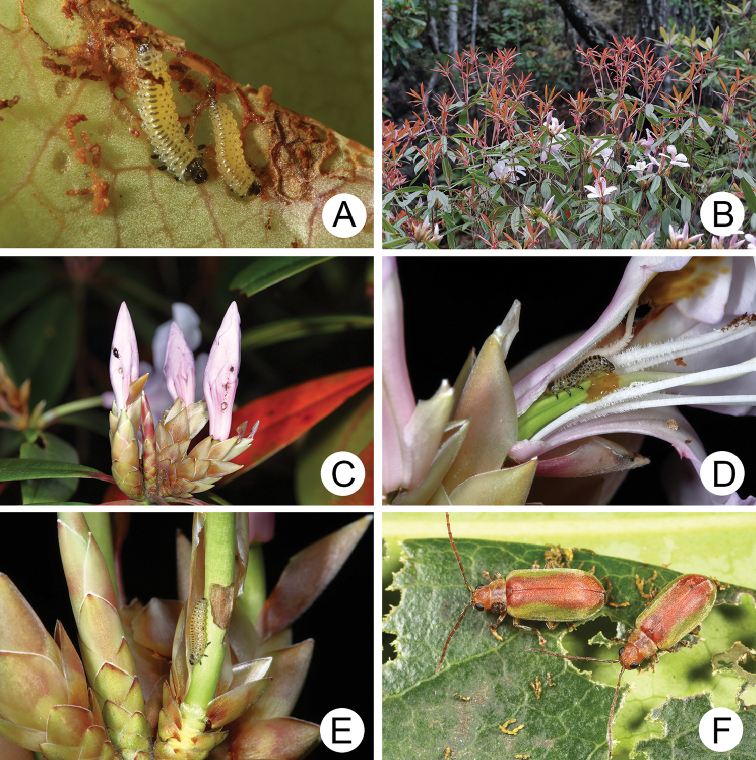
Field photographs of *Pyrrhalta houjayi* sp. nov. on host plant **A** young larvae feeding on tender shoots **B** host plant blooming and sprouting at the same time in Tahsuehshan (大雪山) **C** flower buds with holes caused by larvae **D** Larva found inside the flower buds **E** one larva feeding on pedicels **F** adults.

Adults of *P. houjayi* sp. nov. and *P. taiwana* Kimoto are characterized by their partly green elytra lacking longitudinal ridges. *Pyrrhalta houjayi* sp. nov. can be distinguished from *P. taiwana* by presence of the wide brown band along the suture of the elytra, and more slender elytra (Figs 5D, 9F), 1.8 × longer than wide (lacking brown band on elytra, and wider elytra (Figs 11A, 13D), 1.6 × longer than wide in *P. taiwana*); slender aedeagus, 7.4 × longer than wide, with apex curved to right (Fig. 8C) (broad aedeagus, 6.8 × longer than wide, with tapering apex (Fig. 12C) in *P. taiwana*); two endophallic slerites, primary endophallic sclerite with teeth at apex (Fig. 8C, D) (one endophallic sclerite slender, lacking teeth at apex (Fig. 12C, D) in *P. taiwana*).

#### Host plants.

Larvae and adults feed on leaves of *Rhododendron leptosanthum* Hayata (Ericaceae)

#### Biology.

The first author, Mrs Hsueh Lee, and Mr Hou-Jay Chen found young larvae feeding on tender shoots (Fig. 9A) April 1, 2009 in Lalashan, northern Taiwan. mature larvae started to burrow into soil and built underground chambers for pupation April 11. The newly eclosed adults emerged from soil April 28. The first author and Mr. Ta-Hsiang Lee found the host plants blooming and sprouting at the same time (Fig. 9B) April 21, 2010 in Tahsuehshan, central Taiwan. Many larvae were found inside flower buds with holes (Fig. 9C, D). Some larvae preferred to feed on pedicels (Fig. 9E). Newly emerged adults appeared during late spring, into summer (Fig. 9F).

#### Distribution.

This new species is widespread at mid-altitudes (1,500–2,500 m) in Taiwan.

#### Etymology.

Dedicated to Mrs Su-Fang Yu who was the first member of TCRT to collect specimens of this new species and rear them successfully from eggs to adults.

### Pyrrhalta tahsiangi sp. nov.

Taxon classificationAnimaliaColeopteraChrysomelidae

259339CC-148F-5163-9E7E-837018BEC04C

http://zoobank.org/6856834F-F395-492B-95C7-30D7F948A495

**Figure 10.**
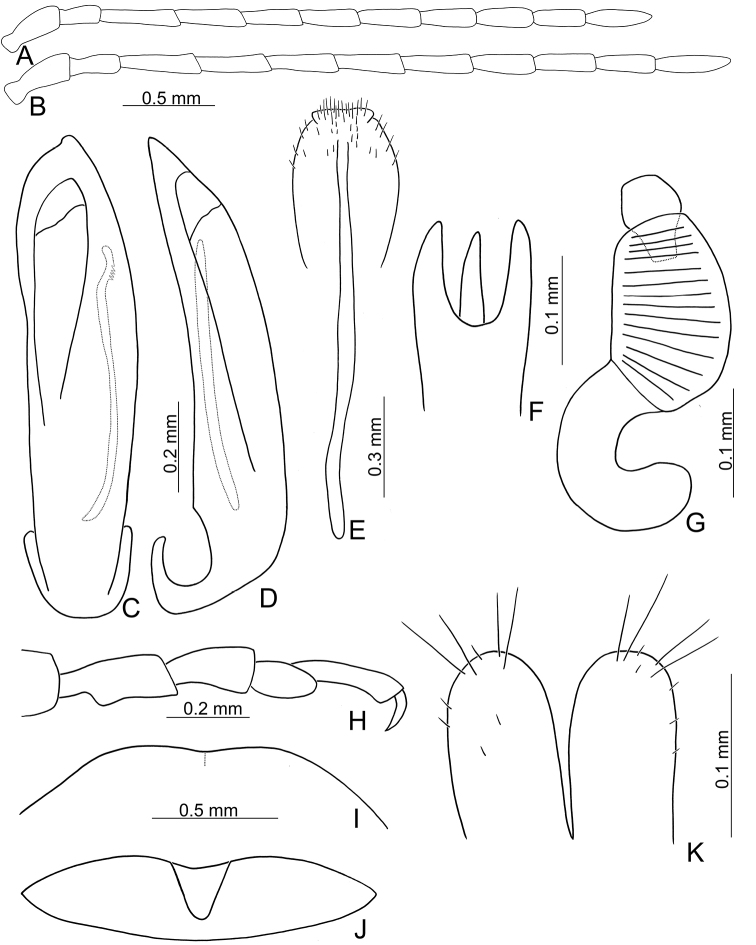
Diagnostic characters of *Pyrrhalta tahsiangi* sp. nov. **A** antenna, male **B** antenna, female **C** aedeagus, dorsal view **D** ditto, lateral view **E** abdominal ventrite VIII **F** apex of tibia of middle leg, male **G** spermatheca **H** tarsi of middle leg, male **I** abdominal ventrite V, female **J** abdominal ventrite V, male **K** gonocoxae.

Figs 5G–I , 7C, D , 10

#### Types.

***Holotype*** ♂ (TARI), Taiwan. Ilan: Tsuifenghu (翠峰湖), 4.VII.2010, leg. M.-H. Tsou. ***Paratypes***. 3♂, 8♀ (TARI), same data as holotype; Ilan: 6♀ (TARI), Yuanyanghu (鴛鴦湖), 23.VIII.2011, leg. M.-H. Tsou; 1♂, 5♀ (TARI), same but with “leg. H. Lee”; 7♀ (TARI), Taipingshan (太平山), 25.V.2009 (reared from larvae), leg. C.-F. Lee.

#### Diagnosis.

Elytra smooth, lacking longitudinal ridges; yellowish brown, with brown longitudinal stripes.

#### Description.

Length 4.8–5.6 mm, width 2.1–2.4 mm. Body color (Fig. 5G–I) yellowish brown; antennae brown, four apical antennomeres darkened; elytra with two pairs of wide, poorly defined, longitudinal brown stripes, one pair near suture, the other arising from humeral calli. Eyes small, interocular space 2.55–2.58 × diameter of eye. Antennae filiform in males (Fig. 10A), length ratios of antennomeres I–XI 1.0: 0.6: 1.2: 1.0: 0.9: 0.9: 1.0: 0.9: 0.9: 0.8: 1.0, length to width ratios of antennomeres I–XI 2.6: 2.2: 4.3: 3.8: 3.8: 3.3: 3.4: 3.3: 3.0: 3.0: 4.0; similar in females (Fig. 10B), length ratios of antennomeres I–XI 1.0: 0.7: 1.1: 1.0: 1.0: 1.0: 1.0: 0.9: 0.9: 0.8: 1.1, length to width ratios of antennomeres I–XI 2.6: 2.6: 4.0: 3.6: 3.5: 3.4: 3.7: 3.2: 3.4: 3.3: 4.3. Pronotum and elytra convex. Pronotum 1.8–1.9 × wider than long, disc smooth; with extremely dense, coarse punctures, and short pubescence, with median longitudinal and lateral depressions; lateral margins slightly rounded, widest at apical 1/3, apical and basal margin slightly concave; anterior and posterior setiferous punctures slightly erect. Elytra elongate and broad, parallel-sided, 1.7 × longer than wide; disc rugose, with dense, coarse punctures, and short pubescence. Apical spur of tibia of middle leg slender (Fig. 10F), and tarsomere I ax-shaped in lateral view, with narrowed basal half and expanded apical half (Fig. 10H) in males. Aedeagus (Fig. 10C, D) broad in dorsal view, 5.0 × longer than wide, parallel-sided, asymmetric, curved at middle, apex narrowly rounded; ostium large, mostly covered by membrane; straight but strongly curved near base in lateral view, apex narrowly rounded; primary endophallic sclerite elongate, 0.5 × as long as aedeagus, with several fine teeth near apex. Only apices of gonocoxae (Fig. 10K) sclerotized, short, with several short setae near apex, and four long setae near apex. Ventrite VIII (Fig. 10E) well sclerotized, apex truncate, plate-shaped and projecting, several extremely short setae along lateral margin and apical area, apical margin with cluster of long setae near middle, spiculum extremely long. Receptacle of spermatheca (Fig. 10G) swollen; pump short and strongly curved; sclerotized proximal spermathecal duct wide and extremely short. Apical margin of abdominal ventrite V slightly concave medially and with deep depression in males (Fig. 10J); while slightly concave and with short internal ridge at middle in females (Fig. 10I).

#### Remarks.

Adults of this new species are easily separated from other members of the species group by their yellowish brown elytra bearing longitudinal brown stripes and modified tarsi of the middle leg. In addition, some putative autapomorphies are found in genitalic characters, including the characteristic shape of the aedeagus and single endophallic sclerite bearing teeth near the apex (Fig. 10C). The truncate apex of abdominal ventrite VIII in females is also diagnostic (Fig. 10E) (tapering apex of abdominal ventrite VIII in females of others).

#### Host plants.

adults feed on leaves of *Rhododendron chilanshanense* Kurashige (Fig. 7C); larvae and adults feed on leaves of *R. mariesii* Hemsl. and E. H. Wilson (Ericaceae) (Fig. 7D).

#### Biology.

The first author and Mr Ta-Hsiang Lee collected young larvae on tender leaves of *Rhododendron mariesii* May 1, 2009 in Taipingshan, northeastern Taiwan, and reared them in the laboratory. Newly eclosed adults emerged from soil May 25. Mr. Mei-Hua Tsou collected a number of adults July 5, 2010 at the same locality (= Tsuifenghu) (Fig. 7C). The first author, Mrs. Hsueh Li, and Mr. Mei-Hua Tsou found adults feeding on leaves of *R. chilanshanense* (Fig. 7D) August 23, 2011 in Yuanyanghu, northeastern Taiwan.

#### Distribution.

This new species is restricted to mid-altitudes (1,000–2,000 m) in northeastern Taiwan.

#### Etymology.

Dedicated to Mr. Ta-Hsiang Lee. He and the first author were the first to find larvae of this new species and rear them successfully to adults.

### Pyrrhalta taiwana

Taxon classificationAnimaliaColeopteraChrysomelidae

Kimoto, 1969

7E8D5C90-C18B-51D3-8002-5DF5DFAA6102

**Figure 11.**
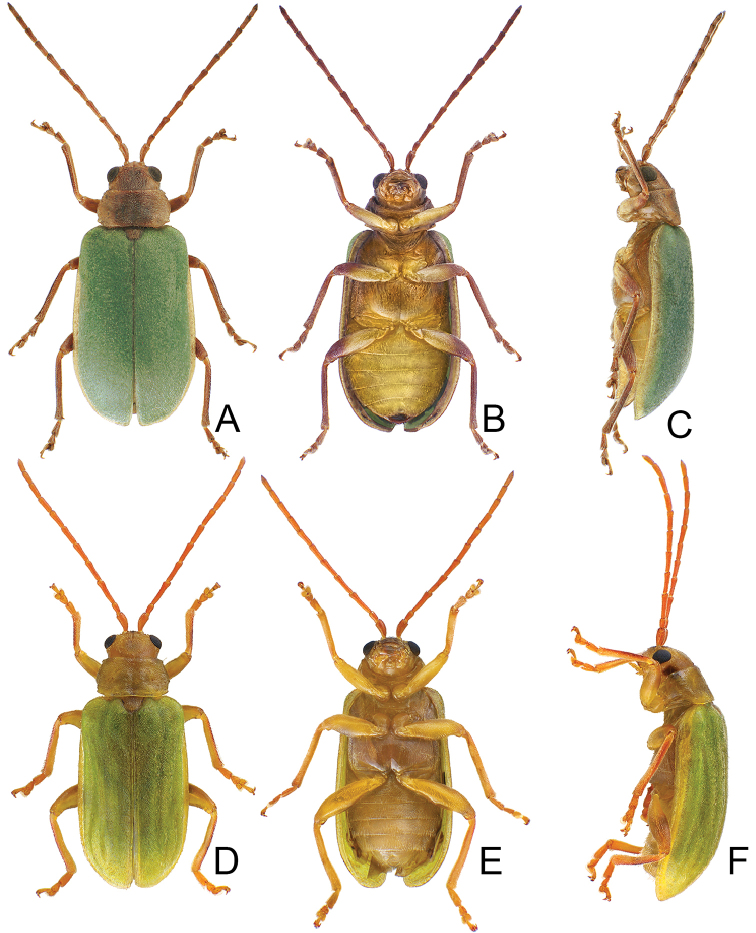
Habitus of *Pyrrhalta taiwana* Kimoto and *P. viridipennis* Kimoto **A***P. taiwana*, male, dorsal view **B** ditto, ventral view **C** ditto, lateral view **D***P. viridipennis*, male, dorsal view **E** ditto, ventral view **F** ditto, lateral view.

**Figure 12.**
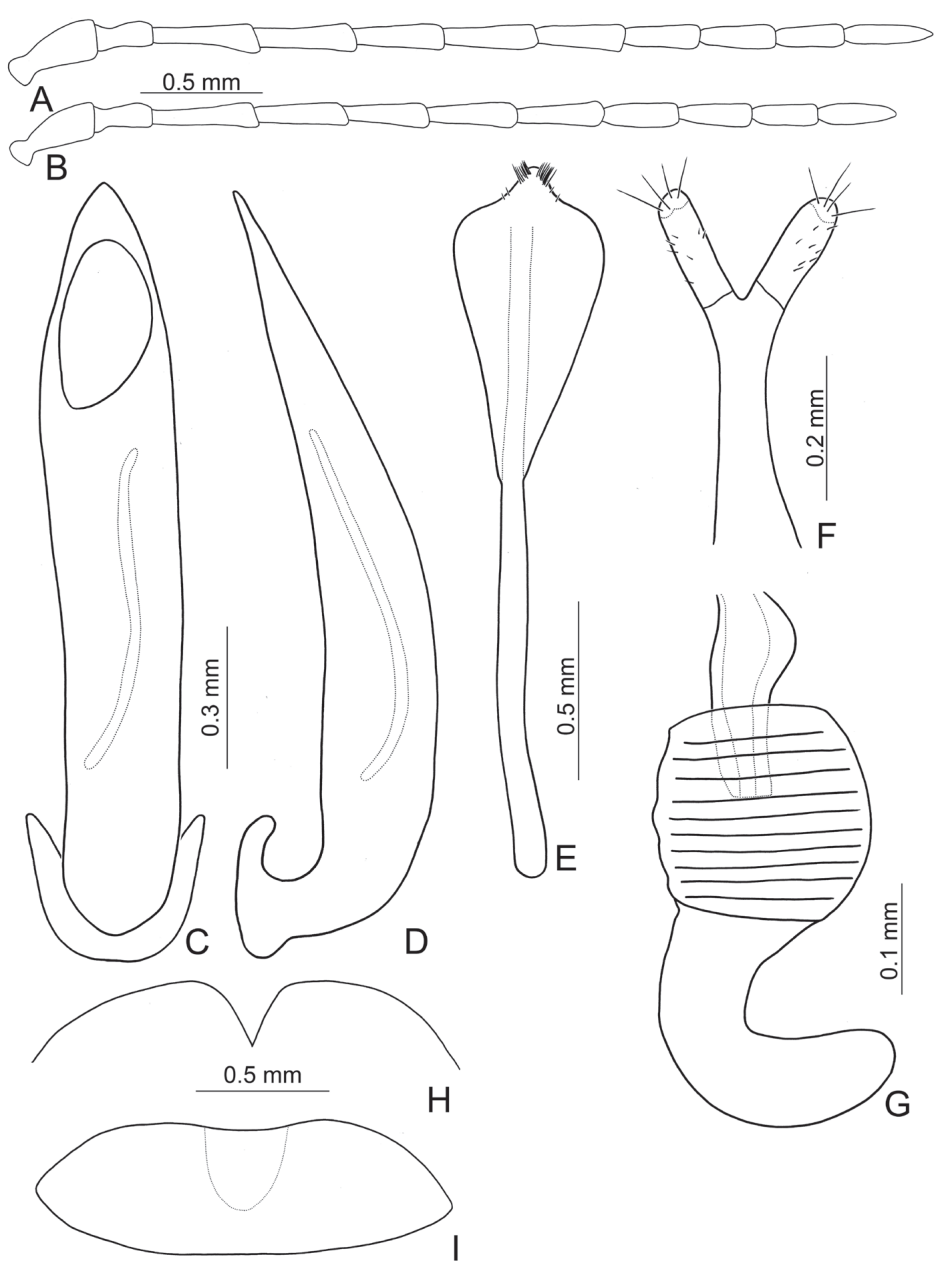
Diagnostic characters of *Pyrrhalta houjayi* sp. nov. **A** antenna, male **B** antenna, female **C** aedeagus, dorsal view **D** ditto, lateral view **E** abdominal ventrite VIII **F** spermatheca **G** gonocoxae **H** abdominal ventrite V, female **I** abdominal ventrite V, male.

**Figure 13.**
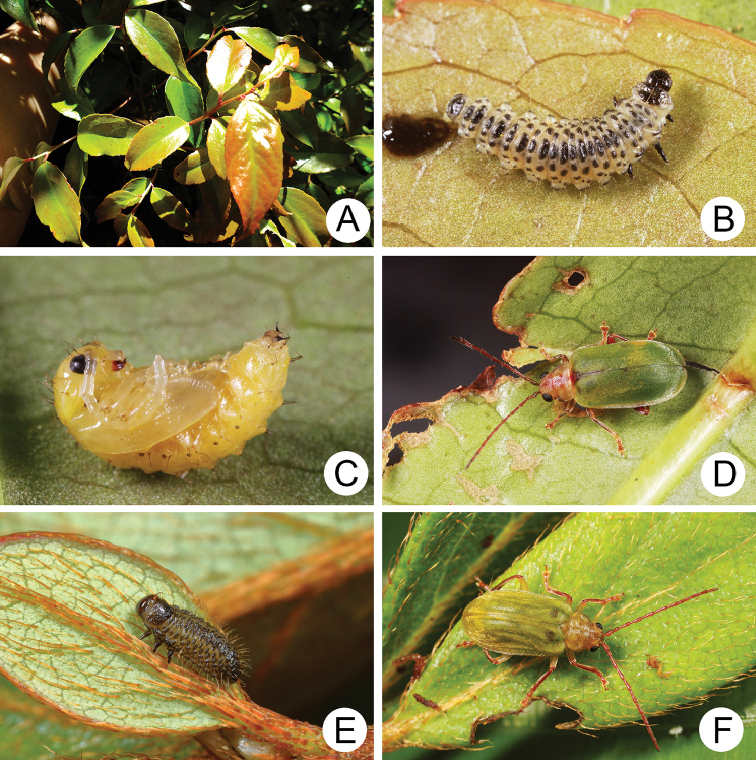
Field photographs of *Pyrrhalta taiwana* Kimoto and *P. viridipennis* Kimoto on host plant **A** host plant for *P. tahsiangi*, *Vaccinium randaiense***B** mature larva of *P. taiwana***C** pupa of *P. taiwana***D** adult of *P. taiwana***E** Third-instar larva of *P. viridipennis***F** adult of *P. viridipennis*.

Figs 11A–C , 12 , 13A–D

Pyrrhalta taiwana Kimoto, 1969: 27 (Taiwan); Kimoto and Chu 1996: 57 (catalogue); Kimoto and Takizawa 1997: 300 (key), 374; Beenen 2010: 453 (catalogue); Xue and Yang 2010: 130 (catalogue); Yang et al. 2015: 121 (catalogue).
Pyrrhalta (Pyrrhalta) taiwana : Wilcox, 1971: 90 (catalogue).

#### Types.

***Holotype*** ♂ (KUEC): “[Formosa] / Hassenzan [= Pahsienshan, 八仙山] (Taichû-shû) / Kahodai [= Chiaobaotai, 佳保台]-Reimei [= Liming, 黎明] / 12. Vii. 1932 / Teiso Esaki [p, w] // Pyrrhalta/ taiwana / Kimoto, n. sp. [h, w] // HOLOTYPE [p, r]”.

#### Other material.

Taiwan. Chiayi: 11♂, 7♀ (TARI), Tzuchung (自忠), 5.VI.2011 (reared from larvae), leg. C.-F. Lee; Kaohsiung: 2♂, 2♀ (TARI), Chungchihkuan (中之關), 3.VII.2009, leg. S.-F. Yu; 1♂, 2♀ (TARI), same locality, 1.VII.2019, leg. M.-H. Tsou; 3♂ (TARI), Tengchih (藤枝), 31.VII.2008, leg. C.-T. Yao; Taichung: 1♂ (TARI), Tahsuehshan (大雪山), 23.VII.2011, leg. J.-C. Chen; Taitung: 6♂, 7♀ (TARI), Lichia trail (利嘉林道), 15.VII.2014, leg. B.-X. Guo.

#### Redescription.

Length 5.6–7.0 mm, width 2.6–3.0 mm. Body color (Fig. 11A–C) yellowish brown; elytra green but with wide yellow stripe along lateral margin; lateral margins of tibiae darkened. Eyes small, interocular space 2.33–2.58 × diameter of eye. Antennae filiform in males (Fig. 12A), length ratios of antennomeres I–XI 1.0: 0.6: 1.1: 1.0: 1.0: 1.0: 0.9: 0.8: 0.8: 0.7: 1.0, length to width ratios of antennomeres I–XI 2.5: 2.3: 3.7: 3.6: 3.6: 3.6: 3.4: 3.2: 3.1: 3.1: 4.9; similar in females (Fig. 12B), length ratios of antennomeres I–XI 1.0: 0.6: 1.1: 1.0: 0.9: 0.8: 0.9: 0.8: 0.8: 0.7: 0.8, length to width ratios of antennomeres I–XI 2.5: 2.3: 4.0: 3.7: 3.5: 3.6: 3.5: 3.3: 3.1: 3.0: 3.7. Pronotum and elytra convex. Pronotum 2.1–2.2 × wider than long, disc with reticulate microsculpture; with dense, coarse punctures, and short pubescence, with median longitudinal and lateral depressions; lateral margins rounded, widest at middle, apical and basal margin slightly concave; anterior and posterior setiferous punctures strongly erect. Elytra elongate and broad, parallel-sided, 1.6 × longer than wide; disc smooth, with dense, fine punctures, and short pubescence. Apical spur of tibia of middle leg absent and tarsomere I not modified in males. Aedeagus (Fig. 12C, D) slender in dorsal view, 6.6 × longer than wide, parallel-sided, asymmetric, apically narrowed from apical 1/5, apex acute; ostium large, not covered by a membrane; straight but strongly curved near base in lateral view, slightly curved at middle, apex narrowly rounded; primary endophallic sclerite elongate, 0.4 × as long as aedeagus. Only apices of gonocoxae (Fig. 12F) sclerotized, elongate, with several short setae near apex, and four long setae in apical area. Ventrite VIII (Fig. 12E) well sclerotized, strongly broadened near apex, outer sides strongly curved, several short setae along apical margin and bearing cluster of long setae medially, spiculum long. Receptacle of spermatheca (Fig. 12G) very swollen; pump short and strongly curved; sclerotized proximal spermathecal duct wide and short. Apical margin of abdominal ventrite V slightly concave medially, with deep depression with an indistinct margin medially in males (Fig. 12I); deep notch in females (Fig. 12H).

#### Remarks.

Adults of *P. taiwana* Kimoto and *P. houjayi* sp. nov. are characterized by their partly green elytra without longitudinal ridges. *Pyrrhalta taiwana* can be distinguished from *P. houjayi* sp. nov. by the entirely green elytra, except lateral margins, and wider elytra (Figs 11A, 13D), 1.6 × longer than wide (presence of the wide brown band along suture of elytra, and more slender elytra (Figs 5D, 9F), 1.8 × longer than wide in *P. houjayi* sp. nov.); broad aedeagus, 6.8 × longer than wide, with tapering apex (Fig. 12C) (slender aedeagus, 7.4 × longer than wide, with apex curved to right (Fig. 8C) in *P. houjayi* sp. nov.); one endophallic sclerite, slender, lacking teeth at apex (Fig. 12C, D) (two endophallic slerites, primary endophallic sclerite with teeth at apex (Fig. 8C, D) in *P. houjayi* sp. nov.).

#### Host plants.

Larvae and adults feed on leaves of *Vaccinium randaiense* Hayata (Fig. 13A); adults feed on leaves of *Rhododendron leptosanthum* Hayata (Ericaceae).

#### Biology.

Mrs Su-Fang Yu found adults feeding on leaves of *Rhododendron leptosanthum* July 3, 2009 in Chungchihkung, southern Taiwan. The first author and Mr. Mei-Hua Tsou found a number of larvae feeding tender leaves of *Vaccinium randaiense* May 9, 2011 in Tzuchung, southern Taiwan. These were reared in the laboratory. They began burrowing into soil May 12 and built underground chambers for pupation. The newly eclosed adults emerged from soil May 25.

#### Distribution.

This species is widespread at mid-altitudes (1,500–2,500 m) in southern Taiwan.

### Pyrrhalta viridipennis

Taxon classificationAnimaliaColeopteraChrysomelidae

Kimoto, 1981

663C33A4-F197-5A4D-9C63-02A9275263FD

**Figure 14.**
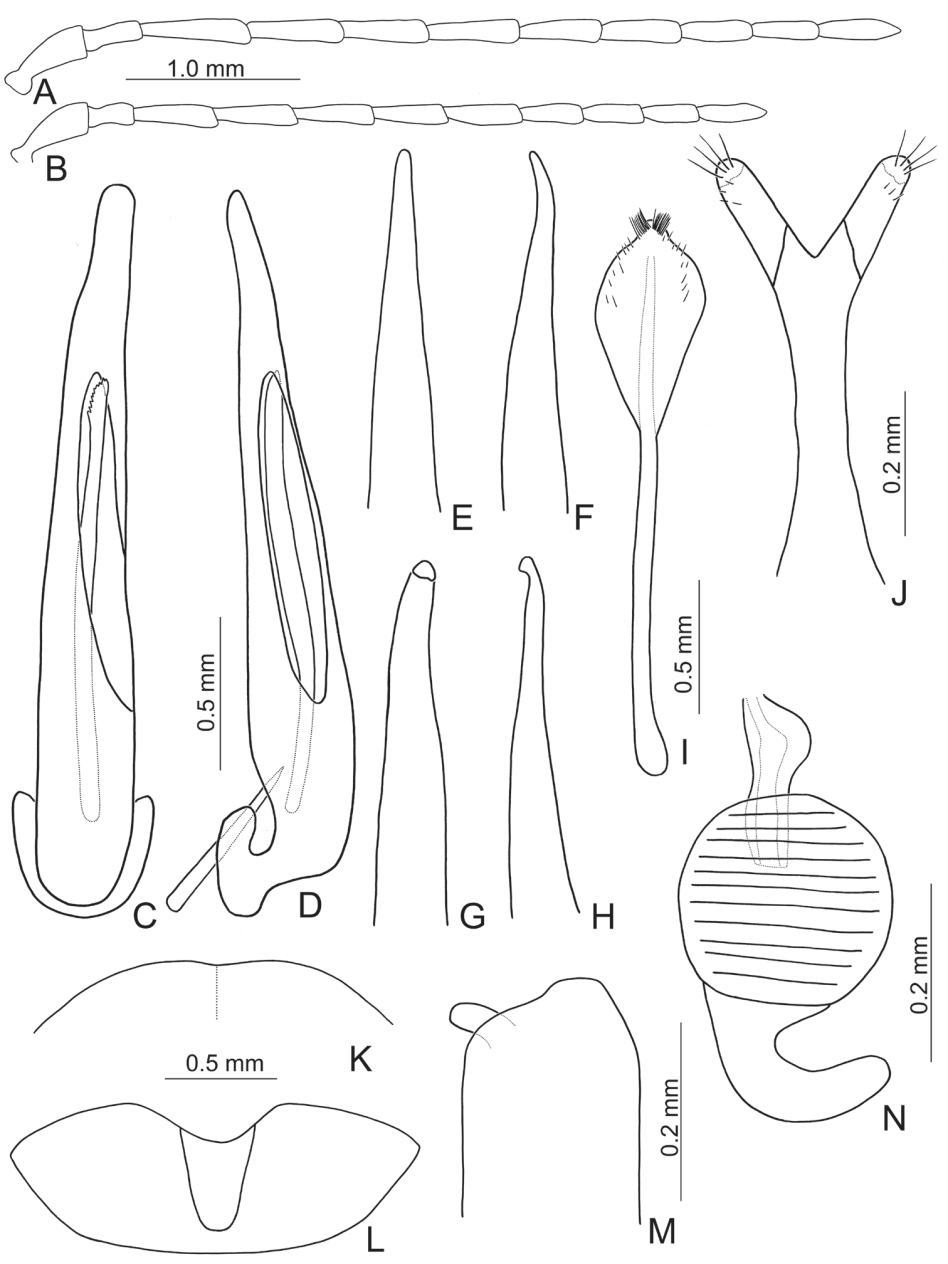
Diagnostic characters of *Pyrrhalta viridipennis* Kimoto **A** antenna, male **B** antenna, female **C** aedeagus, from Alishan (阿里山), dorsal view **D** ditto, lateral view **E** apex of aedeagus, from Shihfen (十分), dorsal view **F** ditto, lateral view **G** apex of aedeagus, from Hsiangyang, dorsal view **H** ditto, lateral view **I** abdominal ventrite VIII **J** gonocoxae **K** abdominal ventrite V, female **L** abdominal ventrite V, male **M** apex of tibia of middle leg **N** spermatheca.

Figs 11D–F , 13C, D , 14

Pyrrhalta viridipennis Kimoto, 1981: 2; Kimoto and Chu 1996: 57; Kimoto and Takizawa 1997: 301 (key), 374; Beenen 2010: 453 (catalogue); Xue and Yang 2010: 132 (catalogue); Yang et al. 2015: 122 (catalogue).

#### Types.

***Holotype*** ♂ (OMNH, by original designation): “NANSHANCHI [南山溪] / TAIWAN / 21. VII. 1974 / Y. KIYOYAMA [p, y] // Pyrrhalta / viridipennis / Kimoto, n. sp. [h, w] // HOLOTYPE [p, r] // PHOTO [p, r]”. ***Paratype***. 1 ♂ (KMNH): “(Taiwan) [p] / Sungan [松安] / Miaoli [h] Hsien [p, w] // 10.IV.1967 [h] / B. S. Chang [p, w] // Pyrrhalta / viridipennis / Kimoto, n. sp. [h, w] // Det. S. Kimoto, 19 [p, w] // PARATYPE [p, b]”.

#### Other material.

Taiwan. Chiayi: 1♂, 1♀ (TARI), Alishan (阿里山), 5–9.VIII.1981, leg. L. Y. Chou & S. C. Lin; Kaohsiung: 1♂ (TARI), Kuanshanyakou (關山啞口), 30.VII.2015, leg. C.-F. Lee; 1♂, 1♀ (TARI), Tengchih (藤枝), 7.IX.2012, leg. W.-C. Liao; 2♂, 5♀ (TARI), same but with “10.VIII.2013”; 1♀ (TARI), same but with “27.IX.2013”; 2♂, 4♀ (TARI), same but with “5.X.2013”; 4♂, 1♀ (TARI), same locality, 4.VIII.2012, leg. J.-C. Chen; 2♂, 2♀ (TARI), same locality, 30.VIII.2014, leg. B.-X. Guo; 2♀ (TARI), Tsuyunshan (出雲山), 25.IV.1990, leg. C. C. Chiang; Nantou: 1♀ (NMNS), Huishun (惠蓀), 3.VII.1991, leg. C. S. Lin; 1♂ (NMNS), Shanlinchi (杉林溪), 11.V.1990, leg. C. C. Chiang; 2♀ (TARI), Tsuifeng (翠峰), 1–3.VIII.1981, leg. T. Lin & W. S. Tang; 1♂, 1♀ (TARI), same locality, 1–3.IX.1982, leg. L. Y. Chou & K. C. Chou; 1♂, 2♀ (TARI), same locality, 12–14.IX.1984, leg. K. S. Lin & S. C. Lin; Pingtung: 1♂, 1♀ (TARI), Ali (阿禮), 30.V.2014, leg. J.-C. Chen; Taichung: 1♂, 1♀ (NHMUK), Basianshan National Forest Recreation Area (八仙山國家森林遊樂區), 27.V.2007, leg. G. Martin & D. L. J. Quicke; 2♀ (TARI), Tahsuehshan (大雪山), 21.IV.2010, leg. C.-F. Lee; Taipei: 1♂, 1♀ (TARI), Shihfen (十分), 23.V.2016, leg. J.-C. Chen; 1♂ (TARI), Yingtzuling (鶯子嶺), 21.VI.2016, leg. Y.-L. Lin; Taitung: 3♂ (TARI), Hsiangyang (向陽), 2.VII.2009, leg. S.-F. Yu; 8♂, 5♀ (TARI), same but with “leg. M.-H. Tsou”; 1♀ (TARI), same locality, 22.XII.2013, leg. W.-C. Liao; 1♂ (TARI), Hsiangyangshan (向陽山), 20.VI.2014, leg. J.-C. Chen.

#### Redescription.

Length 5.3–7.8 mm, width 2.3–3.5 mm. Body color (Fig. 11D–F) yellowish brown; antenna reddish brown; elytra green but with wide yellow stripe along lateral margin, apical area more or less yellowish brown. Eyes small, interocular space 2.67–2.75 × diameter of eye. Antennae filiform in males (Fig. 14A), length ratios of antennomeres I–XI 1.0: 0.7: 1.2: 1.1: 1.0: 1.0: 0.9: 0.9: 0.8: 0.7: 0.9, length to width ratios of antennomeres I–XI 2.6: 2.9: 4.5: 3.9: 3.7: 3.7: 3.4: 3.5: 3.1: 3.3: 4.4; similar in females (Fig. 14B), length ratios of antennomeres I–XI 1.0: 0.5: 0.9: 0.9: 0.9: 0.8: 0.8: 0.7: 0.7: 0.6: 0.8, length to width ratios of antennomeres I–XI 2.8: 2.1: 3.6: 4.0: 4.0: 3.6: 3.7: 3.2: 3.1: 2.9: 3.7. Pronotum and elytra convex. Pronotum 1.9–2.0 × wider than long, disc with reticulate microsculpture; with dense, coarse punctures, and short pubescence, with median longitudinal and lateral depressions; lateral margins rounded, widest at middle, apical and basal margin slightly concave; anterior and posterior setiferous punctures slightly erect. Elytra elongate and broad, parallel-sided, 1.6–1.7 × longer than wide; disc rough, with dense, fine punctures, and short pubescence. Apical spur of tibia of middle leg small and curved (Fig. 14M), and tarsomere I not modified in males. Aedeagus (Fig. 14C, D) slender in dorsal view, 7.9 × longer than wide, asymmetric, curved at apical 1/7, apically narrowed, apex broadly rounded; ostium large, not covered by membrane; straight but strongly curved near base in lateral view, slightly curved near apex, apex narrowly rounded; two endophallic sclerites elongate, primary sclerite longer and 0.6 × as long as aedeagus, apex with several fine teeth; secondary sclerite small, 0.4 × as long as the longer sclerite. Only apices of gonocoxae (Fig. 14J) sclerotized, elongate, with several short setae near apex, and four long setae near apex. Ventrite VIII (Fig. 14I) well sclerotized, strongly broadened near apex, outer sides strongly curved, several short setae along apical margin and bearing cluster of long setae medially, spiculum long. Receptacle of spermatheca (Fig. 14N) very swollen; pump short and strongly curved; sclerotized proximal spermathecal duct wide and short. Apical margin of abdominal ventrite V moderately concave medially, with deep, indistinctly margined depression at middle in males (Fig. 14L); slightly concave, with median, longitudinal internal ridge in females (Fig. 14K).

#### Variation.

The apex of the aedeagus is variable among populations; more slender in north and central Taiwan (Fig. 14E, F); recurved in southeast Taiwan (Fig. 14G, H).

#### Host plant.

Larvae and adults feed on leaves of Rhododendron rubropilosum Hayata var. rubropilosum Hayata (Ericaceae) (Fig. 13E, F).

#### Biology.

The first author and Mr. Ta-Hsiang Lee collected one larva feeding on leaves April 20, 2010 in Tahsuehshan, central Taiwan (Fig. 13E). It was reared in the laboratory. The newly eclosed adult appeared May 20 (Fig. 13F).

#### Remarks.

Adults of *P. viridipennis* Kimoto and *P. gressitti* Kimoto are both characterized by the green elytra with longitudinal ridges. However, *P. viridipennis* differs from *P. gressitti* by the larger body sizes, 5.3–7.8 mm long (3.9–5.4 mm long in *P. gressitti*), rough elytra with fine punctures (smooth and shining elytra with coarse punctures in *P. gressitti*); curved apex of aedeagus and narrowly rounded apex of primary endophallic slerite with teeth (Fig. 14C, D) (recurved apex of aedeagus and broadly rounded apex of primary endophallic sclerite lacking teeth in *P. gressitti* (Fig. 6C, D)); small and stout apical spur of tibia of middle leg in males (Fig. 14M) (slender apical spur of tibia of middle leg in males of *P. viridipennis* (Fig. 6F)); and slightly concave apical margin of abdominal ventrite V with short internal ridge in females (Fig. 14L) (moderately concave apical margin of abdominal ventrite V in females of *P. viridipennis* (Fig. 6J))

#### Distribution.

The species is widespread at mid-altitudes (1,500–2,500 m) in central and southern Taiwan.

### Pyrrhalta meifena

Taxon classificationAnimaliaColeopteraChrysomelidae

species group

121E152D-D91F-52CE-8FAF-13E3813384C0

#### Included species.

*Pyrrhalta alishanensis* sp. nov.; *P. igai* Kimoto, 1981; *P. meifena* Kimoto, 1976; and *P. meihuai* sp. nov.

#### Diagnosis.

Medium to large sized species (5.6–8.7 mm). Antenna stout, antennomeres VII-X shortest (1.5–2.2 × longer than wide), III–VI similar or slender. Body convex. Elytra relatively broad, 1.4–1.6 × longer than wide. Aedeagus apically tapering and symmetric (Figs 16C, 20C, 22C) except *P. meihuai* sp. nov. (Fig. 20C), ostium obliquely longitudinal and lacking cover; endophallic sclerites composed of primary sclerite with several teeth at apex in *P. igai* (Fig. 19C) and *P. meifena* (Fig. 20C), with one secondary sclerite in *P. meihuai* sp. nov. (Fig. 22C, D) or two secondary sclerites in *P. alishanensis* sp. nov. (Fig. 16C, D) Ventrite VIII in females apically sclerotized, apical margin widely rounded and with dense short setae; spiculum long (Figs 16E, 19E, 20E, 22E). Gonocoxae apically sclerotized and longitudinal, with dense, long setae along lateral and apical margins (Figs 16I, 19I, 20I, 22F). Apical margin of abdominal ventrite V with one pair of rounded ridges at middle and slightly concave between ridges in males (Figs 16H, 19H, 20H, 22I); widely rounded in females (Figs 16G, 20G, 22H) except concave in those of *P. igai* (Fig. 19G). Mesotibia lacking apical spine in males and tarsomere I not modified.

#### Biology.

Larvae and adults feed on leaves of *Acer* species (Sapindaceae).

### Pyrrhalta alishanensis sp. nov.

Taxon classificationAnimaliaColeopteraChrysomelidae

F607B635-125A-59B2-AD0D-AAA095412C21

http://zoobank.org/F257BF90-025E-4CC9-8B1B-836B74EDE26C

**Figure 15.**
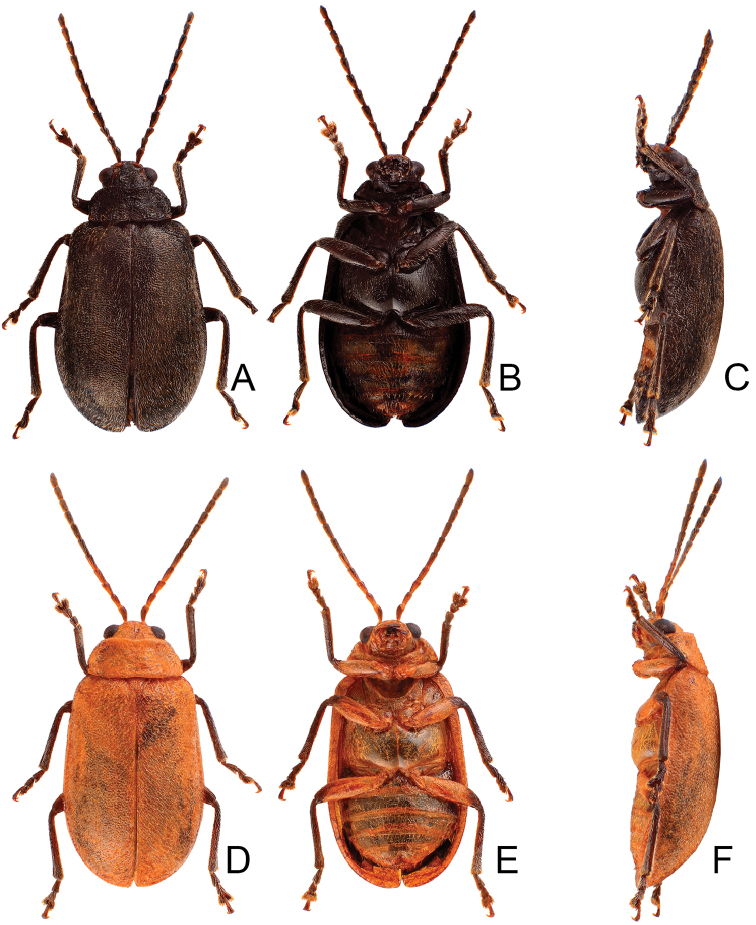
Habitus of *Pyrrhalta alishanensis* sp. nov. and *P. meifena* Kimoto **A***P. alishanensis* sp. nov., male, dorsal view **B** ditto, ventral view **C** ditto, lateral view **D***P. meifena*, male, dorsal view **E** ditto, ventral view **F** ditto, lateral view.

**Figure 16.**
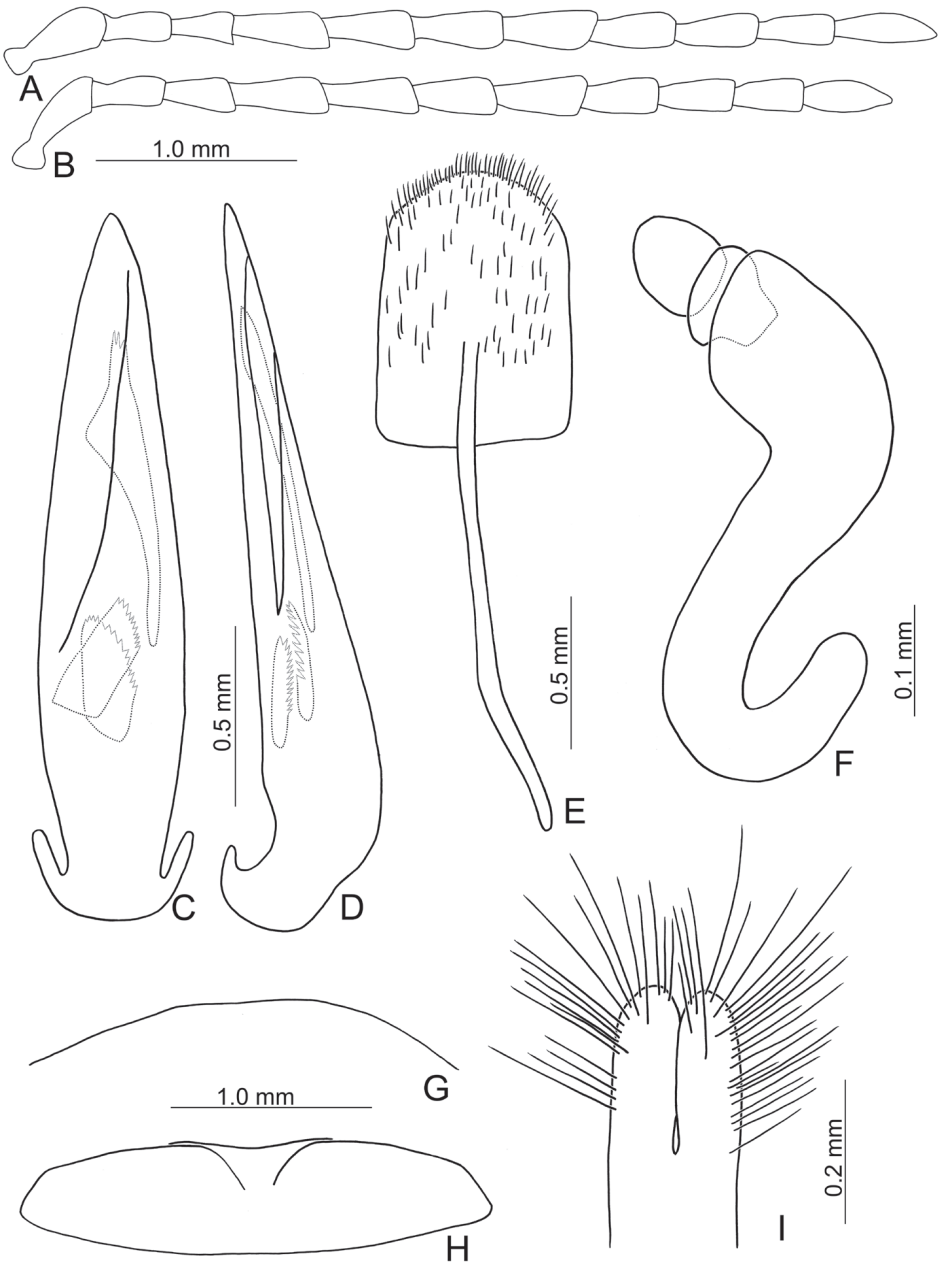
Diagnostic characters of *Pyrrhalta alishanensis* sp. nov. **A** antenna, male **B** antenna, female **C** aedeagus, dorsal view **D** ditto, lateral view **E** abdominal ventrite VIII **F** spermatheca **G** abdominal ventrite V, female **H** abdominal ventrite V, male **I** gonocoxae.

**Figure 17.**
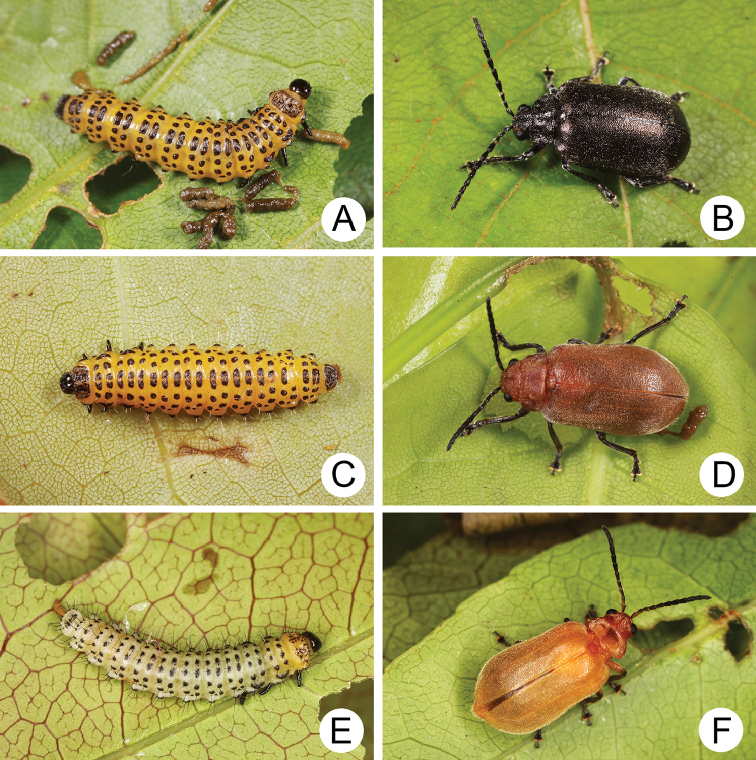
Field photographs of *Pyrrhalta alishanensis* sp. nov., *P. igai* Kimoto, and *P. meihuai* sp. nov. on host plant **A** mature larva of *P. alishanensis* sp. nov. **B** adult of *P. alishanensis* sp. nov. **C** mature larva of *P. igai***D** adult of *P. igai***E** mature larva of *P. meihuai* sp. nov. **F** adult of *P. meihuai* sp. nov.

Figs 15A–C , 16 , 17A, B

#### Types.

***Holotype*** ♂ (TARI), Taiwan. Chiayi: Alishan (阿里山), 22.IV.2009, leg. M.-H. Tsou. ***Paratypes*.** 7♂, 11♀ (TARI), same data as holotype.

#### Diagnosis.

Medium-sized species, 7.3–8.7 mm. Body black. Elytra with fine dense punctures.

#### Description.

Length 7.7–8.7 mm, width 3.8–4.6 mm. Body black (Fig. 15A–C); mouth parts black; abdominal ventrites yellow in males, ventrites II and III darker in females. Eyes small, interocular space 2.12–2.44 × diameter of eye. Antennae filiform in males (Fig. 16A), antennomeres V–VII broadest, length ratios of antennomeres I–XI 1.0: 0.6: 0.6: 0.8: 0.8: 0.8: 0.8: 0.7: 0.7: 0.7: 0.9, length to width ratios of antennomeres I–XI 2.5: 2.1: 1.9: 2.6: 2.4: 2.3: 2.3: 2.3: 2.5: 2.5: 3.5; similar in females (Fig. 16B), length ratios of antennomeres I–XI 1.0: 0.7: 0.6: 0.8: 0.8: 0.7: 0.8: 0.7: 0.7: 0.7: 0.8, length to width ratios of antennomeres I–XI 2.9: 2.4: 2.0: 2.5: 2.5: 2.1: 2.2: 2.1: 2.3: 2.2: 2.8. Pronotum and elytra convex. Pronotum 2.5–2.7 × wider than long, disc smooth and sparse short pubescence; and with extremely dense, coarse punctures laterally, reduced medially; with median longitudinal and lateral depressions; lateral margins moderately rounded, widest at middle, apical and basal margins slightly concave; anterior and posterior setiferous punctures slightly erect. Elytra broad, parallel-sided, 1.5–1.6 × longer than wide; disc smooth, with dense, coarse punctures, and short pubescence. Apical spur of tibia of middle leg absent and tarsomere I not modified in males. Aedeagus (Fig. 16C, D) broad in dorsal view, 4.6 × longer than wide, broadest at basal 2/5, slightly asymmetric, apically narrowed, apex acute; ostium obliquely longitudinal, not covered by a membrane; strongly curved near base in lateral view, apex narrowly rounded; primary endophallic sclerites elongate, 0.4 × as long as aedeagus, with several apical teeth, two secondary sclerites small and wide, 0.3 × as long as primary sclerite, with teeth along apical margins. Only apices of gonocoxae (Fig. 16I) sclerotized, longitudinal, with dense, long setae along lateral and apical margins. Ventrite VIII (Fig. 16E) well sclerotized, lateral margin slightly curved, with dense, long setae covering apex, spiculum long. Receptacle of spermatheca (Fig. 16F) slightly swollen; pump extremely long and strongly curved; sclerotized proximal spermathecal duct wide and short. Apical margin of abdominal ventrite V with one pair of rounded ridges at middle, slightly concave between ridges in males; truncate in females.

#### Remarks.

**Figure 18.**
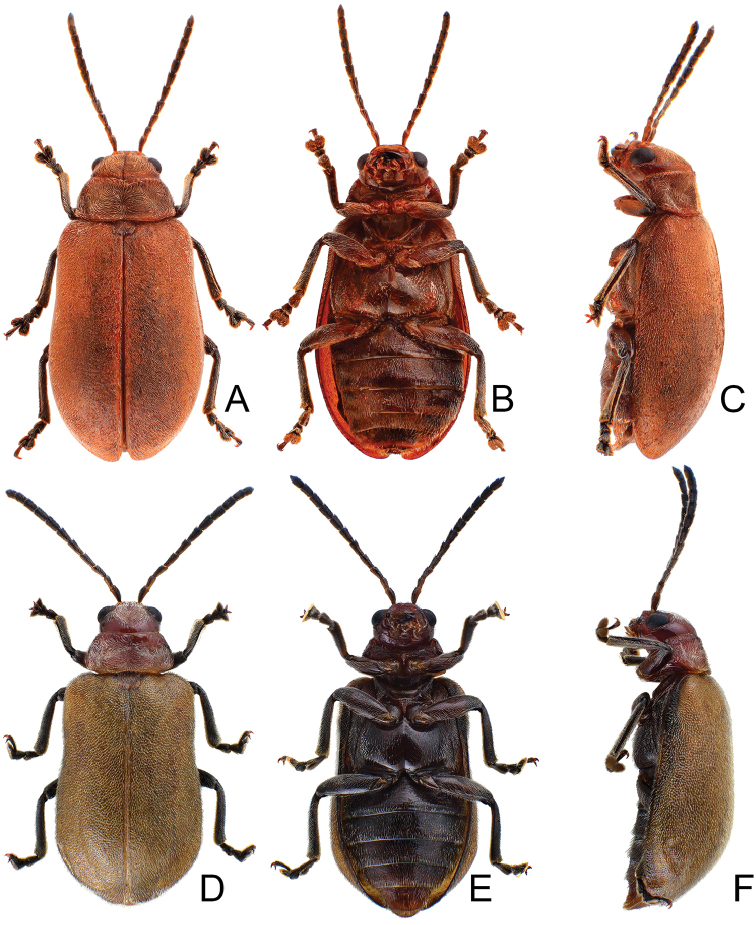
Habitus of *Pyrrhalta igai* Kimoto and *P. meihuai* sp. nov. **A***P. igai*, female, dorsal view **B** ditto, ventral view **C** ditto, lateral view **D***P. meihuai* sp. nov., female, dorsal view **E** ditto, ventral view **F** ditto, lateral view.

Adults of *P. alishanensis* sp. nov. are easily separated from other members of the species group by their black bodies (Fig. 15A–C) (yellow body in *P. meifena* Kimoto (Fig. 15D–F), brown body in *P. igai* Kimoto (Fig. 18 A–C) and *P. meihuai* sp. nov. (Fig. 18 D–F)). The lanceolate aedeagus is similar to those of *P. meifena* and *P. meihuai* sp. nov., but differs in possessing two secondary endophallic sclerites with toothed apical margins (Fig. 16C, D) (no secondary endophallic sclerites in *P. meifena* (Fig. 20C, D), one secondary endophallis sclerite with smooth apical margin in *P. meihuai* sp. nov. (Fig. 22C, D)). The elongate pump of the spermatheca is also diagnostic.

#### Host plant.

Larvae and adults feed on leaves of *Acer rubescens* Hayata (Sapindaceae) (Fig. 17A, B).

#### Biology.

The first author and Mr Mei-Hua Tsou found more than 20 mature larvae (Fig. 17A) on leaves of *Acer rubescens* Hayata in May 10, 2011, and reared them in the laboratory. Five pupae and one newly eclosed adult were observed June 25 (Fig. 17B).

#### Distribution.

Only known from the type locality.

#### Etymology.

Dedicated to the type locality, Alishan.

### Pyrrhalta igai

Taxon classificationAnimaliaColeopteraChrysomelidae

Kimoto, 1981

9918F771-8458-529A-B7AF-BAF3B94A49B8

**Figure 19.**
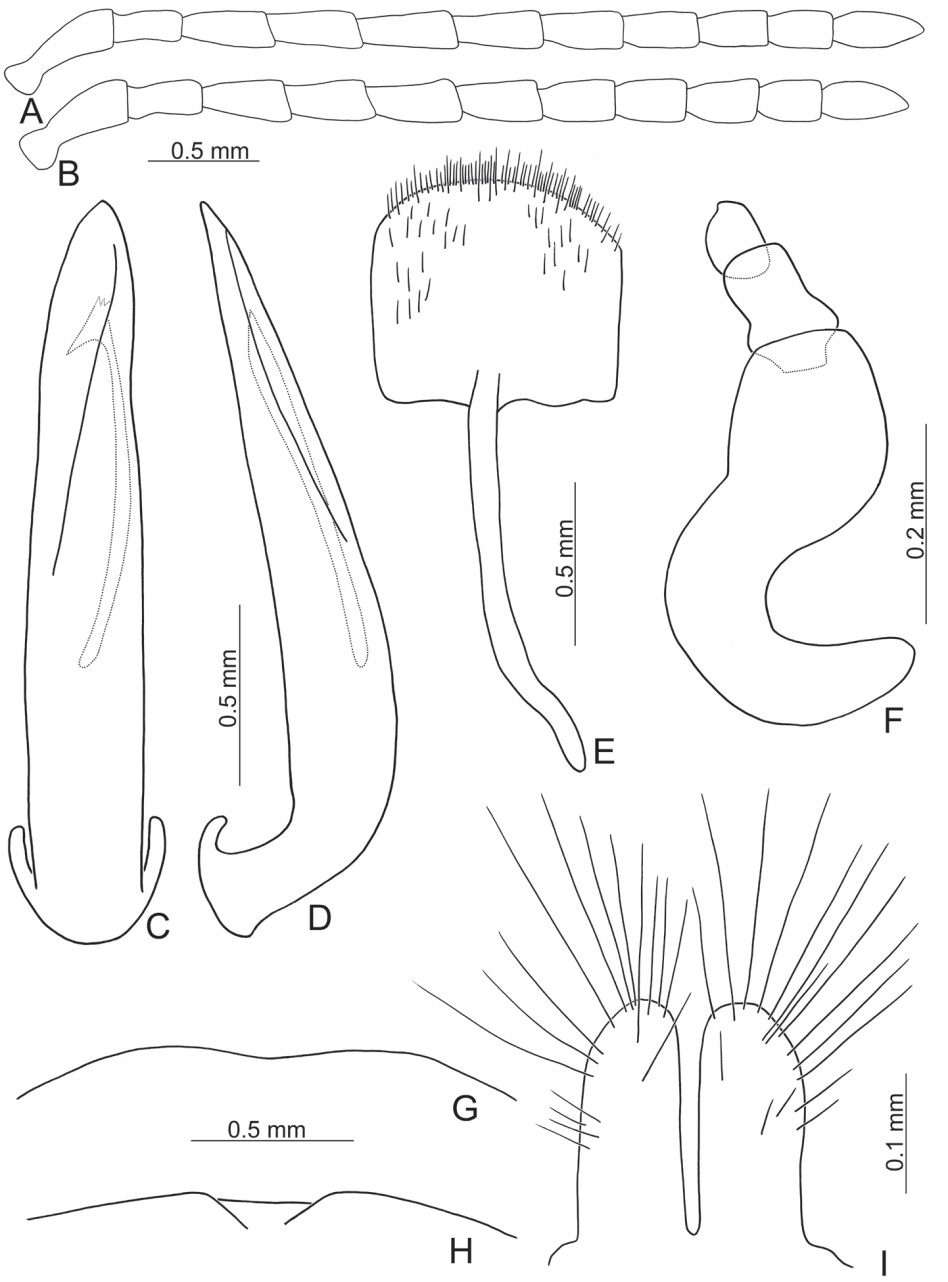
Diagnostic characters of *Pyrrhalta igai* Kimoto **A** antenna, male **B** antenna, female **C** aedeagus, dorsal view **D** ditto, lateral view **E** abdominal ventrite VIII **F** spermatheca **G** abdominal ventrite V, female **H** abdominal ventrite V, male **I** gonocoxae.

Figs 17C, D , 18A–C , 19

Pyrrhalta igai Kimoto, 1981: 1; Kimoto 1989a: 248 (additional records); Kimoto and Chu 1996: 56 (catalogue); Kimoto and Takizawa 1997: 300 (key), 373; Beenen 2010: 452 (catalogue); Xue and Yang 2010: 124 (catalogue); Yang et al. 2015: 117 (catalogue).

#### Types.

***Holotype*** ♂ (OMNH): “MUSHA [霧社] / FORMOSA / 25.V.1970 / A. RIN [p, y] // HOLOTYPE [p, r] // Pyrrhalta / igai / Kimoto, n. sp. [h, w] // PHOTO [p, r]”. ***Paratype*.** 1♂ (KMNH): “Mt. Shitoushan [獅頭山] / Miaoli Hsien / Taiwan / 3.VI.1976 / H. Makihara leg. [p, w] // Pyrrhalta / igai / Kimoto, n. sp. [h, w] // PARATYPE [p, b]”.

#### Other material.

Taiwan. Hsinchu: 1♂ (TARI), Talu trail (大鹿林道), 12.V.2018, leg. Y.-L. Lin; Kaohsiung: 3♂ (TARI), Neiyingshan (內英山), 5.V.2016, leg. B.-X. Guo; 1♀ (KMNH), Shyk Shan (石山), near Liu Kui (六龜), 28.VI.1986, leg. K. Baba, det. S. Kimoto, 1989; 3♂ (TARI), Tengchih (藤枝), 4.VII.2011, leg. M.-H. Tsou; 1♂ (TARI), 2.V.2015, leg. J.-C. Chen; Pingtung: 3♂, 7♀ (TARI), Machia (瑪家), 25.V.2016, leg. Y.-T. Chung; 1♂, 5♀ (TARI), Peitawushan (北大武山), 28.V.2014, leg. Y.-T. Chung; 1♂, 1♀ (TARI), Tahanshan (大漢山), 13.VI.2015, leg. W.-C. Liao; Taitung: 1♂, 2♀ (TARI), Litao (利稻), 23.IV.2011, leg. M.-H. Tsou; 4♂, 5♀ (TARI), Wulu (霧鹿), 18.IV.2011, leg. C.-F. Lee; 5♂, 3♀ (TARI), same locality, 21–27.IV.2011, leg. M.-H. Tsou; 1♂, 8♀ (TARI), same locality, 22.V.2011, leg. C.-F. Lee; Taoyuan: 2♀ (TARI), Paling (巴陵), 3–5.V.1983, leg. K. C. Chou & C. C. Pan; 1♀ (TARI), same locality, 26.V.2014, leg. M.-H. Tsou.

#### Redescription.

Length 8.1–8.5 mm, width 4.0–4.5 mm. Body brown (Fig. 18A–C); antennae and legs black. Eyes small, interocular space 3.20–3.33 × diameter of eye. Antennae filiform in males (Fig. 19A), gradually broadened from antennomere V to X, length ratios of antennomeres I–XI 1.0: 0.6: 0.7: 0.7: 0.8: 0.7: 0.7: 0.6: 0.6: 0.6: 0.8, length to width ratios of antennomeres I–XI 3.0: 2.1: 2.3: 2.3: 2.6: 2.3: 2.1: 2.2: 2.0: 1.8: 2.5; similar in females (Fig. 19B), length ratios of antennomeres I–XI 1.0: 0.6: 0.7: 0.7: 0.8: 0.7: 0.6: 0.6: 0.6: 0.5: 0.7, length to width ratios of antennomeres I–XI 2.6: 2.1: 2.0: 2.0: 2.2: 1.8: 1.8: 1.7: 1.7: 1.5: 2.3. Pronotum and elytra convex. Pronotum 2.4–2.5 × wider than long, disc with reticulate microsculpture; with extremely dense, coarse punctures, and long pubescence; with transverse ridge along apical margin deflexed at antero-lateral angles, ridge smooth, lacking pubescence but with sparse punctures; with median longitudinal and lateral depressions; lateral margins moderately rounded, widest in apical 1/3, apical and basal margins slightly concave; anterior and posterior setiferous punctures slightly erect. Elytra broad, parallel-sided, 1.4–1.5 × longer than wide; disc with reticulate microsculpture, with dense, coarse punctures, and short pubescence. Apical spur of tibia of middle leg absent and tarsomere I not modified in males. Aedeagus (Fig. 19C, D) slender in dorsal view, 6.8 × longer than wide, broadest at base, asymmetric, apically narrowed, curved at apical 1/5, apex acute; ostium obliquely longitudinal, not covered by a membrane; strongly curved near base in lateral view, apex narrowly rounded; primary endophallic sclerites elongate, 0.5 × as long as aedeagus, with three teeth at apex. Only apices of gonocoxae (Fig. 19I) sclerotized, longitudinal, with dense, long, setae along lateral and apical margins. Ventrite VIII (Fig. 19E) well sclerotized, with dense, long setae laterally, apical area, and along apical margin, spiculum long. Receptacle of spermatheca (Fig. 19F) very swollen; pump long and strongly curved; sclerotized proximal spermathecal duct wide and short. Apical margin of abdominal ventrite V with one pair of rounded ridges at middle, slightly concave between ridges in males (Fig. 19G) and females (Fig. 19H).

#### Remarks.

**Figure 20.**
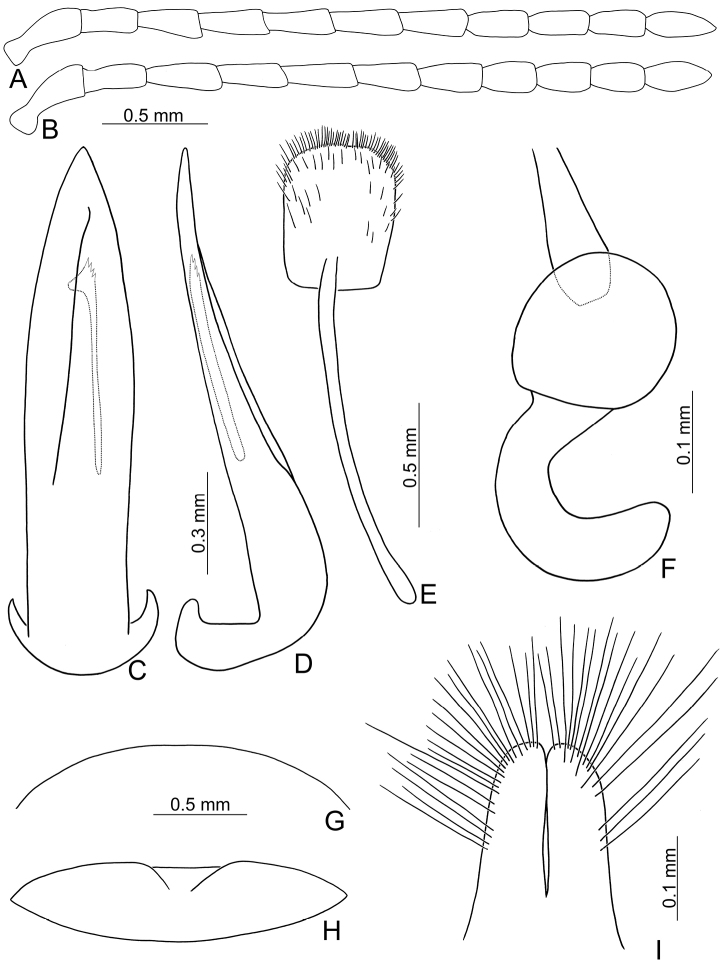
Diagnostic characters of *Pyrrhalta meifena* Kimoto **A** antenna, male **B** antenna, female **C** aedeagus, dorsal view **D** ditto, lateral view **E** abdominal ventrite VIII **F** spermatheca **G** abdominal ventrite V, female **H** abdominal ventrite V, male **I** gonocoxae.

Adults of *P. igai* Kimoto are similar to those of *Pyrrhalta meihuai* sp. nov. in body sizes and color patterns (Fig. 17D, F), but differ by the rough pronotum and elytra covered with reticulate microsculpture (shining and smooth pronotum and elytra in *P. meihuai* sp. nov.). In males of *P. igai*, the single endophallic sclerites (Fig. 19C) are shared with those of *P. meifena* (Fig. 20C), and the aedeagus is characterized by its asymmetrical shape (lanceolate shape in others of the species group (Figs 16C, 20C, 22C).

#### Host plant.

Larvae and adults feed on leaves of *Acer albopurpurascens* Hayata (Sapindaceae).

#### Biology.

The first author and Mr Mei-Hua Tsou found larvae feeding on leaves (Fig. 17C) March 29, 2009 in Wulu, Southeast Taiwan. They were transferred to the laboratory for rearing. mature larvae began to burrow into soil March 30, and built underground chambers for pupation. Duration of the pupal stage was 19–23 days. adults (Fig. 17D) appeared in spring and summer.

#### Remarks.

The collecting data of the label on the holotype is inconsistent with Kimoto (1981) probably because of typos.

#### Distribution.

The species is widespread at mid-altitudes (1,500–2,500 m) in Taiwan.

### Pyrrhalta meifena

Taxon classificationAnimaliaColeopteraChrysomelidae

Kimoto, 1976

50C34370-AF51-5EE7-A2F8-F944EC9460C1

**Figure 21.**
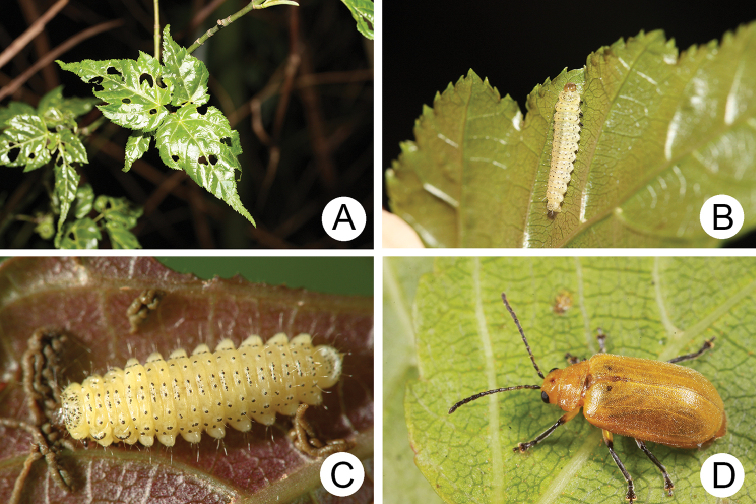
Field photographs of *Pyrrhalta meifena* Kimoto on host plant **A** host plant, Acer insulare var. caudatifolium**B** Second-instar larva **C** third-instar larva **D** adult.

Figs 15D–F , 20 , 21

Pyrrhalta meifena Kimoto, 1976: 4; Kimoto 1987: 188 (additional records); Kimoto and Chu 1996: 56 (catalogue); Kimoto and Takizawa 1997: 3010 (key), 373; Beenen 2010: 453 (catalogue); Xue and Yang 2010: 126 (catalogue); Lee and Cheng 2010: 119 (redescription); Takahashi 2012: 323 (deposition of type material); Yang et al. 2015: 119 (catalogue).

#### Types.

***Holotype*** ♂ (OMNH, by original designation): “[TAIWAN] / Meifeng [梅峰] / Nantou Hsien [h, w] // 26.VI.1971/ Y. Miyatake [h, w] // Pyrrhalta / meifena / Kimoto, n. sp. [h, w] // HOLOTYPE [p, r] // (Regd. O.M.N.H.) [p, w]”. ***Paratypes*.** 1 ♀ (KMNH): “(Taiwan) / Wushe [霧社] / Nantou Hsien [h, w] // 30.V.1965 / B. S. Chang [h, w] // Pyrrhalta / meifena / Kimoto, n. sp. [h, w] // Det. S. Kimoto, 19 [p, w] // PARATOPOTYPE [p, b]”; 1 ♂ (KMNH): “[TAIWAN] / Meifeng [梅峰] / Nantou Hsien [h, w] // 26.VI.1971 / Y. Miyatake [h, w] // Pyrrhalta / meifena / Kimoto, n. sp. [h, w] // PARATOPOTYPE [p, b]”.

#### Other material.

Taiwan. Chiayi: 1♂, 2♀ (TARI), Alishan (阿里山), 22.IX.2009, leg. M.-H. Tsou; 2♂♂ (TARI), same locality, 29.V.2016, leg. Y.-T. Chung; 1♂ (TARI), Shizilu (十字路), 23.VII.2015, leg. U. Ong; Hsinchu: 9♂, 2♀, Litungshan (李棟山), 24–25.III.2009, leg. M.-H. Tsou; 4♂, 3♀ (TARI), same locality, 28.III.2009, leg. S.-F. Yu; 1♀ (TARI), same locality, 16.VI.2010, leg. Y.-L. Lin; Hualien: 1♂, 2♀ (TARI), Kuanyuan (關原), 2.VII.2008, leg. S.-F. Yu and M.-H. Tsou; 1♀ (TARI), Pilu (碧綠), 15.VII.2019, leg. B.-X. Guo; 1♀ (TARI), Tayuling (大禹嶺), 2.VII.2018, leg. J.-C. Chen; Kaohsiung: 1♀ (TARI), Chungchihkung (中之關), 1.VII.2009, leg. S.-F. Yu; 1♀ (TARI), same locality, 2.VII.2009, leg. M.-H. Tsou; 1♀ (TARI), same locality, 17.IV.2012, leg. L.-P. Hsu; 1♂, 1♀ (TARI), Tienchih (天池), 2.VII.2009, leg. M.-H. Tsou; Nantou: 1♂, 2♀ (TARI), Meifeng (梅峰), 24–26.VI.1981, leg. K. S. Lin & W. S. Tang; 1♀ (TARI), same locality, 1–3.VIII.1981, leg. T. Lin & W. S. Tang; 6♂ (TARI), Tatachia (塔塔加), 13.VII.2014, leg. W.-C. Liao; 1♂ (TARI), Tsuifeng (翠峰), 25–27.VI.1981, leg. K. S. Lin & W. S. Tang; 1♀ (TARI), same locality, S. C. Lin & C. N. Lin; Taichung: 5♂, 7♀ (TARI), Anmashan (鞍馬山), 7.VI.2010, leg. C.-F. Lee; Taitung: 1♂ (TARI), Hsiangyang (向陽), 9.V.2013, leg. J.-C. Chen; 1♂ (TARI), same locality, 18.VII.2014, leg. W.-C. Huan; 2♀ (TARI), Liyuan (栗園), 19.VI.2013, leg. C.-F. Lee.

#### Redescription.

Length 5.6–6.5 mm, width 2.7–3.2 mm. Body yellow (Fig. 15D–F); antennae black but four or five basal antennomeres paled; tibiae and tarsi black or blackish brown. Eyes small, interocular space 2.35–3.58x diameter of eye. Antennae filiform in males (Fig. 20A), gradually broadened from antennomere V to X, length ratios of antennomeres I–XI 1.0: 0.7: 0.7: 0.8: 0.8: 0.8: 0.8: 0.7: 0.7: 0.7: 0.8, length to width ratios of antennomeres I–XI 2.8: 2.2: 2.3: 2.5: 2.6: 2.7: 2.6: 2.4: 2.3: 2.1: 2.7; similar in females (Fig. 20B), length ratios of antennomeres I–XI 1.0: 0.7: 0.8: 0.7: 0.9: 0.7: 0.7: 0.6: 0.6: 0.6: 0.8, length to width ratios of antennomeres I–XI 2.7: 2.5: 2.8: 2.7: 2.8: 2.3: 2.1: 1.8: 1.8: 1.9: 2.3. Pronotum and elytra convex. Pronotum 2.2–2.3 × wider than long, disc smooth; with extremely dense, coarse punctures, and sparse long pubescence, with median longitudinal and lateral depressions; lateral margins moderately rounded, widest at middle, apical and basal margins slightly concave; anterior and posterior setiferous punctures slightly erect. Elytra broad, parallel-sided, 1.5–1.6 × longer than wide; disc smooth, with extremely dense, coarse punctures, and short pubescence. Apical spur of tibia of middle leg absent and tarsomere I not modified in males. Aedeagus (Fig. 20C, D) broad in dorsal view, 5.7 × longer than wide, symmetric, subparallel from base to middle, then apically narrowed, apex acute; ostium obliquely longitudinal, covered by a membrane; strongly curved near base in lateral view, slight recurved at apical 1/6, apex acute; primary endophallic sclerites elongate, 0.5 × as long as aedeagus, with several teeth at apex. Only apices of gonocoxae (Fig. 20I) sclerotized, longitudinal, with dense, long setae along lateral and apical margins. Ventrite VIII (Fig. 20E) well sclerotized, with dense, long setae laterally, apical area, and along apical margin, spiculum long. Receptacle of spermatheca (Fig. 20F) very swollen; pump long and strongly curved; sclerotized proximal spermathecal duct narrow and short. Apical margin of abdominal ventrite V with one pair of rounded ridges at middle and slightly concave between ridges in males (Fig. 20H); broadly rounded in females (Fig. 20G).

#### Remarks.

**Figure 22.**
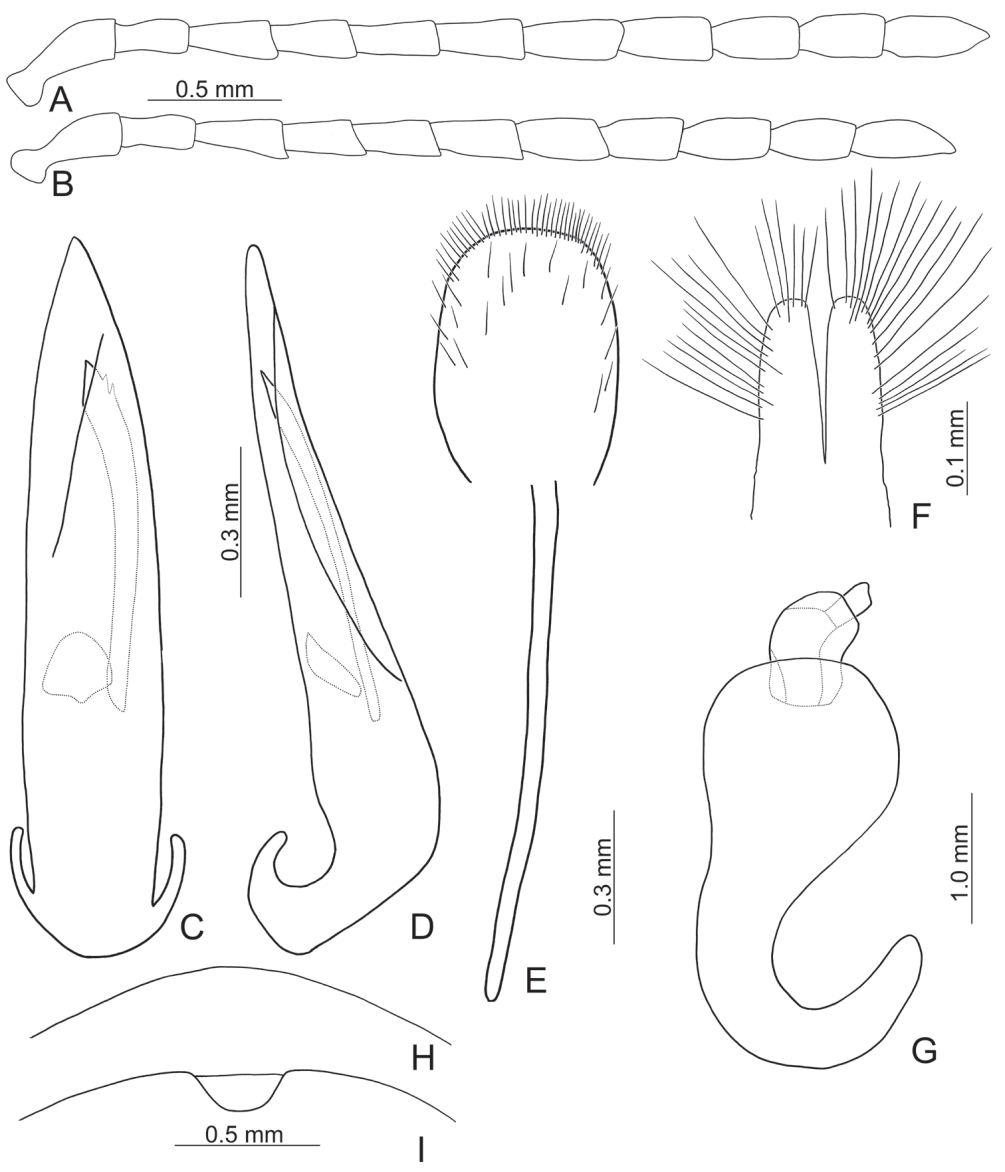
Diagnostic characters of *Pyrrhalta meihuai* sp. nov. **A** antenna, male **B** antenna, female **C**aedeagus, dorsal view **D** ditto, lateral view **E** abdominal ventrite VIII **F** gonocoxae **G** spermatheca **H** abdominal ventrite V, female **I** abdominal ventrite V, male.

adults of *P. meifena* Kimoto are characterized by their small body sizes, 5.6–6.5 mm long (7.3–8.7 mm long in others), and yellow bodies (Fig. 15D–F) (black bodies in *P. alishanensis* sp. nov. (Fig. 15A–C); brown bodies in *P. igai* and *P. meihuai* sp. nov. (Fig. 18)) within the species group. In males of *P. meifena*, the lanceolate aedeagus (Fig. 20C) is similar to those of *P. alishanensis* sp. nov. (Fig. 16C) and *P. meihuai* sp. nov. (Fig. 22C) but differs in lacking secondary endophallic sclerite in addition to the primary endophallic sclerite (one secondary sclerite in *P. meihuai* sp. nov.; two secondary sclerites in *P. alishanensis* sp. nov.).

#### Host plants.

Larvae and adults feed on leaves of Acer insulare Hayata var. caudatifolium (Hayata) and *A. rubescens* Hayata (Sapindaceae).

#### Biology.

Mrs Su-Fang Yu found young larvae (Fig. 21B) feeding on tender leaves of Acer insulare var. caudatifolium (Fig. 21A) February 26, 2009, in Litungshan, northern Taiwan; and reared them in the laboratory. mature larvae (Fig. 20C) began to burrow into soil March 2, and built underground chambers for pupation. Duration of the pupal stage was 22–24 days. adults (Fig. 21D) appeared from spring to summer.

#### Distribution.

The species is widespread at mid-altitudes (1,500–2,500 m) in Taiwan.

### Pyrrhalta meihuai sp. nov.

Taxon classificationAnimaliaColeopteraChrysomelidae

365746A6-8CB5-577A-B511-F6D824927EC2

http://zoobank.org/0D75E209-97C3-4F69-A865-B056DAE8BF64

Figs 17E, F , 18D–F , 22

#### Types.

***Holotype*** ♂ (TARI), Taiwan. Ilan: Mingchi (明池), 2.V.2009, leg. M.-H. Tsou. ***Paratypes*.** 3♂, 3♀ (TARI), same data as holotype; 1♂, 3♀ (TARI), same but with “1.V.2009”; Pingtung: 1♂ (TARI), Tahanshan (大漢山), 18.VI.2012, leg. Y.-T. Chung; 1♂ (TARI), same but with “11.VII.2012”; 2♂, 1♀ (TARI), same but with “24.IV.2013”; 3♂, 5♀ (TARI), same but with “15.V.2013”; 2♂ (TARI), same but with “25.V.2013”; 2♀ (TARI), same but with “30.V.2013”; 3♀ (TARI), same but with “17.VI.2013”; 3♂ (TARI), same but with “2.VII.2013”; 1♂ (TARI), same but with “10.VII.2013”; 1♀ (TARI), same but with “30.VII.2013”; 1♀ (TARI), same but with “12.VI.2014”; 1♀ (TARI), same but with “4.VI.2020”; 1♀ (TARI), same locality, 19.VII.2012, leg. C.-F. Lee; 1♂ (TARI), Tahantrail (大漢林道), 20.VIII.2012, leg. J.-C. Chen; 1♀ (TARI), same but with “27.V.2013”; Taipei: 1♀ (TARI), Hsiungkungshan (熊空山), 15.VI.2014, leg. Y.-L. Lin; Taitung: 1♀ (TARI), Lichia (利嘉), 15.VII.2014, leg. Y.-T. Chung; 1♂ (TARI), same but with “16.VII.2014”; Taoyuan: 1♂ (TARI), Hsiaowulai (小烏來), 29.IX.2009, leg. M-H. Tsou; 1♀ (TARI), same locality, 1.VI.2010, leg. S.-F. Yu; 1♀ (TARI), Lalashan (拉拉山), 4.V.2009, leg. H.-J. Chen; 1♂ (TARI), Tungyanshan (東眼山), 12.VII.2015, leg. H. Lee.

#### Diagnosis.

Medium-sized species, 7.3–8.7mm. Body brown. Elytra with fine dense punctures. Discs of pronotum and elytra smooth, lacking reticulate microsculpture.

#### Redescription.

Length 7.3–8.6 mm, width 3.4–4.0 mm. Head and prothorax reddish brown (Fig. 18D–F), but antennae black; scutellum and elytra yellowish brown; meso- and metathoracic ventrites, and legs black. Eyes small, interocular space 2.24–2.76 × diameter of eye. Antennae filiform in males (Fig. 22A), gradually broadened from antennomere V to X, length ratios of antennomeres I–XI 1.0: 0.6: 0.7: 0.7: 0.8: 0.7: 0.8: 0.8: 0.7: 0.7: 0.9, length to width ratios of antennomeres I–XI 2.8: 2.1: 2.2: 2.2: 2.6: 2.2: 2.4: 2.3: 2.0: 1.9: 2.7; similar in females (Fig. 22B), length ratios of antennomeres I–XI 1.0: 0.6: 0.8: 0.6: 0.7: 0.7: 0.8: 0.7: 0.8: 0.7: 0.9, length to width ratios of antennomeres I–XI 2.7: 2.2: 2.2: 2.0: 2.1: 2.2: 2.1: 1.9: 2.2: 2.0: 2.8. Pronotum and elytra convex. Pronotum 2.2–2.3 × wider than long, disc smooth; with extremely dense, coarse punctures, and long pubescence, with median longitudinal and lateral depressions; lateral margins slightly rounded, widest at middle, apical margin slightly concave, basal margin straight; anterior and posterior setiferous punctures not erect. Elytra broad, parallel-sided, 1.6 × longer than wide; disc smooth, with dense, coarse punctures, and short pubescence. Apical spur of tibia of middle leg absent and tarsomere I not modified in males. Aedeagus (Fig. 22C, D) broad in dorsal view, 5.4 × as long as aedeagus, with several teeth along lateral margin near apex, secondary sclerite small and wide, 0.2 × as long as primary sclerite. Only apices of gonocoxae (Fig. 22F) sclerotized, longitudinal, with dense, long setae along lateral and apical margins. Ventrite VIII (Fig. 22E) well sclerotized, sides strongly curved, with dense, long setae laterally, apical area, and along apical margin, spiculum extremely long. Receptacle of spermatheca (Fig. 22G) very swollen; pump long and strongly curved; sclerotized proximal spermathecal duct wide and short. Apical margin of abdominal ventrite V broadly rounded with deep depression at middle in males (Fig. 22I); while lacking depression in females (Fig. 22H).

#### Remarks.

Adults of *P. meihuai* sp. nov. are similar to those of *P. igai* Kimoto in body sizes and color patterns (Fig. 17D, F), but differ by the shining, smooth pronotum and elytra (rough pronotum and elytra covered by reticulate microsculpture in *P. igai*). In males of *P. meihuai* sp. nov., the lanceolate aedeagus (Fig. 22C) is similar to that of *P. alishanensis* sp. nov. (Fig. 16C) and *P. meifena* (Fig. 20C) but differs in possessing one secondary endophallic sclerite in addition to the primary endophallic sclerite (no secondary sclerite in *P. meifena*; two secondary sclerites in *P. alishanensis* sp. nov.).

#### Host plant.

Larvae and adults feed on leaves of *Acer serrulatum* Hayata (Sapindaceae).

#### Biology.

Mr Mei-Hua Tsou collected mature larvae (Fig. 14E) April 5, 2009 in Mingchi, Northeast Taiwan. They began burrowing into soil at the same day, and built underground chambers for pupation. Duration of the pupal stage was 24–27 days. adults (Fig. 17F) appeared from spring to summer.

#### Distribution.

The species is widespread at mid-altitudes (1,500–2,500 m) in Taiwan.

#### Etymology.

Dedicated to Mr. Mei-Hua Tsou. He, the first author, and Mr. Hou-Jay Chen were the first to collect larvae of this new species and rear them successfully to adults.

### Pyrrhalta semifulva

Taxon classificationAnimaliaColeopteraChrysomelidae

species group

F3A641DF-B6F4-528B-BAB1-E012F1B717C3

#### Included species.

*Pyrrhalta maculata* Gressitt & Kimoto, 1963; *P. tsoui* Bezděk & Lee, 2019; *P. formosanensis* sp. nov.; *P. semifulva* (Jacoby, 1885); *P. discalis* Gressitt & Kimoto, 1963; *P. ishiharai* Kimoto, 1976; and *P. wulaiensis* sp. nov.

#### Diagnosis.

Small sized species (3.3–5.6 mm). Antenna stout, antennomeres VIII–X stout (1.4–2.0x longer than wide), III-VI slender. Body convex. Elytra relatively wider, 1.4–1.6 × longer than wide. Aedeagus asymmetric; ostium covered by a membrane or lacking cover; endophallic sclerites composed of two slender sclerites, with several teeth on apex of primary sclerite and with one additional tooth near apex of secondary sclerite except *P. formosanensis* sp. nov. with only primary sclerite (Fig. 28D–F), *P. maculata* lacking teeth on sclerites (Fig. 24C, D), *P. semifulva* (Fig. 29C, D) and *P. discalis* (Fig. 32C, D) lacking additional tooth on secondary sclerite. The ventrite VIII in females apically sclerotized, with dense short and long setae mixed along apical margin; spiculum long (Figs 24F, 25F, 28H, 29G, 32E, 35F, 36E). Gonocoxae apically sclerotized and with variable number of setae; both gonocoxae small and connected, with two long setae on each gonocoxa in *P. maculata* (Fig. 24H) and *P. tsoui* (Fig. 25J), both gonocoxae longitudinally oriented and connected, with a number of setae near apices in *P. discalis* (Fig. 32I), gonocoxae longitudinal and with dense, long setae in *P. ishiharai* (Fig. 35K), both gonocoxae separated, transverse, and disc with a number of scattered short setae in *P. wulaiensis* sp. nov. (Fig. 36I). Both gonocoxae separated, small and with dense short setae in *P. formosanensis* sp. nov. (Fig. 28J) and *P. semifulva* (Fig. 29K). Apical margin of abdominal ventrite V truncate or slightly concave, with deeply rounded depression at middle in males(Figs 24J, 25I, 28K, 29J, 32H, 35J, 36H); slightly concave or widely rounded in females (Figs 24I, 25H, 28L, 29I, 32G, 35I, 36G). Mesotibia with apical spine in males of *P. maculata* (Fig. 24E), *P. tsoui* (Fig. 25E), *P. formosanensis* sp. nov. (Fig. 28G), and *P. ishiharai* (Fig. 35E); or lacking apical spine in those of the remaining species; mesotarsi with tarsomere I modified in males of *P. maculata* (Fig. 24K), *P. formosanensis* sp. nov. (Fig. 38M), and *P. ishiharai* (Fig. 35H).

#### Remarks.

**Figure 23.**
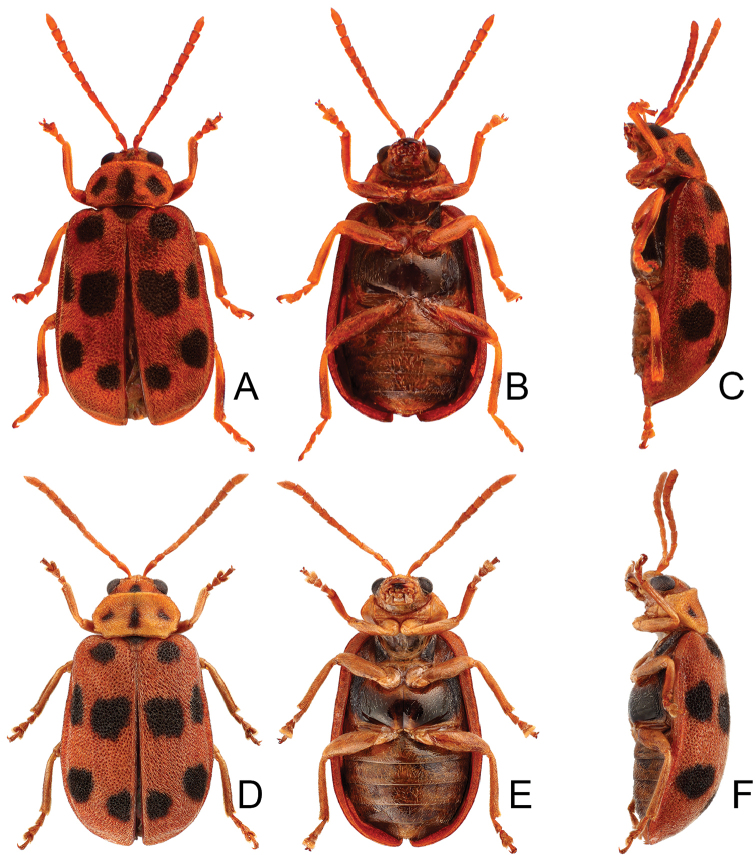
Habitus of *Pyrrhalta maculata* Gressitt & Kimoto and *P. tsoui* Bezděk & Lee **A***P. maculata*, female, dorsal view **B** ditto, ventral view **C** ditto, lateral view **D***P. tsoui* female, dorsal view **E** ditto, ventral view **F** ditto, lateral view.

**Figure 24.**
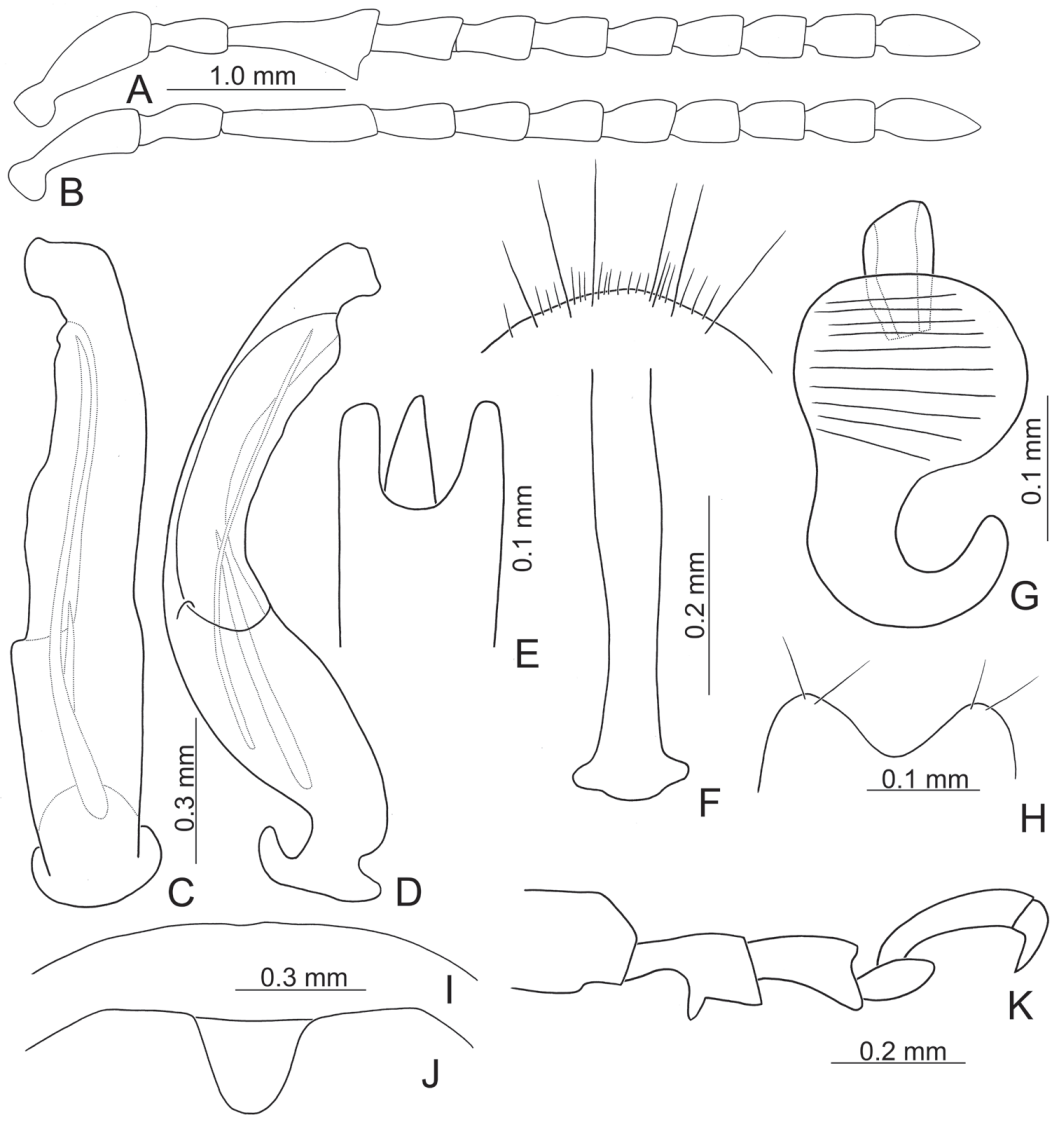
Diagnostic characters of *Pyrrhalta maculata* Gressitt & Kimoto **A** antenna, male **B** antenna, female **C** aedeagus, dorsal view **D** ditto, lateral view **E** apex of tibia of middle leg, male **F** abdominal ventrite VIII **G** spermatheca **H** gonocoxae **I** abdominal ventrite V, female **J** abdominal ventrite V, male **K** tarsi of middle leg, male.

**Figure 25.**
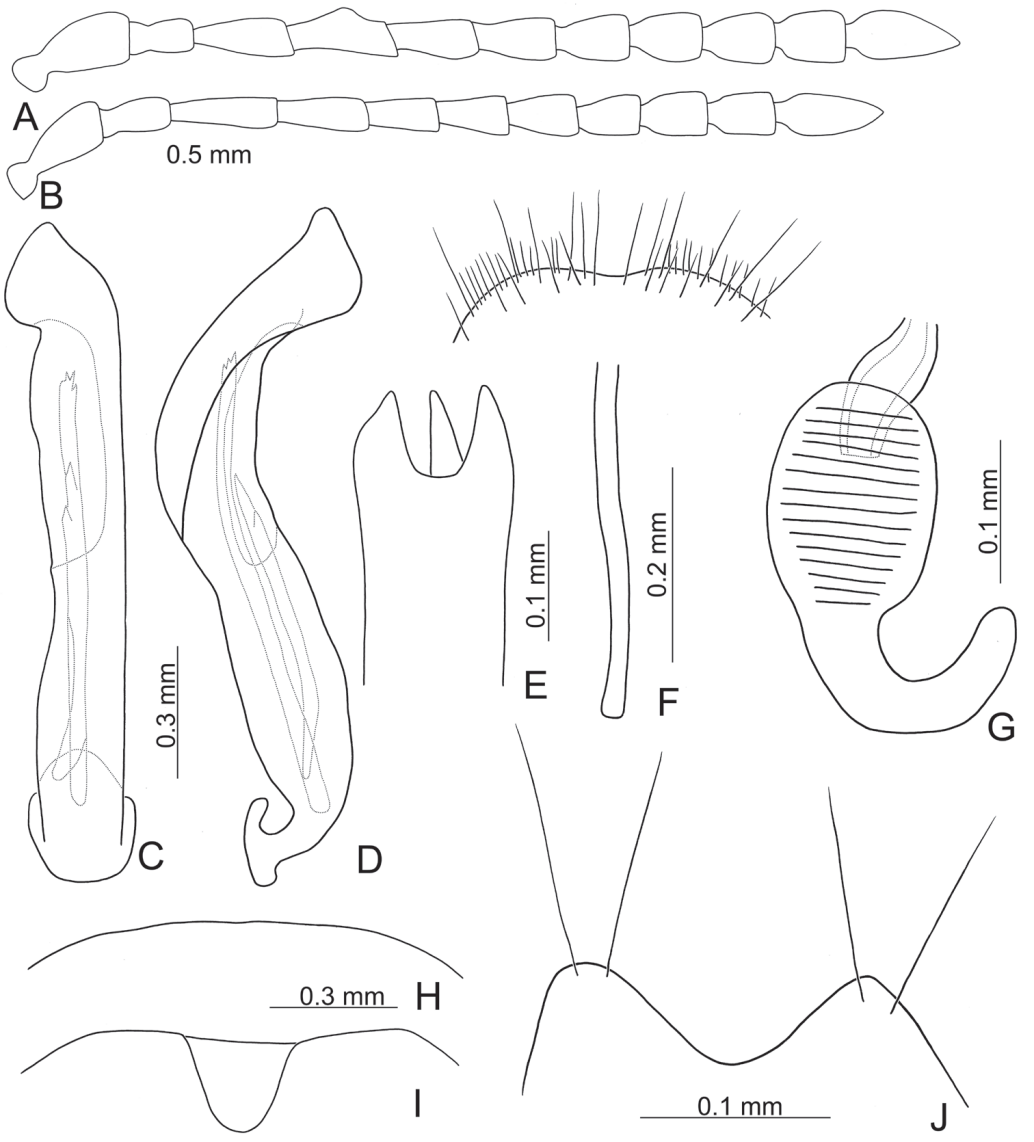
Diagnostic characters of *Pyrrhalta tsoui* Bezděk & Lee **A** antenna, male **B** antenna, female **C** aedeagus, dorsal view **D** ditto, lateral view **E** apex of tibia of middle leg, male **F** abdominal ventrite VIII **G** spermatheca **H** abdominal ventrite V, female **I** abdominal ventrite V, male **J** gonocoxae.

Included species can be subdivided into species complexes based on similar color patterns. For example, Bezděk and Lee (2019) treated the *P. maculata* species complex, including *P. maculata*, *P. tsoui*, and five more species. They are characterized by their maculate elytra (Fig. 23), strongly curved aedeagi (Figs 24C, D, 25C, D), and only two setae on each gonocoxa (Figs 24H, 25J). *Pyrrhalta semifulva* and *P. formosanensis* sp. nov. belong to another species complex characterized by their reddish brown elytra (Fig. 27) and small gonoxae possessing dense setae (Figs 28J, 29K). *Pyrrhalta ishiharai* is grouped with *P. wulaiensis* sp. nov. based on the longitudinal ridges of the elytra (Fig. 34A, D) and angular apices of aedeagi (Figs 35C, 36C).

#### Biology.

Anthophagous species. Larvae and adults feed on flowers of *Meliosma rhoifolia* (Sabiaceae) or species of Rosaceae.

### Pyrrhalta maculata

Taxon classificationAnimaliaColeopteraChrysomelidae

Gressitt & Kimoto, 1963

2FD5CEC4-6B12-50F0-A452-BC5EE112ED5C

Figs 23A–C , 24

Pyrrhalta maculata Gressitt & Kimoto, 1963: 456; Kimoto 1969: 28 (additional records in Taiwan); Kimoto 1987: 188 (additional records in Taiwan); Kimoto 1989a: 248 (additional records in Taiwan); Kimoto 1991: 9 (additional records in Taiwan); Kimoto and Chu 1996: 56 (catalogue); Kimoto and Takizawa 1997: 300 (key), 373; Yang 2002: 627 (China: Fujian); Beenen 2010: 453 (catalogue); Xue and Yang 2010: 126 (catalogue); Medvedev 2013: 268 (key); Yang et al. 2015: 118 (catalogue); Bezděk and Lee 2019: 519 (redescription).
Pyrrhalta (Pyrrhalta) maculata : Wilcox, 1971: 88 (catalogue).

#### Type

(types examined by Bezděk and Lee (2019) exclude)**. *Paratype*.** 1♂ (CAS): “TAIWAN (C.), Mu- / sha (Wuse) 1100 m, / V-19-32. Gressitt [p, w] // PARATYPE / Pyrrhalta / maculata [h] / Gressitt and Kimoto [p, y]”.

#### Other material

(specimens examined by Bezděk and Lee (2019) exclude). Taiwan. Nantou: 1♀ (TARI), Huakang (華岡), 24.IV.2019, leg. J.-C. Chen; 1♂, 3♀♀ (NMNS), Meifeng (梅峰), 9.IV. –7.V.2002, leg. C. S. Lin & W. T. Yang; 1♀ (NMNS), same but with “7.V. –11.VI.2002”; 1♀ (NMNS), same but with “11.VI. –9.VII.2002”; 1♂ (NMNS), same but with “10.IX.-15.X.2002”; 1♀ (NMNS), same but with “14.VII. –7.VIII.2007”; 1♂, 1♀ (TARI), Peitungyanshan (北東眼山), 16.IX.2013, leg. F.-S. Huang; 1♂ (TARI), Sungkang (松崗), 2.IV.1997, leg. W.-Y. Chou; 1♀ (TARI), same locality, 10.IV.2016, leg. Y.-T. Chung.

#### Redescription.

Length 4.7–5.2 mm, width 2.3–2.5 mm. Body color (Fig. 23A–C) reddish brown; vertex with one black spot at center; pronotum with three large black spots, one poorly defined, elongate spot at center, from basal 1/3 to apical 1/3, one pair laterally; scutellum black; five pairs of large black spots on elytra, one pair near base at middle, two pairs on the line at middle, one pair at apical 2/5 laterally, one pair at apical 1/5 near suture; metathoracic ventrites darker. Eyes small, interocular space 1.94–2.86 × diameter of eye. Antennae filiform in males (Fig. 24A), antennomere III apically broadened at apex, length ratios of antennomeres I–XI 1.0: 0.5: 1.0: 0.6: 0.5: 0.5: 0.5: 0.5: 0.4: 0.5: 0.7, length to width ratios of antennomeres I–XI 3.3: 2.2: 2.0: 2.1: 1.7: 1.9: 1.8: 1.8: 1.5: 1.6: 2.5; filiform in females (Fig. 24B), antennomere III not modified, length ratios of antennomeres I–XI 1.0: 0.6: 1.1: 0.6: 0.5: 0.5: 0.5: 0.5: 0.5: 0.5: 0.8, length to width ratios of antennomeres I–XI 3.2: 2.3: 4.5: 2.4: 1.9: 1.8: 1.7: 1.7: 1.7: 1.6: 2.6. Pronotum and elytra convex. Pronotum 1.9–2.1 × wider than long, disc with reticulate microsculpture; dense, extremely coarse punctures and extremely short pubescence; with median longitudinal and lateral depressions; lateral margins moderately rounded, apical margin slightly concave, basal margin straight; only posterior setiferous punctures erect. Elytra elongate and broad, parallel-sided, 1.5–1.6 × longer than wide; disc with reticulate microsculpture, and with dense, extremely coarse punctures and short pubescence. Apical spur of middle tibia small (Fig. 24E), tarsomere I basally narrowed in lateral view, with small tooth at middle ventrally in males (Fig. 24K). Aedeagus (Fig. 24C, D) slender in dorsal view, 5.5 × longer than wide, sides asymmetric, curved near apex, apex truncate; strongly curved at middle in lateral view; ostium not covered by membrane, ventrally located, located along lateral margin; two endophallic sclerites elongate, apex of primary endophallic sclerite acute, 0.6 × as long as aedeagus, secondary sclerite much shorter, 0.6 × as long as primary endophallic sclerite, apex acute. Only apices of gonocoxae (Fig. 24H) sclerotized, transverse, with two long setae at apex of each gonocoxa. Ventrite VIII (Fig. 24F) transverse; disc with several long setae and dense short setae along apical margin; spiculum long. Receptacle of spermatheca (Fig. 24F) very swollen; pump short and strongly curved; sclerotized proximal spermathecal duct wide and short. Apical margin of abdominal ventrite V truncate, with deeply rounded depression at middle in males (Fig. 24J); slightly concave in females (Fig. 24I).

#### Remarks.

Adults of *P. maculata* Gressitt and Kimoto and *P. tsoui* Bezděk and Lee may be separated from others within the species group by the five pairs of large black spots on the elytra (Fig. 23), the strongly curved aedeagus in lateral view (Figs 24C, 25C), and gonocoxa with only two setae (Figs 24H, 25J). adults of *P. maculata* differ from those of *P. tsoui* by the apically broadened antennomere III in males (Fig. 24A) (unmodified antennomere III, but IV with a large tubercle in those of *P. tsoui* (Fig. 25A)), and extremely slender antennomere III in females, > 4.0 × longer than wide (Fig. 24B); (slender antennomere III, < 4.0 × longer than wide in those of *P. tsoui* (Fig. 25B)). In males of *P. maculata*, the apex of the primary endophallic sclerite is acute, and lacks additional teeth on the secondary sclerite (Fig. 24C, D). The apex of the primary endophallic sclerite have several teeth and one additional tooth on the secondary sclerite in those of *P. tsoui* (Fig. 25C, D).

#### Food plants.

Possibly adults fed flowers of Lauraceae based on the following events. A specimen was collected by Mr Yi-Ting Chung 10 April 2016 in Sungkang by sweeping flowers of Lauraceae. Two specimens were collected by Mr Fu-Sheng Huang 16 September 2013 in Peitungyanshan by fogging Neolitsea aciculata (Bl.) Koidz. var. variabillima J.C. Liao (Lauraceae).

#### Distribution.

China, Taiwan.

### Pyrrhalta tsoui

Taxon classificationAnimaliaColeopteraChrysomelidae

Bezděk & Lee, 2019

1681EBF9-D68C-50A3-B656-E12F2FD44BAA

**Figure 26.**
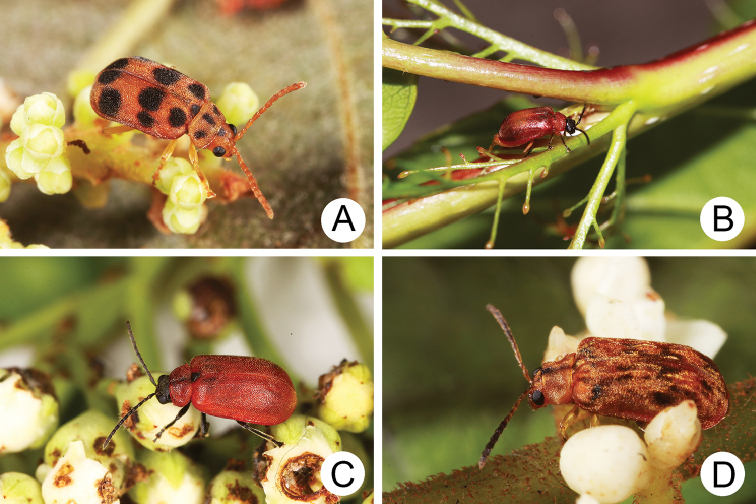
Field photographs of adults of *Pyrrhalta* species on host plants **A***P. tsoui* Bezděk & Lee **B***P. formosanensis* sp. nov. **C***P. semifulva* (Jacoby) **D***P. ishiharai* Kimoto.

Figs 23D–F , 25 , 26A

Pyrrhalta tsoui Bezděk & Lee, 2019: 531.

#### Other material

(specimens examined by Bezděk and Lee (2019) excluded). Taiwan. Nantou: 2♀ (KMNH), Lushan Wenchuan (廬山溫泉), 6.VI.1976, leg. H. Makihara (one identified as *P. maculata* by Kimoto, 1983); 1♂ (TARI), Tsuifeng (翠峰), 12–14.IX.1984, leg. K. S. Lin & S. C. Lin.

#### Redescription.

Length 4.6–5.3 mm, width 2.3–2.8 mm. Body color (Fig. 23D–F) reddish brown; vertex with one black spot at center; pronotum with three large black spots, one poorly defined elongate spot at center, from basal 1/3 to middle, one pair laterally; scutellum black; five pairs of large black spots on elytra, one pair near base at middle, two pairs in line with middle, one pair at apical 2/5 laterally, one pair at apical 1/5 near suture; metathoracic ventrites darker. Eyes small, interocular space 2.37–2.42 × diameter of eye. Antennae filiform in males (Fig. 25A), antennomere I swollen, IV with a large tubercle on outer sides at middle, length ratios of antennomeres I–XI 1.0: 0.5: 0.8: 0.9: 0.7: 0.7: 0.6: 0.6: 0.6: 0.6: 1.0, length to width ratios of antennomeres I–XI 2.1: 1.9: 2.7: 2.6: 2.1: 1.9: 1.6: 1.5: 1.5: 1.4: 2.4; filiform in females (Fig. 25B), antennomere I and IV not modified, length ratios of antennomeres I–XI 1.0: 0.6: 0.9: 0.8: 0.7: 0.6: 0.6: 0.6: 0.6: 0.6: 1.0, length to width ratios of antennomeres I–XI 2.4: 2.1: 3.4: 2.7: 2.2: 1.9: 1.7: 1.5: 1.6: 1.6: 2.5. Pronotum and elytra convex. Pronotum 1.9–2.0 × wider than long, disc with reticulate microsculpture; dense, extremely coarse punctures and extremely short pubescence; with median longitudinal and lateral depressions; lateral margins moderately rounded, apical and basal margins straight; anterior and posterior setiferous punctures erect. Elytra elongate and broad, parallel-sided, 1.4–1.6 × longer than wide; disc with reticulate microsculpture, and with dense extremely coarse punctures and short pubescence. Apical spur of tibia of middle leg small (Fig. 25E), tarsomere I not modified in males. Aedeagus (Fig. 25C, D) extremely slender in dorsal view, 8.4 × longer than wide, sides asymmetric, curved near apex, apex truncate; strongly curved at apical 1/3 and near base in lateral view, apex truncate; ostium not covered by membrane, ventrally located, along lateral margin; two endophallic sclerites elongate, apex of primary endophallic sclerite with several teeth, 0.6 × as long as aedeagus, secondary sclerite much shorter, 0.7 × as long as primary endophallic sclerite, apex acute, with one tooth near apex. Only apices of gonocoxae (Fig. 25J) sclerotized and transverse, with two long setae at apex of each gonocoxa. Ventrite VIII (Fig. 25F) transverse; disc with several long setae and dense short setae along apical margin; spiculum long. Receptacle of spermatheca (Fig. 25G) slightly swollen; pump short and strongly curved; sclerotized proximal spermathecal duct wide and short. Apical margin of abdominal ventrite V truncate, with deeply rounded depression at middle in males (Fig. 25I); slightly concave in females (Fig. 25H).

#### Remarks.

Adults of *P. tsoui* Bezděk & Lee and *P. maculata* Gressitt & Kimoto may be separated from others within the species group by the five pairs of large black spots on the elytra (Fig. 23), the strongly curved aedeagus in lateral view (Figs 24C, 25C), and gonocoxa with only two setae (Figs 24H, 25J). adults of *P. tsoui* differ from those of *P. maculata* by the normal antennomere III and antennomere IV with a large tubercle in males (Fig. 25A) (antennomere III apically broadened in *P. maculata* (Fig. 24A)), and slender antennomere III, < 4.0 × longer than wide in females (Fig. 25B) (extremely slender antennomere III, > 4.0 × longer than wide; in those of *P. maculata* (Fig. 24B)). In males of *P. tsoui*, the apex of the primary endophallic sclerite has several teeth and one additional tooth on the secondary sclerite (Fig. 25C, D). In *P. maculata* the primary endophallic sclerite is acute apically, and the secondary sclerite lacks additional teeth (Fig. 24C, D).

#### Food plant.

Adults feed on flowers of *Meliosma rhoifolia* Maxim. (Sabiaceae).

#### Distribution.

This species is widespread in lowlands of Taiwan.

### Pyrrhalta formosanensis sp. nov.

Taxon classificationAnimaliaColeopteraChrysomelidae

1A54B7F4-2057-5613-8DDE-0C1A74BC4B31

http://zoobank.org/8AB36966-903B-4853-8A90-7D6122D2DFFB

**Figure 27.**
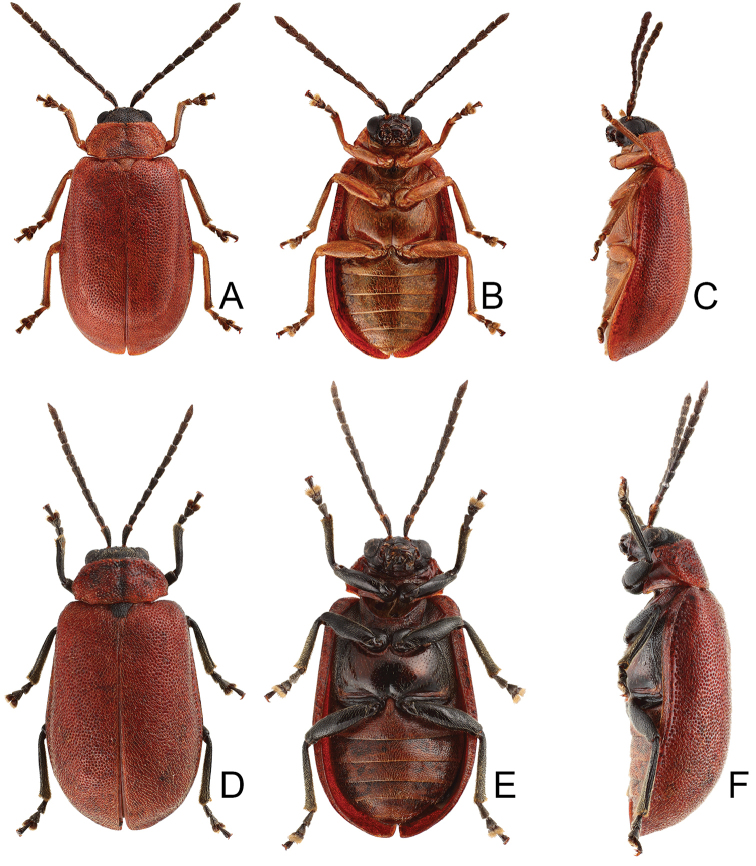
Habitus of *Pyrrhalta formosanensis* sp. nov. and *P. semifulva* (Jacoby) **A***P. formosanensis* sp. nov., female, dorsal view **B** ditto, ventral view **C** ditto, lateral view **D***P. semifulva*, from Taiwan, female, dorsal view **E** ditto, ventral view **F** ditto, lateral view.

**Figure 28.**
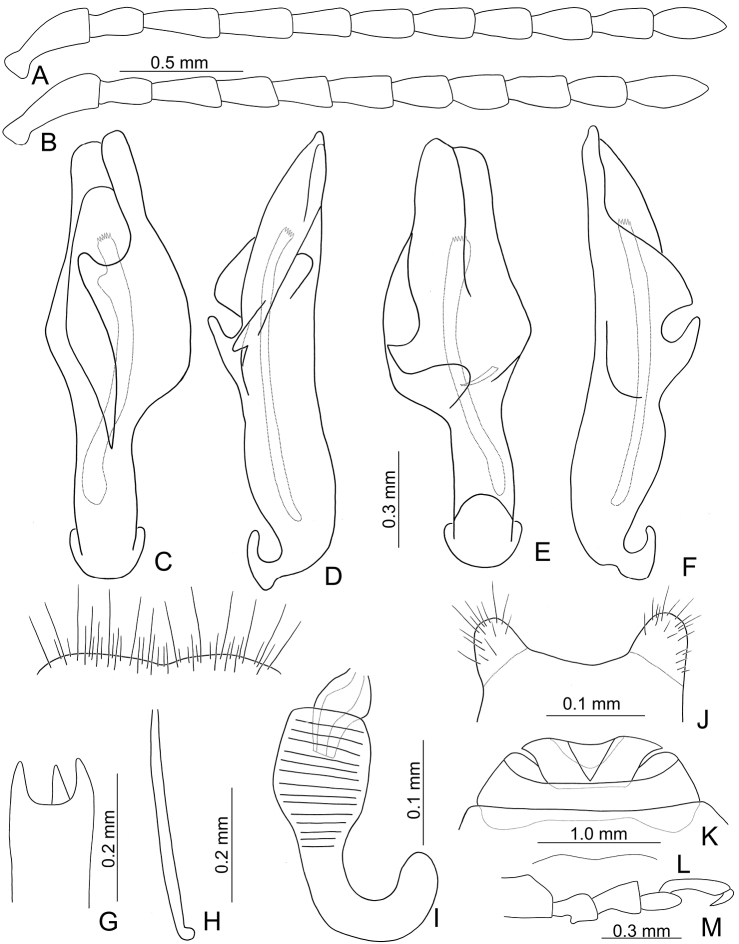
Diagnostic characters of *Pyrrhalta formosanensis* sp. nov. **A** antenna, male **B** antenna, female **C** aedeagus, dorsal view **D** ditto, left-side view **E** ditto, ventral view **F** ditto, right-side view **G** apex of tibia of middle leg, male **H** abdominal ventrite VIII **I** spermatheca **J** gonocoxae **K** abdominal ventrite V, male **L** abdominal ventrite V, female **M** tarsi of middle leg, male.

Figs 26B , 27A–C , 28

#### Types.

***Holotype*** ♂ (TARI), Taiwan. Kaohsiung, Tienchih (天池), 2.VII.2009, leg. M.-H. Tsou. ***Paratypes*.** 1♂, 12♀, same data as holotype; Hualien: 1♂ (TARI), Kuanyuan (關原), 2.VII.2008, leg. M.-H. Tsou; 1♂ (TARI), Pilu (碧綠), 6.VII.2018, leg. H.-F. Lu; Ilan: 1♀ (TARI), Chienching trail (見晴步道), 23.IV.2019, leg. M.-D. Chen; 1♀ (TARI), Tsuifenghu (翠峰湖), 15.VIII.2007, leg. S.-S. Li; Kaohsiung: 1♂ (TARI), Chungchihkuan (中之關), 10.VI.2015, leg. C.-F. Lee; Nantou: 1♂ (TARI), Meifeng (梅峰), 24–26.VI.1981, leg. K. S. Lin & W. S. Tang; 1♂ (TARI), Piluhsi (碧綠溪), 8.VII.2015, leg. C.-F. Lee; 1♂ (TARI), Tsuifeng (翠峰), 30.VII.2014, leg. C.-F. Lee.

#### Diagnosis.

Legs reddish brown; tibia of middle leg with apical spine; tarsomere I modified. Sides of ventrite V strongly shortened in males.

#### Description.

Length 4.6–5.5 mm, width 2.4–2.9 mm. Body color (Fig. 27A–C) reddish brown; head black but antennae dark brown. Eyes small, interocular space 2.62–2.69 × diameter of eye. Antennae filiform in males (Fig. 28A), length ratios of antennomeres I–XI 1.0: 0.6: 0.8: 0.8: 0.6: 0.7: 0.6: 0.6: 0.6: 0.6: 0.8, length to width ratios of antennomeres I–XI 2.8: 1.9: 2.5: 2.4: 2.0: 2.2: 2.0: 1.9: 1.9: 2.0: 2.4; similar in females (Fig. 28B), length ratios of antennomeres I–XI 1.0: 0.5: 0.7: 0.6: 0.6: 0.6: 0.6: 0.6: 0.6: 0.5: 0.7, length to width ratios of antennomeres I–XI 3.2: 2.0: 2.4: 2.2: 1.9: 2.0: 1.9: 1.7: 1.7: 1.6: 2.2. Pronotum and elytra convex. Pronotum 2.1–2.2 × wider than long, disc with dense, extremely coarse punctures and extremely short pubescence, with transverse ridge near apical margin deflexed near antero-lateral corners, with median longitudinal and lateral depressions; lateral margins moderately rounded, apical margin slightly concave, basal margin straight; anterior and posterior setiferous punctures slightly erect. Elytra elongate and broad, parallel-sided, 1.4–1.5 × longer than wide; disc with dense, extremely coarse punctures and extremely short pubescence. Apical spur of tibia of middle leg slender (Fig. 28G), tarsomere I axe-shaped in lateral view, with narrow basal part and expanded apical 2/3, posterior angles of expanded part narrowly rounded (Fig. 28M) in males. Aedeagus (Fig. 28C–E) extremely asymmetric in dorsal view, inner margin of right side expending at apical 1/3, covering right side of ostium, lateral margin of right side expanding downwards, with a notch at middle; inner margin of left side expanding inwards at basal 2/5 and apical 2/5; primary endophallic sclerite elongate, several fine teeth on apex. Sclerotized gonocoxae (Fig. 28J) transverse, both gonocoxae separated, with several long setae near apices. Ventrite VIII (Fig. 28H) transverse; disc with dense, long setae along apical margin; spiculum short. Receptacle of spermatheca (Fig. 28I) slightly swollen; pump short and strongly curved; sclerotized proximal spermathecal duct wide and short. Apical margin of abdominal ventrite V slightly concave medially, with deep triangular depression at middle in males, sides of abdominal ventrite V shortened, sides of basal margin of abdominal ventrite IV expanding downwards in males (Fig. 28K); only slightly concave in females (Fig. 28L).

#### Remarks.

Adults of *P. formosanensis* sp. nov. are similar to those in Taiwanese populations of *P. semifulva* with their reddish bodies, but differ in the reddish brown scutellum, legs, and thoracic ventrites (Fig. 27B) (black scutellum, legs, and thoracic ventrites (Fig. 27E) in *P. semifulva*), modified sides of abdominal ventrite V (Fig. 28K), and mesotarsomere I of middle leg (Fig. 28M) in males, and very characteristic aedeagus (Fig. 28C–E).

#### Food plant.

Adults feed on flowers of *Prunus campanulata* Maxim. (Rosaceae) (Fig. 26B).

#### Distribution.

The species is widespread at mid-altitudes (1,500–2,500 m) in Taiwan.

#### Etymology.

This species is named after Taiwan, a beautiful island.

### Pyrrhalta semifulva

Taxon classificationAnimaliaColeopteraChrysomelidae

(Jacoby, 1885)

C81A269E-DA7D-58E8-8E64-32C578A9AEE6

**Figure 29.**
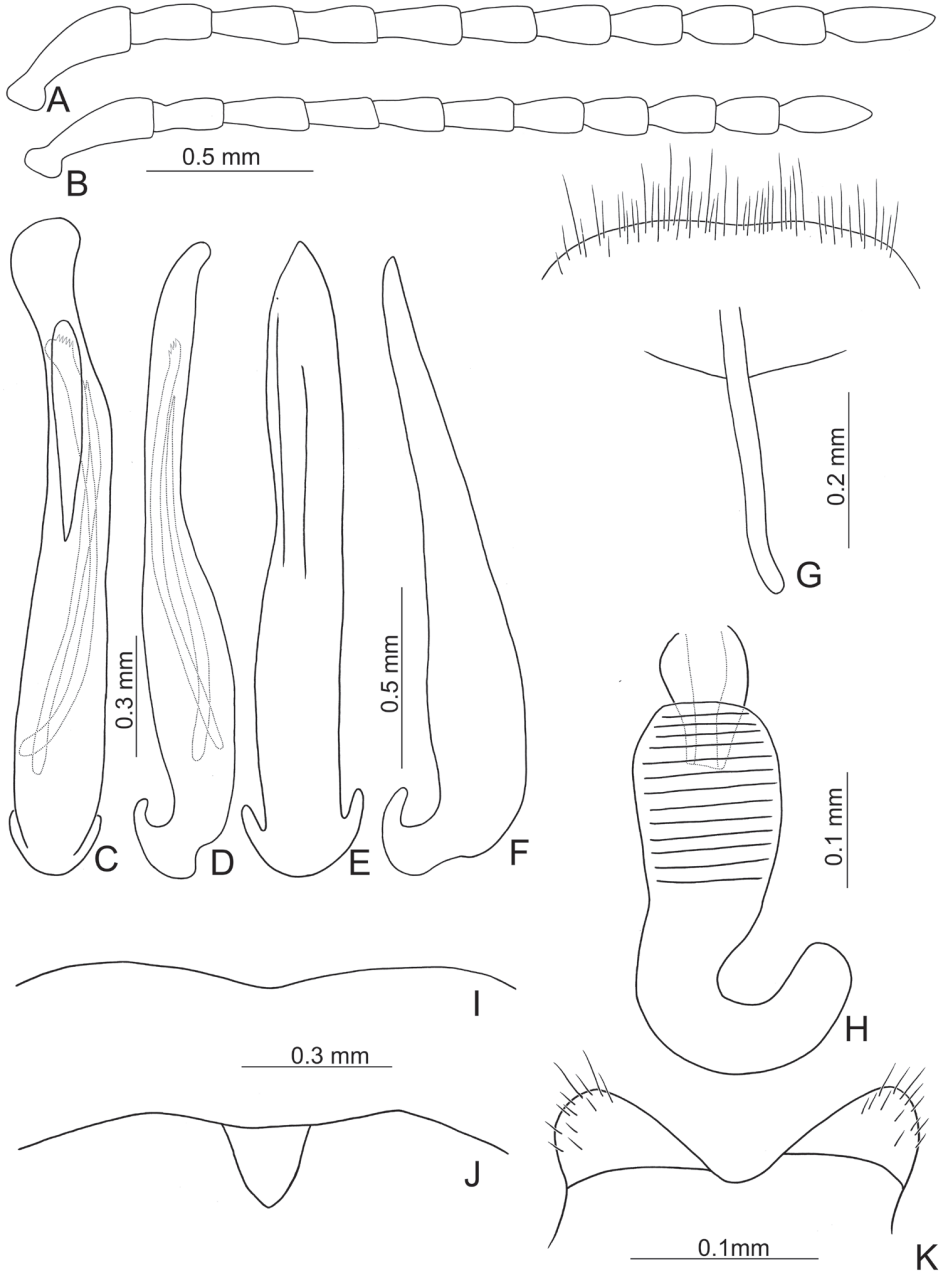
Diagnostic characters of *Pyrrhalta semifulva* (Jacoby) **A** antenna, male **B** antenna, female **C** aedeagus, typical form, dorsal view **D** ditto, lateral view **E** aedeagus, variation (endophallic sclerites omitted), dorsal view apex of tibia of middle leg, male **F** ditto, lateral view **G** abdominal ventrite VIII **H** ermatheca **I** abdominal ventrite V, female **J** abdominal ventrite V, male **K** gonocoxae.

**Figure 30.**
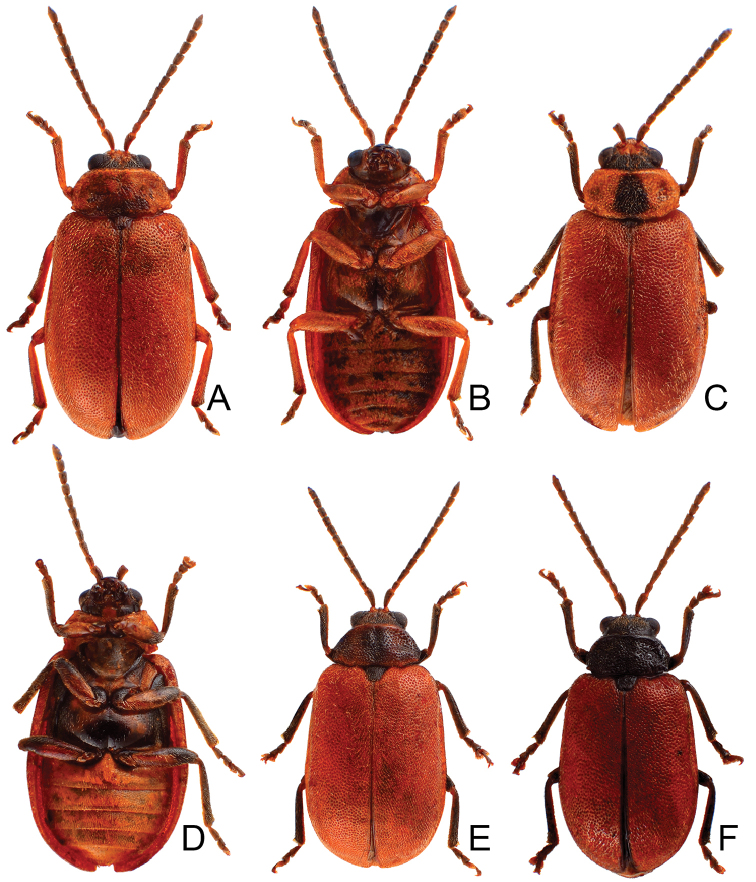
Habitus of *Pyrrhalta semifulva* (Jacoby) from Japan **A** female, color variation, dorsal view **B** ditto, ventral view **C** Female, color variation, dorsal view **D** ditto, ventral view **E** female, color variation, dorsal view **F** male, color variation, dorsal view.

Figs 26C , 27D–F , 29 , 30

Gallerucella semifulva Jacoby, 1885: 745 (Japan: Kiga).
Lochmaea (Tricholochmaea) semifulva : Laboissière 1932: 964; Ogloblin 1936: 91 (redescription); Chûjô 1940: 112 (Japan: Kyushu, Shikoku); Chûjô 1954: 57 (Japan: Shikoku); Wilcox 1971: 82 (catalogue).
Tricholochmaea semifulva : Chûjô & Kimoto 1961: 169 (catalogue); Kimoto 1964b: 373 (deposition of type specimens); Lopatin et al. 2004: 130 (catalogue); Beenen 2010: 455 (catalogue).
Pyrrhalta semifulva : Kimoto 1964a: 299; Kimoto and Hiura 1971: 15 (Japan); Kimoto and Gressitt 1966: 476 (key), 520 (Ryukyus); Kimoto 1974: 24 (Taiwan); Nakane 1984: 626 (Japan); Kimoto 1985: 4 (catalogue); Kimoto 1986: 56 (additional records in Taiwan); Kimoto 1987: 188 (additional records in Taiwan); Medvedev and Roginskaya 1988: 116 (host plants); Kimoto 1989a: 268 (additional records in Taiwan); Kimoto 1991: 9 (additional records in Taiwan); Li 1992: 184 (China: Liaoning); Kimoto and Takizawa 1994: 234 (key), 307 (Japan); Kimoto and Chu 1996: 57 (catalogue); Kimoto and Takizawa 1997: 300 (key), 374; Wang and Yang 1998: 68 (China: Fujian); Yang 2002: 628 (China: Fujian); Xue and Yang 2010: 129 (catalogue); Yang et al. 2015: 120 (catalogue); Matsumura et al. 2017: 85 (female reproductive system).
Pyrrhalta (Tricholochmaea) semifulva : Wilcox 1965: 38; Medvedev 1992: 580 (key); Medvedev and Dubeshko 1992: 133 (key); Medvedev 2006: 141 (Russia: Far East).
Gallerucella modesta Jacoby, 1885: 745 (Japan: Nikko). Synonymized by Chûjô 1954: 57.
Lochmaea (Tricholochmaea) modesta : Chûjô 1940: 112 (Japan: Shikoku).
Gallerucella signaticeps Weise, 1887: 191 (Vladivostok). Synonymized by Ogloblin 1936: 91.
Lochmaea japonica Weise, 1922: 67 (Japan: Honshu). Synonymized by Laboissière 1932: 964.

#### Types.

*Gallerucella semifulva*. ***Lectotype*** ♀ (NHMUK, here designated): “Kiga [h, w, underside of card glued with specimen] // Type / H.T. [p, w, circle label with red border] // Japan. / G. Lewis. / 1910-320. [p, w] // *Galeruca* / *semifulva* Jac [h, b] // SYN- / TYPE [p, w, circle label with blue border]”. Paralectotypes. 1♂ (NHMUK): “Kiga [h, w, underside of card glued with specimen] // Japan / Lewis [h, w] // Jacoby Coll. / 1909-28a. [p, w] // semifulva Jac [h, b]”; 1♂ (NHMUK): “Kiga [h, w, underside of card glued with specimen] // Japan. / G. Lewis. / 1910-320. [p, w]”; 1♀ (NHMUK): “Japan. / G. Lewis. / 1910-320. [p, w]”; 1 (TARI, sex undetermined): “KIGA [h] / JAPAN [p] / 18.III.1880 [h] / Col. G. LEWIS [p, w] // CO / Type [p, circle label with yellow letters border] // *Gallerucella* / *semifulva* / Jacoby [h] / DET. M. CHUJO [p, w] // *Gallerucella* / *semifulva* Jac. [h] / Det. T. Shiraki [p, w] // 1934 [p, w]”; 1 (MCZC, sex undetermined): “Japan / Lewis [h, w] // 1st Jacoby / Coll. [p, w] // Type. / Sp. figured. [p, w] // Type [p] / 17878 [h, r]”. Since types much be collected from “Kiga” based on Jacoby (1885). Those specimens with different localities but with type labels should not be regarded as type series, including 1 (NHMUK, sex undetermined): “marshes / nagasaki (h, w) // Japan. / G. Lewis. / 1910-320. [p, w] // SYN- / TYPE [p, w, circle label with blue border]”; 1♂ (NHMUK): “Subashiri [h, w, underside of card glued with specimen] // Japan. / G. Lewis. / 1910-320. [p, w] // SYN- / TYPE [p, w, circle label with blue border]”; 1 (TARI, sex undetermined): “Ipongi [h] / JAPAN [p] / III.1881 [h] / Col. G. LEWIS [p] // CO / Type [p, circle label with yellow letters border] // *Galerucella* / *semifulva* / Jacoby [h] / DET. M. CHUJO [p, w]”.

*Gallerucella modesta*. Lectotype (sex undetermined, NHMUK, here designated): “Nikko [h, w, underside of card glued with specimen] // Type / H.T. [p, w, circle label with red border] // Japan. / G. Lewis. / 1910-320. [p, w] // Nikko. [p, w] // *Galerucella* / *modesta* Jac. [h, b]”. Paralectotypes. 1 (NHMUK, sex undetermined): “Nikko. [p, w] // Japan. / G. Lewis. / 1910-320. [p, w] // *Galerucella* / *modesta* / Jac [h, w] // *Lochmaea* / (*Tricholochmaea*) *semifulva* Jacob. [h] / D. Ogloblin det. [p, w]”; 1 (MCZC, sex undetermined): “Japan / Lewis [h, w] / 1st Jacoby / Coll. [p, w] // modesta Jac. [h, b] // Type [p] / 17877 [h, r]”.

#### Other material.

**Japan.** Hokkaido: 1♀ (HSC), Etetsu-shi, Nopporo, 18.VI.2011, leg. H. Suenaga; 1♀ (HSC), Tomakomai-shi, Lake Utonai-ko, 29.VIII.2011, leg. H. Suenaga; 1♂ (HSC), same but with “22.V.2012”; Honshu: 1♂, 1♀ (HSC), Akita Pref., Nikaho-shi, Chôkaisan, Hokodate, 10.VI.2016, leg. S. Sejima; 1♀ (TARI), Aomori Pref., 29.VI. 1934, leg. F. Watanabe; 1♀ (TARI), Aomori Pref., Hatinohe, 1.VI.1933, leg. A. Fukuda; 1♀ (KMNH), Fukui Pref., Mt. Monju, 3.V.1963, leg. H. Sasaji; 1♀ (NMNS), Gifu Pref., 2.IV.1946; 1♂ (NMNS), same locality, 6.V.1947, leg. K. Ohbayashi; 1♂ (NMNS), Gifu Pref., Suhara, 15.IV.1956, leg. K. & M. Ohbayashi; 1♀ (NMNS), same locality, 5.V.1955, leg. K. Ohbayashi; 1♂ (NMNS), same but with “13.V.1956”; 1♂, 1♀ (NMNS), same but with “15.V.1956”; 1♂ (NMNS), same locality, 3.V.1957, leg. N. Ohbayashi; 1♂ (NMNS), same but with “19.V.1957”; 1♂ (NMNS), same but with “1♂ (NMNS), Gifu Pref., Tanigumi, 11.VI.1941, leg. K. Ohbayashi; 1♀ (HSC), Gunma Pref., Minakami-machi, Fujiwara, 6.VI.2008, leg. S. Sejima; 1♀ (HSC), Hiroshima Pref., Akioota-cho, Mt. Mushiki-toge, 14.VI.2010, leg. H. Suenaga; 2♀ (HSC), Hiroshima Pref., Takano-cho, Takano, 13.VI.2009, leg. H. Suenaga; 8♂, 5♀ (NMNS), Hyogo Pref. Mt. Oginosen, 4.V.1964, leg. M.-H. Chûjô; 8♂, 4♀ (TARI), same locality, 2.V.1965, leg. Y. Ohira; 1♂ (HSC), Kanagawa Pref., Zushi-shi, Junmu-ji, 18.IV.2012, H. Suenaga; 1♀ (TARI), Nagano Pref., Koganezawa, 12.V.1935, leg. S. Miyamoto; 1♂, 1♀ (KMNH), Nagano Pref., Misuzuko, 27.VII.1973, leg. S. Kimoto; 1♂ (KMNH), Nagano Pref., Wada, 10.VII.1951, leg. M. Takahashi; 1♂ (TARI), Nagano Pref., Yarisawa, 25.VII.1931, leg. K. Obayashi; 1♂ (TARI), Niigata Pref., Sado, Suizu, 22.V.1937, leg. K. Baba; 1♂, 2♀ (HSC), Okayama Pref., Tomata-Gun, Kagamino-cho, 4.V.2006, leg. H. Suenaga; 1♀ (HSC), same but with “Shiraka-keikoku”;1♀ (HSC), Okayama Pref., Niimi-shi, Toyanagakôma, 2.VII.2006, leg. S. Sejima; 1♂ (TARI), Tochigi Pref., Nikko, Sanno-Toge, 16.VI.1940, leg. Tn. Nakane; Kyushu: 2♂ (TARI), Fukuoka Pref., Mt. Hiko, 7.VII.1934, leg. K. Yamauchi; 1♀ (TARI), same locality, 14.VII.1941, leg. M. Chujo; 1♀ (TARI), Kagoshima Pref., Kirishima, 16.VII.1939, leg. Y. Takemura; 1♂, 1♀ (HSC), Kagoshima Pref., Minamioosumi-machi, Sata-misaki, 6.V.2013, leg. H. Suenaga; 1♀ (HSC), Oita Pref., Kamizue-cho, Hakuso, 5.V.2013, leg. H. Suenaga; 1♀ (HSC), Oita Pref., Yufu-shi, Kurodake, 11.VI.2006, leg. S. Sejima; Shikoku: 1♀ (HSC), Ehime Pref., Kumakogen-cho, Mt. Ishizuchi, Tsuchigoya, 28.VI.2009, leg. H. Suenaga; 1♂ (HSC), Ehime Pref., Kumakogen-cho, Mt. Saraga-mine, 23.V.2009, leg. H. Suenaga; 1♂ (HSC), Ehime Pref., Kumakogen-cho, Omogokei, 16.VII.2007, leg. H. Suenaga; 1♀ (HSC), Ehime Pref., Matsuyama-shi, Komenono, 27.V.2007, leg. T. Ichiyanagi; 1♀ (HSC), same locality, 26.V.2007, leg. H. Suenaga; 3♀♀ (HSC), Ehime Pref., Matsuyama-shi, Shukuno, near the dame of Ishitegawa, 2.V.2010, leg. K. Hashimoto; 1♀ (HSC), Ehime Pref., Uwajima-shi, Onigajôzan to Yatsurayama, 7.V.2007, leg. S. Sejima; 1♂, 1♀ (HSC), Kagawa Pref., Mannou-cho, Nakadouri, Mt. Daisenzan, 29.VII.2007, leg. H. Suenaga; 2♀♀ (TARI), Kooti-Ken (= Kochi Pref.), leg. I. Okubo; 1♀ (TARI), same but with “26.V.1935”; 1♂, 1♀ (TARI), same but with “24.VII.1936”; 1♀ (HSC), Tokushima Pref., Yoshinokawa-shi, Mt. Kotsu-zan, 18.V.1987, leg. S. Mano; Taiwan. Chiayi: 1♀ (KMNH), Alishan (阿里山), 6.VII.1965, leg. S. Kimoto, det. S. Kimoto, 1974; Hsinchu: 1♀ (TARI), Lupi (魯壁), 10.VII.2010, leg. M.-H. Tsou; Hualien: 1♀ (TARI), Hahuan Cross-Ridge (合歡越嶺古道), 4.VIII.2018, leg. H.-F. Lu; Ilan: 1♀ (TARI), Ssuyuan (思源), 28.IV.2009, leg. M.-H. Tsou; Kaohsiung: 1♀ (KMNH), Liukuei (六龜), V.1985, leg. W. L. Chen, Nagoya Univ. Col., det. S. Kimoto, 1987; Nantou: 1♂ (NMNS), Shanlinchi (杉林溪), 11.V.1990, leg. C. C. Chiang; 1♀ (TARI), Sungkang (松崗), 18.IV.2015, leg. B.-X. Guo; 1♀ (TARI), Tsuifeng (翠峰), 12–14.IX.1984, leg. K. S. Lin and S. C. Lin; Taoyuan: 1♀ (KMNH), Lalashan (拉拉山), 7.V.1982, leg. N. Ohbayashi.

#### Redescription.

Length 4.3–5.4 mm, width 2.4–3.0 mm. Body color (Fig. 27D–F) reddish brown; head (including antennae), scutellum, thoracic ventrites, and legs black. Eyes small, interocular space 2.50–2.86 × diameter of eye. Antennae filiform in males (Fig. 29A), length ratios of antennomeres I–XI 1.0: 0.6: 0.6: 0.6: 0.6: 0.6: 0.5: 0.5: 0.5: 0.5: 0.8, length to width ratios of antennomeres I–XI 3.1: 2.6: 2.2: 2.1: 2.3: 2.1: 2.0: 2.0: 1.9: 1.9: 3.2; similar in females (Fig. 29B), length ratios of antennomeres I–XI 1.0: 0.5: 0.6: 0.5: 0.5: 0.6: 0.5: 0.5: 0.5: 0.5: 0.7, length to width ratios of antennomeres I–XI 3.1: 1.9: 2.2: 1.9: 1.8: 2.0: 1.8: 1.8: 1.9: 1.8: 2.5. Pronotum and elytra convex. Pronotum 2.2–2.3 × wider than long, disc with reticulate microsculpture; with dense, extremely coarse punctures and extremely short pubescence, with transverse ridge near apical margin that curves downwards near antero-lateral corners, no punctures or pubescence above ridge but coarse punctures present on antero-lateral corners; with median longitudinal and lateral depressions; lateral margins moderately rounded, apical margin slightly concave, basal margin straight; anterior and posterior setiferous punctures slightly erect. Elytra elongate and broad, parallel-sided, 1.4–1.5 × longer than wide; disc smooth, with extremely coarse, dense punctures and extremely short pubescence. Apical spur of tibia of middle leg absent, tarsomere I not modified in males. Aedeagus (Fig. 29C, D) extremely slender in dorsal view, 7.9 × longer than wide, sides asymmetric, parallel-sided, apex truncate, curved near apex; strongly curved near base in lateral view, apex narrowly rounded; ostium not covered membrane; two endophallic sclerites elongate, several fine teeth on apex of primary endophallic sclerite, 0.6 × as long as aedeagus, secondary sclerite a little shorter, 0.9 × as long as primary endophallic sclerite, apex acute. Sclerotized gonocoxae (Fig. 29K) stout and cylindrical, gonocoxae separated, disk with several longer setae mixed with dense, shorter setae. Ventrite VIII (Fig. 29G) transverse; disc with three layers of different lengths of setae on apical area, shortest setae along apical margin, longest setae a slightly before apex, intermediate setae further from apex; spiculum short. Receptacle of spermatheca (Fig. 29H) slightly swollen; pump short and strongly curved; sclerotized proximal spermathecal duct wide and short. Apical margin of abdominal ventrite V slightly concave medially, with deep depression at middle in males (Fig. 29J); only slightly concave in females (Fig. 29I).

#### Variation.

Aedeagi of many individuals have apically tapering apices and look straight in lateral view (Fig. 29E, F). Japanese populations display great color variation. Some individuals (Hokkaido) have the entire reddish brown bodies but one black spot is present on the vertex, and five apical antennomeres are darkened. Some are similar to the previous ones, but the head is black except mouth parts (Fig. 30A, B). Some are similar the previous ones, but the pronotum has one black spot at center, without a clear margin; legs are reddish brown but outer sides of tibiae and entire tarsi are dark brown, antennae and the scutellum are blackish brown (Fig. 30C, D). Some specimens are similar to Taiwanese populations but with different degrees of darkness on the pronotum (Fig. 30E, F).

#### Remarks.

*Pyrrhalta semifulva* (Jacoby) and *P. formosanensis* sp. nov. may be separated from others within the species group by the reddish brown bodies (Figs 27, 30) and short, cylindrical gonocoxae with dense setae (Figs 28J, 29K). Taiwanese populations of *P. semifulva* differ from *P. formosanensis* sp. nov. by the black scutellum, legs, and thoracic ventrites (Fig. 27E) (reddish brown scutellum, legs, and thoracic ventrites (Fig. 27B) in *P. formosanensis* sp. nov.), normal abdominal ventrite V (Fig. 29I), and unmodified mesotarsomere I of middle leg in males (modified abdominal ventrite V (Fig. 28K) and mesotarsomere of middle leg (Fig. 28M in males of *P. formosanensis* sp. nov.). In males of *P. semifulva*, the elongate and apically curved aedeagus is similar to that of *P. discalis* Gressitt and Kimoto, but differs in the relatively longer secondary endophallic sclerite, 0.9x as long as primary endophallic sclerite (Fig. 29C, D) (vs. relatively shorter secondary endophallic sclerite, 0.6x as long as primary endophallic sclerite in *P. discalis* (Fig. 32C, D).

#### Food plants.

Rosaceae: *Prunus jamasakura* Sieb., ex Koidz., *P. yedoensis* Matsum., and *Sorbus japonica* (Decne.) Hedl.; Hamamelidaceae: *Corylopsis gotoana* Makino, (Chûjô and Kimoto 1961). adults of Taiwanese populations feed on flowers of *Photinia niitakayamensis* Hayata (Rosaceae) (Fig. 26C).

#### Distribution.

Japan, Russian, Taiwan. The species is widespread at mid-altitudes (1,500–2,500 m) in Taiwan.

### Pyrrhalta discalis

Taxon classificationAnimaliaColeopteraChrysomelidae

Gressitt & Kimoto, 1963

9940893C-FFDB-5E0A-BDA4-E2CCDF8E2029

Figs 31 , 32 , 33

Pyrrhalta discalis Gressitt & Kimoto, 1963: 448 (China: Hubei); Kimoto 1974: 24 (Taiwan); Kimoto and Chu 1996: 55 (catalogue); Kimoto and Takizawa 1997: 300 (key), 373; Beenen 2010: 452 (catalogue); Xue and Yang 2010: 122 (catalogue); Yang et al. 2015: 116 (catalogue).
Pyrrhalta (Pyrrhalta) discalis : Wilcox 1971: 85 (catalogue).

#### Types.

***Holotype*** ♂ (CAS, by original designation): “Suisapa, 1000 M. / Lichuan Distri. / W. Hupeh, China / VII- [p] 25 [h] -48 [p, w] // Ridge above / 1200-1500 M [p, w] // J. L. Gressitt / Collector [p, w] // Pyrrhalta / discalis / G&K [h] / J. L. Gressitt det. // HOLOTYPE [p] / Pyrrhalta / discalis [h] / Gressitt & Kimoto [p, r]. ***Paratypes*.** 1♀ (CAS): “Suisapa, 1000 M. / Lichuan Distri. / W. Hupeh, China / VII- [p] 23 [h] -48 [p, w] // J. L. Gressitt / Collector [p, w] // ALLOTYPE [p] / Pyrrhalta / discalis / S. Kimoto & [h] / J. L. Gressitt [p, r] // Pyrrhalta / discalis / Gress & Kim. [h] / Gressitt & Kimoto det. 196[p]2 [h, w]”; 1♂ (CAS): “W. HUPEH / China, Suisapa, / Lichuan, 1000 m. / IX-[p] 17 [h] 1948 [p, w] // Gressitt & / Djou Collrs. [p, w] // PARATYPE [p] / Pyrrhalta / discalis [h] / Gressitt & Kimoto [p, y]”; 1♀ (CAS): “Suisapa, 1000 M. / Lichuan Distri. / W. Hupeh, China / VII- [p] 23 [h] -48 [p, w] // J. L. Gressitt / Collector [p, w] // PARATYPE [p] / Pyrrhalta / discalis [h] / Gressitt & Kimoto [p, y]”; 1♀ (CAS): “Suisapa, 1000 M. / Lichuan Distri. / W. Hupeh, China / VII- [p] 24 [h] -48 [p, w] // J. L. Gressitt / Collector [p, w] // PARATYPE [p] / Pyrrhalta / discalis [h] / Gressitt & Kimoto [p, y]”; 1♀ (BPBM): “Suisapa, 1000 M. / Lichuan Distri. / W. Hupeh, China / VII- [p] 22 [h] -48 [p, w] // Gressitt & / Djou Collrs. [p, w] // PARATYPE [p, b] // Pyrrhalta / discalis / Paratype G&K [h] / J.L. Gressitt det. [p, w].

#### Other material.

Taiwan. Hsinchu: 1♂ (TARI), Litungshan (李棟山), 15.III.2009, leg. S.-F. Yu; 1♀ (TARI), Lupi (魯壁), 25.II.2010, leg. S.-F. Yu; 1♀ (TARI), Wuchihshan (五指山), 27.III.2008, leg. H. Lee; 1♂ (TARI), same locality, 14.V.2008, leg. S.-F. Yu; Hualien: 1♀ (TARI), Pulowan (布洛灣), 26.III.2016, leg. H.-F. Lu; 1♂, 1♀ (TARI), same but with “31.III.2016”; 10♂, 4♀ (TARI), same but with “30.IV.2016”; 1♀ (TARI), same but with “9.V.2016”; Pingtung: 1♂ (TARI), Tahanshan (大漢山), 29.VI.2018, leg. Y.-T. Chung; Taichung: 1♀ (KMNH), Pahsienshan (八仙山), 29.V.1971, leg. K. Kanmiya, det. S. Kimoto, 1973; Taipei: 1♂ (TARI), Chihshanyan (芝山岩), 2.V.2016, leg. M.-H. Tsou; 1♂ (TARI), Wulai (烏來), 17.V.2008, leg. M.-H. Tsou; Taoyuan: 1♀ (TARI), Lalashan (拉拉山), 2.IV.2009, leg. H. Lee; 1♀ (TARI), Nantzukou (湳仔溝), 24.IV.2016, leg. M.-H. Tsou; 1♀ (TARI), Yongfu (永福), 17.IV.2011, leg. M.-H. Tsou; 1♂, 1♀ (TARI), same but with “30.IV.2011”; 2♂, 1♀ (TARI), same but with “11.V.2011”; 1♂ (TARI), same but with “20.IV.2015”.

#### Redescription.

**Figure 31.**
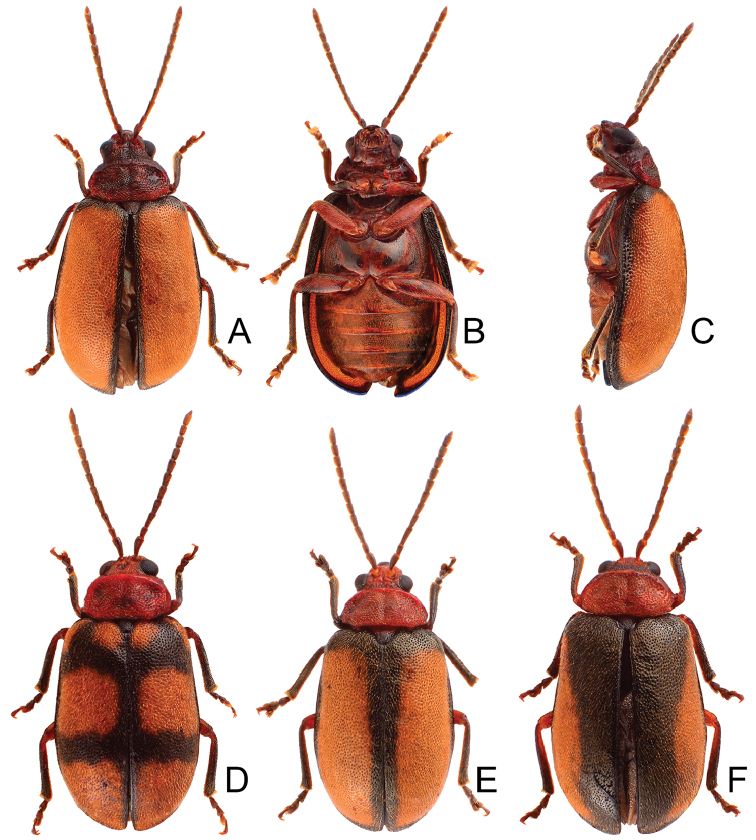
Habitus of *Pyrrhalta discalis* Gressitt & Kimoto **A** male, typical form, dorsal view **B** ditto, ventral view **C** ditto, lateral view **D** male, color variation, dorsal view **E** male, color variation, dorsal view **F** male, color variation, dorsal view.

**Figure 32.**
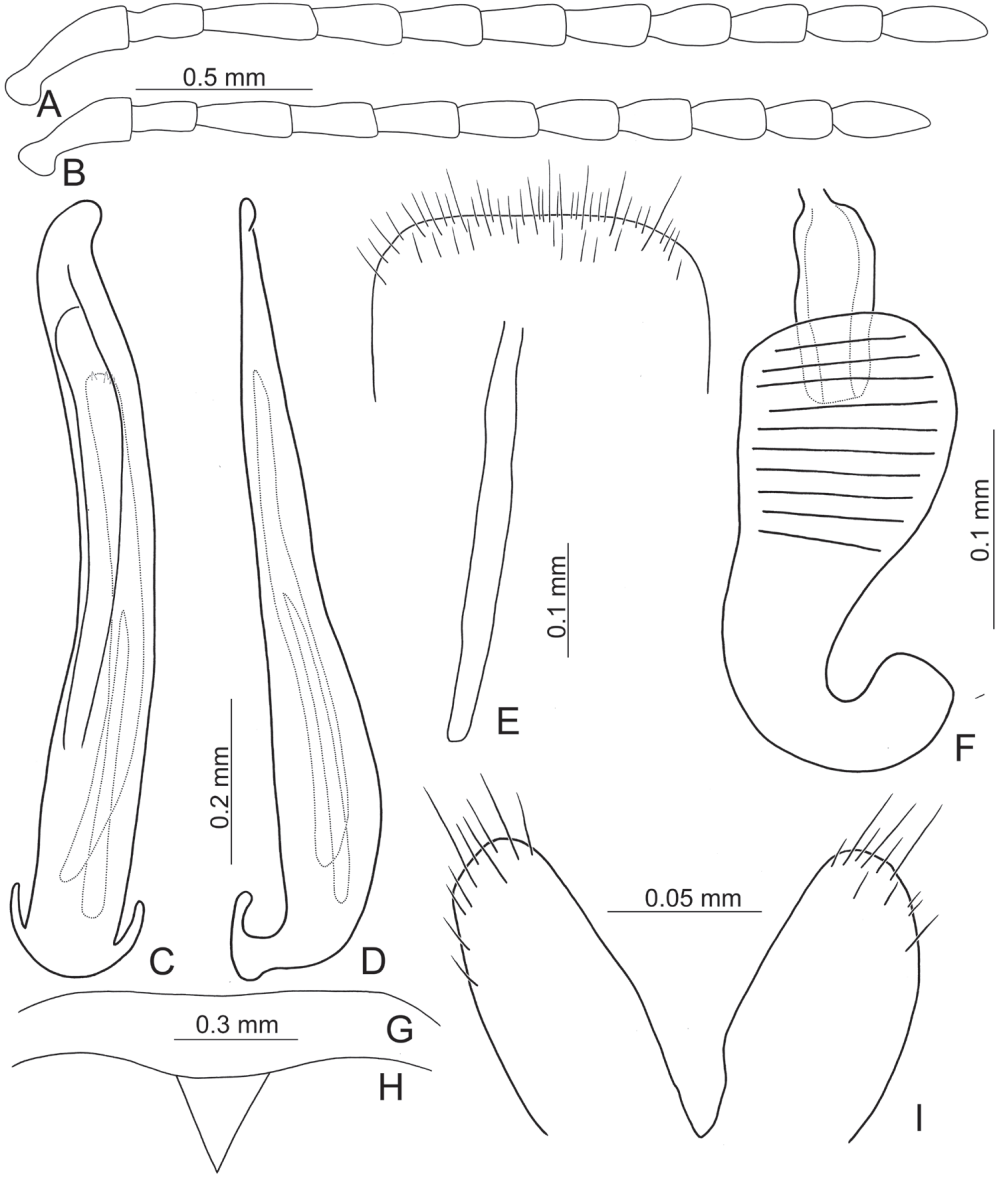
Diagnostic characters of *Pyrrhalta discalis* Gressitt & Kimoto **A** antenna, male **B** antenna, female **C** aedeagus, dorsal view **D** ditto, lateral view **E** abdominal ventrite VIII **F** spermatheca **G** abdominal ventrite V, female **H** abdominal ventrite V, male **I** gonocoxae.

Length 4.6–5.6 mm, width 2.3–2.8 mm. Body color (Fig. 31A–C) yellowish brown; head and prothorax reddish brown, but antennae blackish brown; with wide black stripes along lateral margins and suture of elytra; tibiae and tarsi black. Eyes small, interocular space 2.09–2.49 × diameter of eye. Antennae filiform in males (Fig. 32A), length ratios of antennomeres I–XI 1.0: 0.5: 0.8: 0.6: 0.6: 0.6: 0.6: 0.6: 0.5: 0.5: 0.7, length to width ratios of antennomeres I–XI 3.4: 2.3: 3.2: 2.2: 2.1: 2.0: 2.0: 2.0: 2.0: 2.0: 2.9; similar in females (Fig. 4B), length ratios of antennomeres I–XI 1.0: 0.5: 0.8: 0.7: 0.7: 0.7: 0.7: 0.6: 0.6: 0.5: 0.8, length to width ratios of antennomeres I–XI 2.7: 2.2: 2.7: 2.4: 2.3: 2.2: 2.0: 1.8: 1.9: 1.7: 2.6. Pronotum and elytra convex. Pronotum 2.0–2.1 × wider than long, disc with dense, extremely coarse punctures and extremely short pubescence, with median longitudinal and lateral depressions; lateral margins moderately rounded, apical margin slightly concave, basal margin straight; anterior and posterior setiferous punctures slightly erect. Elytra elongate and broad, parallel-sided, 1.4 × longer than wide; disc with dense extremely coarse punctures and extremely short pubescence. Apical spur of tibia of middle leg absent and tarsomere I not modified in males. Aedeagus (Fig. 32C, D) extremely slender in dorsal view, 8.6 × longer than wide, sides asymmetric, curved at middle, recurved near apex, apex narrowly rounded; straight but strongly curved near base in lateral view, apex narrowly rounded; ostium not covered by membrane; two elongate endophallic sclerite, several fine teeth on apex of primary endophallic sclerite, 0.6 × as long as aedeagus, secondary sclerite much shorter, 0.6 × as long as primary endophallic sclerite, apex narrowly rounded. Sclerotized gonocoxae (Fig. 32I) transverse, both gonocoxae basally connected and membranous, with several short and long setae near apices. Ventrite VIII (Fig. 32E) transverse; disc with dense, short and few longer setae along apical margin; spiculum long. Receptacle of spermatheca (Fig. 32F) slightly swollen; pump short and strongly curved; sclerotized proximal spermathecal duct wide and short. Apical margin of abdominal ventrite V slightly concave medially, with deep, triangular depression at middle in males (Fig. 32H); only slightly concave in females (Fig. 32G).

#### Variation.

Taiwanese populations display great variation of color patterns on the elytra. Some individuals have two additional transverse black stripes (Fig. 31D): anterior stripe at basal 1/5, with a longitudinal black stripe at middle, anteriorly connected with basal stripe; posterior stripe at middle. Some individuals have a black stripe along suture expanding laterally at base (Fig. 31E), sometimes covering entire base, and gradually narrowing towards apex (Fig. 31F).

#### Remarks.

adults of *P. discalis* Gressitt and Kimoto are easily recognized by the yellowish brown bodies. In males of *P. discalis*, the elongate and apically curved aedeagus is similar to that of *P. semifulva* (Jacoby), but differs by the relatively shorter secondary endophallic sclerite, 0.6 × as long as primary endophallic sclerite (Fig. 32C, D) (relatively longer secondary endophallic sclerite, 0.9 × as long as primary endophallic sclerite in *P. discalis* (Fig. 29C, D).

#### Host plants.

**Figure 33.**
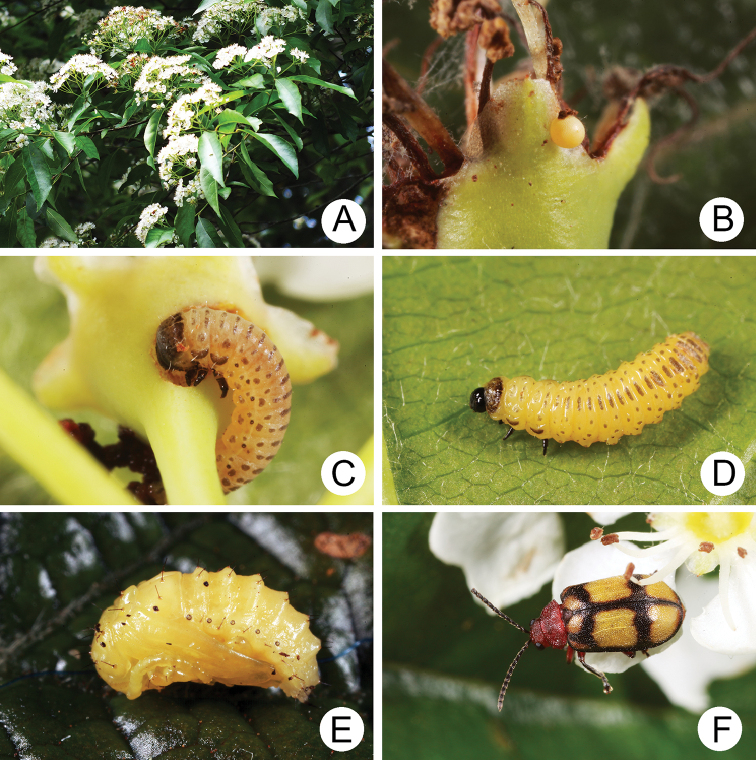
Field photographs of *Pyrrhalta discalis* Gressitt & Kimoto on host plant **A** host plant, *Pourthiaea lucida***B** egg **C** Three-instar larva **D** mature larva **E** pupa **F** adult.

Larvae and adults feed on flowers of *Pourthiaea lucida* Decne. (Fig. 33A) and *Pyracantha koidzumii* (Hayata) Rehder (Rosaceae).

#### Biology.

eggs (Fig. 33B), mature larvae (Fig. 33C), and adults (Fig. 33F) were found on flowers of *Pourthiaea lucida* April 14, 2011 in Yongfu, northern Taiwan by Mr Mei-Hua Tsou. mature larvae (Fig. 33D) burrowed into soil and built underground chambers for pupation at the same day. Duration of the pupal stage (Fig. 33E) was 14 days.

#### Distribution.

China, Taiwan. It is widespread at lowlands (0–1,500 m) in Taiwan.

### Pyrrhalta ishiharai

Taxon classificationAnimaliaColeopteraChrysomelidae

Kimoto, 1976

5ACDE192-3ED2-51A5-8221-A192D338A26A

**Figure 34.**
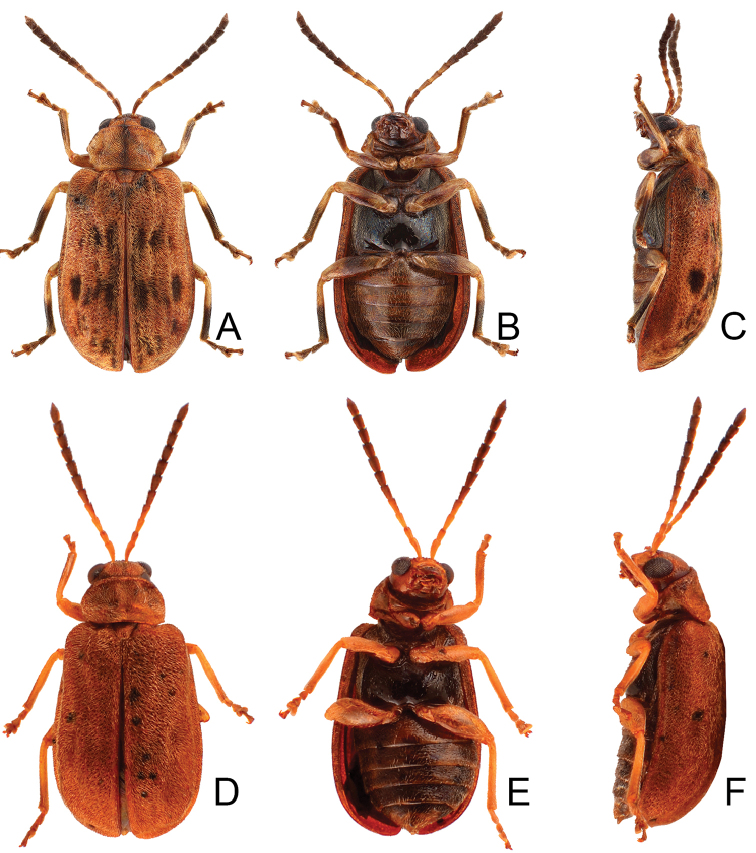
Habitus of *Pyrrhalta ishiharai* Kimoto and *P. wulaiensis* sp. nov. **A***P. ishiharai*, female, typical form, dorsal view **B** ditto, ventral view **C** ditto, lateral view **D***P. wulaiensis* sp. nov., female, dorsal view **E** ditto, ventral view **F** ditto, lateral view.

**Figure 35.**
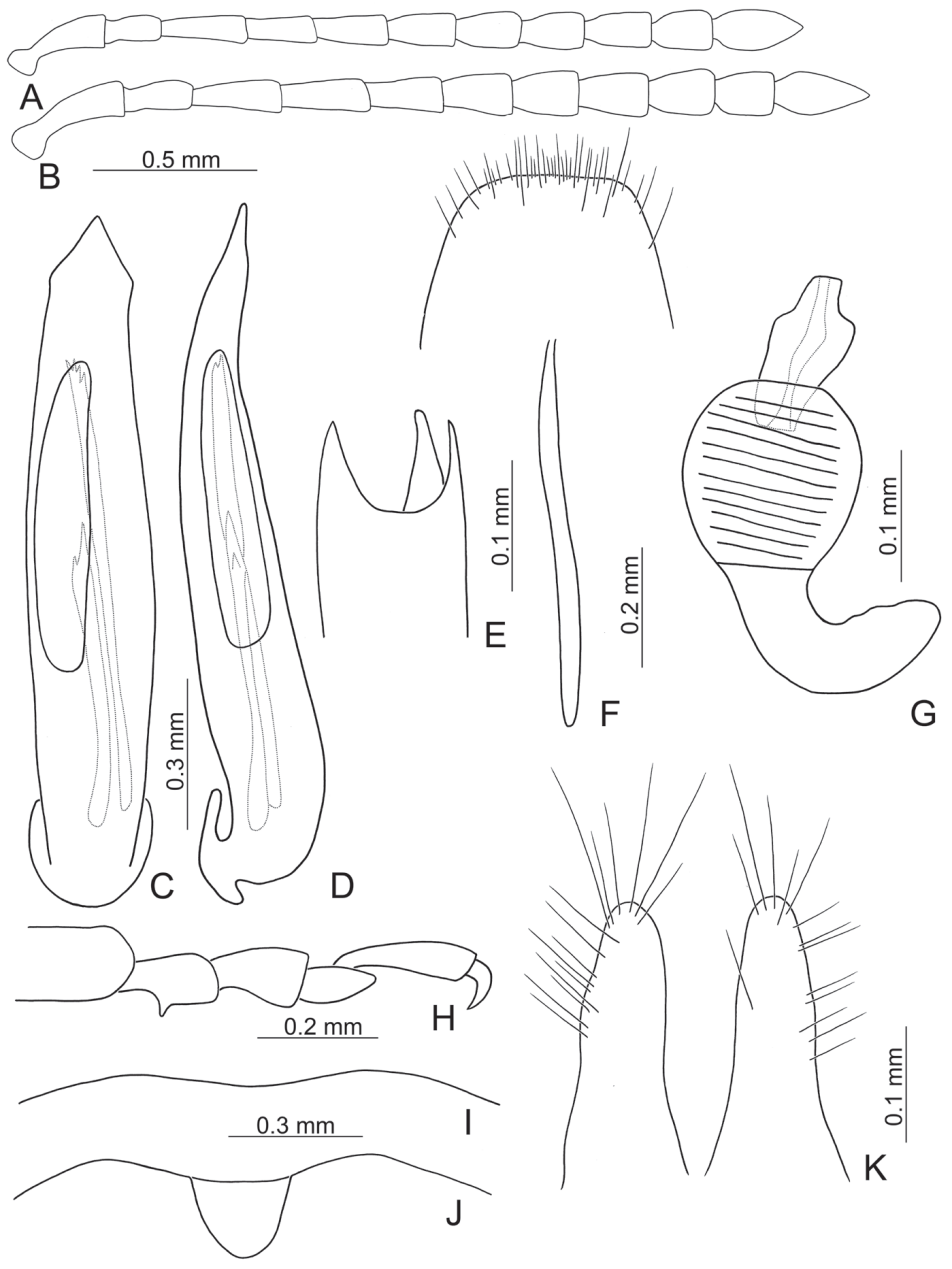
Diagnostic characters of *Pyrrhalta ishiharai* Kimoto **A** antenna, male **B** antenna, female **C** aedeagus, dorsal view **D** ditto, lateral view **E** apex of tibia of middle leg, male **F** abdominal ventrite VIII **G** spermatheca **H** tarsi of middle leg, male **I** abdominal ventrite V, female **J** abdominal ventrite V, male **K** gonocoxae.

Figs 26D , 34A–C , 35 , 37F

Pyrrhalta aurata : Kimoto, 1976: 4 (Taiwan). Misidentification (after Kimoto 1994)!
Pyrrhalta ishiharai Kimoto, 1994: 191; Kimoto and Chu 1996: 56 (catalogue); Kimoto and Takizawa 1997: 300 (key), 373; Beenen 2010: 452 (catalogue); Xue and Yang 2010: 124 (catalogue); Yang et al. 2015: 117 (catalogue).

#### Types.

***Holotype*** ♀ (EUMJ, by original designation): “(TAIWAN) / Kueishan [龜山] / ~ Wulai [烏來] / Taipei Hsien / 5. VI, 1970 / Y. Hori leg. [p, w] // Pyrrhalta / ishiharai / Kimoto, n. sp [h] / Det. S. Kimoto, 19[p]93 [h, w] // msp [h, w] // PHOTO [p, r] // HOLOTYPE [p, r]”. ***Paratype*.** 1♀ (KMNH): “NANSHANCHI [南山溪] / TAIWAN / 2. V. 1982 / F. KIMURA [p, y] // Pyrrhalta / ishiharai / Kimoto, n. sp [h] / Det. S. Kimoto, 19[p]93 [h, w] // PARATYPE [p, b]”.

#### Other material.

Taiwan. Hsinchu: 1♂, 1♀ (TARI), Chienshih (尖石), 10.VII.2010, leg. M.-H. Tsou; 1♂ (TARI), same locality, 5.VIII.2012, leg. Y.-L. Lin; Nantou: 1♂ (NMNS), Chunyang (春陽), 7.I. –13.II.2003, leg. C. S. Lin & W. T. Yang; Taipei: 2♀ (TARI), Fushan (福山), 26.VI.2011, leg. M.-H. Tsou; 2♂, 1♀ (TARI), same but with “8.VII.2011”; 3♂, 3♀ (TARI), same but with “21.VI.2015”; 2♂, 2♀ (TARI), Hsinhsien (信賢), 8.VII.2011, leg. M.-H. Tsou; 4♂, 3♀ (TARI), same but with “6.VII.2019”; 4♂, 2♀ (TARI), same but with 7.VII.2019”; 3♂ (TARI), same but with “27.VI.2020”; 1♂, 1♀ (TARI), same but with “5.VII.2020”; 4♂, 3♀ (TARI), Wulai (烏來), 8.VII.2011, leg. M.-H. Tsou; 1♂, 1♀ (TARI), same but with “17.VI.2018”; 1♂, 7♀ (TARI), same but with “27.VI.2020”; 5♂, 8♀ (TARI), same but with “5.VII.2020”; Taitung: 2♂ (TARI), Liyuan (栗園), 19.VI.2013, leg. Y.-T. Chung; 2♂ (TARI), same but with “leg. B.-X. Guo”.

#### Redescription.

Length 4.8–5.1 mm, width 2.3–2.5 mm. Body color (Fig. 34A–C) yellowish brown; vertex with one dark spot at center; antennae dark brown, but four or five basal antennomeres basally paler; pronotum with three black spots, one elongate spot at center, one pair laterally; scutellum basally darker; four pairs of transverse dark spots on elytra, one pair near base and behind scutellum, three pairs at basal 2/5, 3/5, 4/5 respectively, intercepted by two pairs of longitudinal yellowish brown ridges, all dark spots poorly defined; meso- and metathoracic ventrites darker; apical 2/3 of tibiae and entire tarsi black except inner side of protibia. Eyes small, interocular space 2.35–2.38 × diameter of eye. Antennae filiform in males (Fig. 35A), length ratios of antennomeres I–XI 1.0: 0.6: 0.9: 0.7: 0.7: 0.7: 0.6: 0.6: 0.6: 0.6: 0.9, length to width ratios of antennomeres I–XI 3.1: 2.1: 3.2: 2.5: 2.4: 2.1: 1.8: 1.8: 1.8: 1.6: 2.2; filiform in females (Fig. 35B), length ratios of antennomeres I–XI 1.0: 0.6: 0.7: 0.7: 0.6: 0.6: 0.6: 0.6: 0.5: 0.5: 0.8, length to width ratios of antennomeres I–XI 3.4: 2.5: 2.9: 2.8: 2.2: 1.7: 1.6: 1.6: 1.5: 1.4: 2.3. Pronotum and elytra convex. Pronotum 1.7–1.9 × wider than long, disc with reticulate microsculpture; coarse, extremely dense punctures, and extremely short pubescence; with median longitudinal and lateral depressions; lateral margins angular, widest at apical 1/3, apical and basal margins truncate; posterior setiferous punctures strongly erect. Elytra elongate, broad, parallel-sided, 1.5 × longer than wide; disc with reticulate microsculpture and coarse, extremely dense punctures and short pubescence; with two pairs of long longitudinal ridges near suture, apically abbreviated; several oblique ridges exterior to longitudinal ridges. Apical spur of middle tibia of middle small (Fig. 35E), tarsomere I with a small tooth at middle ventrally in males (Fig. 35H). Aedeagus (Fig. 35C, D) slender in dorsal view, 5.9 × longer than wide, sides asymmetric, widest at middle, apex angular; strongly curved near base in lateral view, weakly recurved apically, apex acute; ostium longitudinal, not covered by membrane; two endophallic sclerites elongate, apex of primary endophallic sclerite with several teeth, 0.6 × as long as aedeagus, secondary sclerite much shorter, 0.7 × as long as primary sclerite, apex acute, with one additional tooth near apex. Only apices of gonocoxae (Fig. 35K) sclerotized and longitudinal, with dense, long setae along lateral and apical margins. Ventrite VIII (Fig. 35F) narrow; disc with several long setae and dense short setae along apical margin; spiculum long. Receptacle of spermatheca (Fig. 35G) very swollen; pump short and strongly curved; sclerotized proximal spermathecal duct wide and short. Apical margin of abdominal ventrite V slightly concave, with deeply rounded depression at middle in males (Fig. 35J); slightly concave in females (Fig. 35I).

#### Remarks.

**Figure 36.**
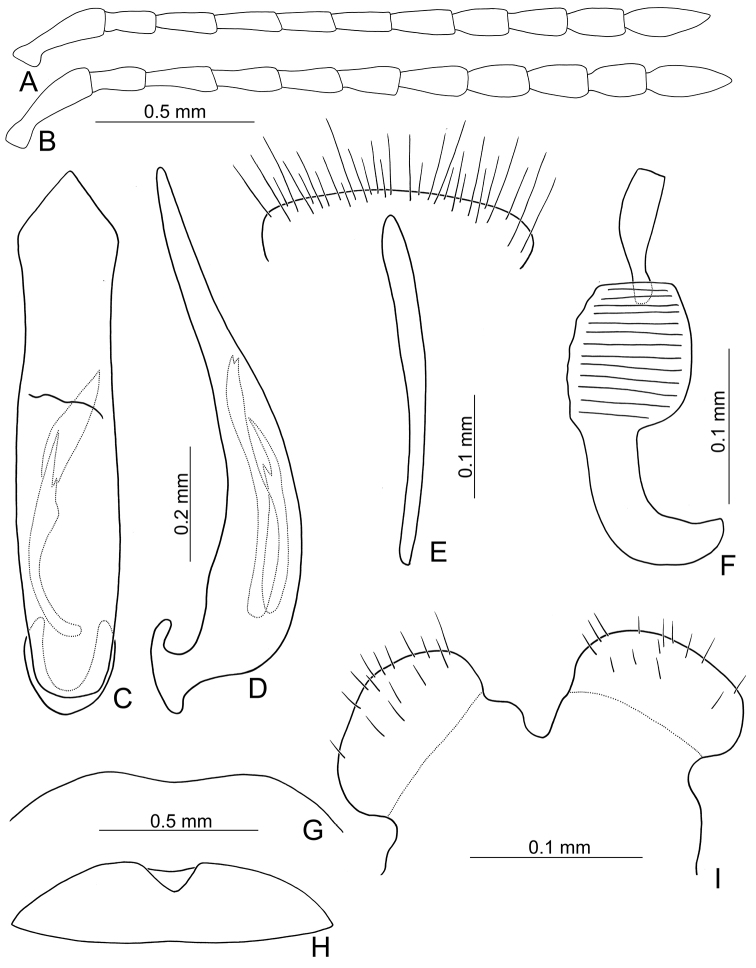
Diagnostic characters of *Pyrrhalta wulaiensis* sp. nov. **A** antenna, male **B** antenna, female **C** aedeagus, dorsal view **D** ditto, lateral view **E** abdominal ventrite VIII **F** spermatheca **G** abdominal ventrite V, female **H** abdominal ventrite V, male **I** gonocoxae.

**Figure 37.**
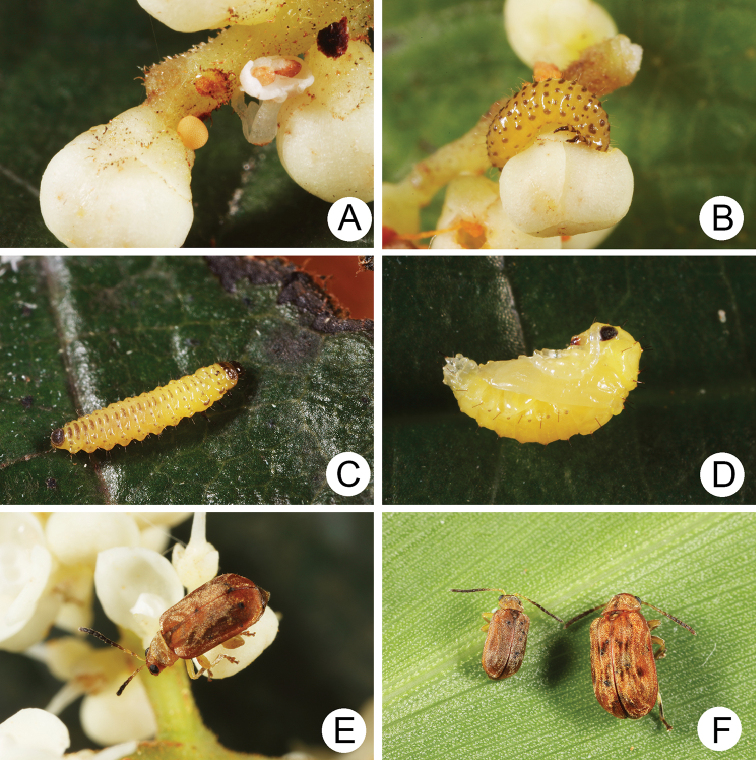
Field photographs of *Pyrrhalta wulaiensis* sp. nov. on host plant **A** egg **B** third-instar larva **C** mature larva **D** pupa **E** adult **F** adults: *P. wulaiensis* sp. nov. (left) and *P. ishiharai* (right).

Adults of *P. ishiharai* Kimoto and *P. wulaiensis* sp. nov. are easily separated from other species within the species group by the longitudinal ridges on the elytra (Fig. 34) and the angular apices of the aedeagi (Figs 35C, 36C). *Pyrrhalta ishiharai* is distinguished from *P. wulaiensis* sp. nov. by the larger body size (Fig. 37F), 4.8–5.1 mm long (3.3–3.7 mm long in *P. wulaiensis* sp. nov.), dark spots present between the longitudinal ridges on the elytra (Fig. 34A) (dark spots absent between longitudinal ridges on elytra in *P. wulaiensis* sp. nov. Fig. 34D), apical spine present on tibia (Fig. 35E) and modified tarsomere I of middle leg (Fig. 35H) in males (lacking apical spine on tibia and normal tarsomere I of middle leg in males of *P. wulaiensis* sp. nov.), longitudinal ostium and aedeagus recurved in apical 1/3 (Fig. 35C, D) (transverse ostium and aedeagus curved at middle in *P. wulaiensis* sp. nov. (Fig. 36C, D)), longitudinally cylindrical gonocoxae with dense, long setae (Fig. 35K) (transversely rounded gonocoxae with scattered short setae in *P. wulaiensis* sp. nov. (Fig. 36I)).

#### Food plant.

Adults feed on flowers of *Meliosma rhoifolia* Maxim. (Sabiaceae) (Fig. 26D).

#### Distribution.

The species is widespread at lowlands (0–1,500 m) in Taiwan.

### Pyrrhalta wulaiensis sp. nov.

Taxon classificationAnimaliaColeopteraChrysomelidae

51B26CF8-688E-544B-BECB-422E3CD49B1C

http://zoobank.org/ECCE0D20-4E5F-4CC3-B17B-24BBE0BAF34F

Figs 34D–F , 36 , 37A–E

#### Types.

***Holotype*** ♂ (TARI), Taiwan. Nantou: Peitungyanshan (北東眼山), 3.VII.2014, leg. F.-S. Huang, 變葉新木薑子 (Neolitsea aciculata (Bl.) Koidz. var. variabillima J.C. Liao) 噴霧 (fogging). ***Paratypes*.** 1♂ (TARI), same locality as holotype, 3.VII.2014, leg. C.-F. Lee; Ilan: 1♂ (TARI), Fushan (福山), 5.VII.2013, leg. Y.-T. Wang; Miaoli: 1♂ (TARI), Hsuehchien (雪見), 5.III.2013, leg. W.-B. Yeh; Nantou: 1♀ (TARI), Meifeng (梅峰), 28–29.VIII.1981, leg. L. Y. Chou & S. C. Lin; 1♀ (TARI), same locality, 15.VII.1982, leg. S. C. Lin & C. N. Lin; 1♀ (NMNS), same locality, 13.VI. –18.VII.2001, leg. C. S. Lin & W. T. Yang, Malaise trap (KCN); 1♂ (NMNS), same but with “15.XI. –19.XII.2001”; 1♀ (NMNS), same but with “5.X. –16.XI.2004”; Taipei: 1♂ (TARI), Fushan (福山) – 烏來 (Wulai), 21.VI.2015, leg. M.-H. Tsou; 1♂ (TARI), Hsinhsien (信賢), 5.VII.2020, leg. M.-H. Tsou; 1♀ (TARI), same but with “27.VI.2020”; 1♀ (TARI), Wulai (烏來), 19.VII.2011, leg. M.-H. Tsou.

#### Diagnosis.

Smaller species, 3.3–3.7 mm in length. Elytra relatively broad, 1.5 × longer than wide; unicolorous, without dark spots; with ridges.

#### Description.

Length 3.3–3.7 mm, width 1.6–1.9 mm. Body color (Fig. 34D–F) brown or dark brown; antennae black but antennomeres I–III yellow, IV, and V brown. Eyes large, interocular space 1.75–1.83 × diameter of eye. Antennae filiform in males (Fig. 36A), length ratios of antennomeres I–XI 1.0: 0.5: 0.6: 0.7: 0.6: 0.6: 0.7: 0.6: 0.6: 0.6: 0.9, length to width ratios of antennomeres I–XI 3.2: 2.2: 2.9: 3.0: 2.9: 2.9: 2.5: 2.0: 2.1: 2.0: 2.9; similar in females (Fig. 36B), length ratios of antennomeres I–XI 1.0: 0.5: 0.7: 0.7: 0.6: 0.6: 0.7: 0.6: 0.6: 0.6: 0.8, length to width ratios of antennomeres I–XI 3.5: 2.3: 2.9: 2.8: 2.5: 2.2: 2.3: 1.9: 1.8: 1.8: 2.7. Pronotum and elytra convex. Pronotum 1.7–2.0 × wider than long, with transverse ridge along apical margin deflexed at antero-lateral angles; disc smooth on ridge, but with reticulate microsculpture below ridge, with extremely dense and coarse punctures, with one short seta at each puncture; with median longitudinal and lateral depressions; lateral margins moderately rounded, widest at apical 1/3, apical and basal margins slightly concave; posterior setiferous punctures slightly erect. Elytra elongate and broad, parallel-sided, 1.5 × longer than wide; disc with reticulate microsculpture, and with coarse and sparse punctures, with extremely dense short pubescence, all of pubescence located between punctures; with indistinct, obliquely longitudinal ridges arising from behind humeral calli, with depressions between ridges and suture at apical 1/3 and middle. Apical spur of tibia of middle leg absent and tarsomere I not modified in males. Aedeagus (Fig. 36C, D) slender in dorsal view, 5.9 × longer than wide, sides symmetric, parallel-sided but slightly narrowed at apical 1/4, apex angular; strongly curved near base in lateral view, apex acute; ostium transverse, covered by a membrane; two endophallic sclerite elongate, apex of primary endophallic sclerite with two teeth, 0.4 × as long as aedeagus, secondary sclerite 0.8 × as long as primary sclerite, apex acute, with one additional tooth at apical 1/4. Only apices of gonocoxae (Fig. 36I) sclerotized and transverse, with short, scattered setae. Ventrite VIII (Fig. 36E) with only apical area sclerotized; disc with several long setae and dense short setae along apical margin; spiculum long. Receptacle of spermatheca (Fig. 36F) very swollen; pump short and strongly curved; sclerotized proximal spermathecal duct narrow and short. Apical margin of abdominal ventrite V slightly concave, with shallow triangular depression at middle in males (Fig. 36H); slightly concave in females (Fig. 36G).

#### Remarks.

Adults of *P. wulaiensis* sp. nov. and *P. ishiharai* Kimoto are easily separated from other species within the species group by the longitudinal ridges on the elytra (Fig. 34) and the angular apices of aedeagi (Figs 35C, 36C). *Pyrrhalta wulaiensis* sp. nov. is distinguished from *P. ishiharai* by the smaller body size (Fig. 37F), 3.3–3.7 mm long (4.8–5.1 mm long in *P. ishiharai*), absence of dark spots between the longitudinal ridges on the elytra (Fig. 34D) (dark spots present between longitudinal ridges on elytra in *P. ishiharai* Fig. 34A), lacking apical spine on tibia and normal tarsomere I of middle leg in males (apical spine present on tibia (Fig. 35E) and modified tarsomere I of middle leg in males of *P. ishiharai* (Fig. 35H)), transverse ostium and medially curved aedeagus (Fig. 36C, D) (in longitudinal ostium and recurved at apical 1/3 of aedeagus *P. ishiharai* (Fig. 35C, D)), transversely rounded gonocoxae with scattered short setae (Fig. 36I) (longitudinally cylindrical gonocoxae with dense, long setae in *P. ishiharai* (Fig. 35K))

#### Host plant.

Larvae and adults feed on flowers of *Meliosma rhoifolia* Maxim. (Sabiaceae).

#### Biology.

One female was collected on flowers of the host plant (Fig. 37E) July 8, 2011 in Wulai, northern Taiwan by Mr Mei-Hua Tsou. The female deposited eggs (Fig. 37A) singly on flowers July 12. Larvae hatched in seven days. The larvae (Fig. 37B) fed on flowers and the larval duration was eleven days. mature larvae (Fig. 37C) burrowed into soil and built underground chambers for pupation. Duration of the pupal stage (Fig. 37D) was eight days.

#### Distribution.

The species is widespread at lowlands (0–1,500 m) in northern Taiwan and mid-altitudes (1,500–2,500 m) in central Taiwan.

#### Etymology.

The species is named for the locality where specimens were collected and used for laboratory rearing.

### Pyrrhalta shirozui

Taxon classificationAnimaliaColeopteraChrysomelidae

species group

5113CA48-E4C9-5B9C-8BF0-97115B9B68F8

#### Included species.

*Pyrrhalta jungchani* sp. nov.; *P. lui* sp. nov.; and *P. shirozui* Kimoto, 1969.

#### Diagnosis.

**Figure 38.**
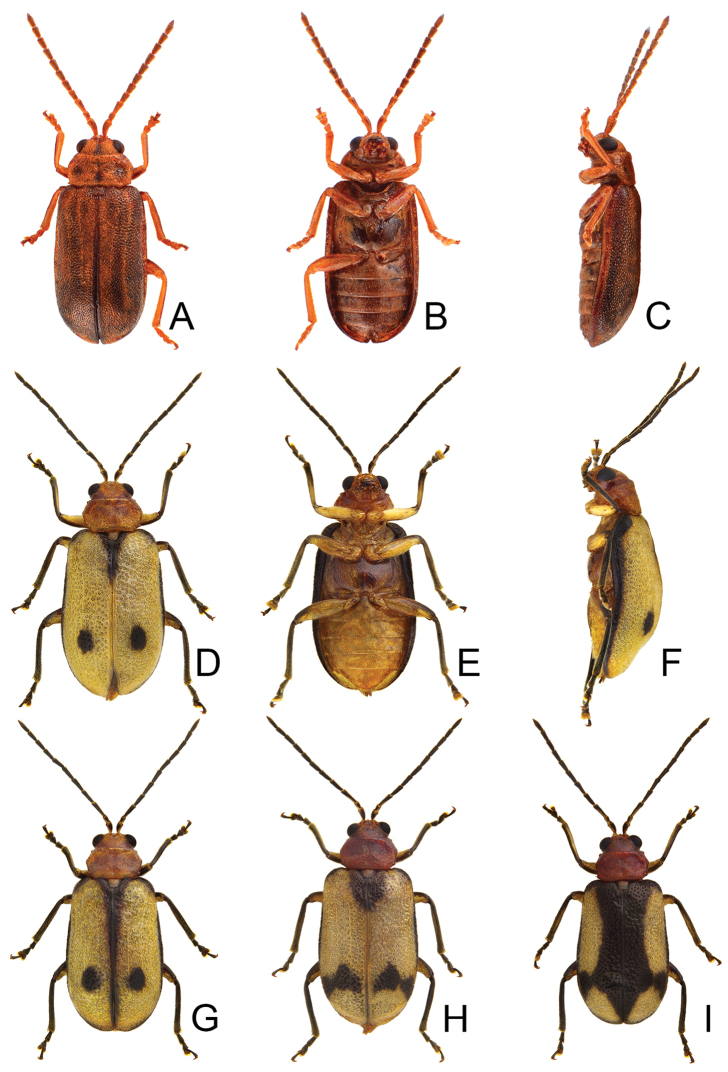
Habitus of *Pyrrhalta jungchani* sp. nov. *and P. shirozui* Kimoto **A***P. jungchani* sp. nov., male, dorsal view **B** ditto, ventral view **C** ditto, lateral view **D***P. shirozui*, female, dorsal view **E** ditto, ventral view **F** ditto, lateral view **G** Same species, color variation **H** Same species, color variation **I** Same species, color variation.

**Figure 39.**
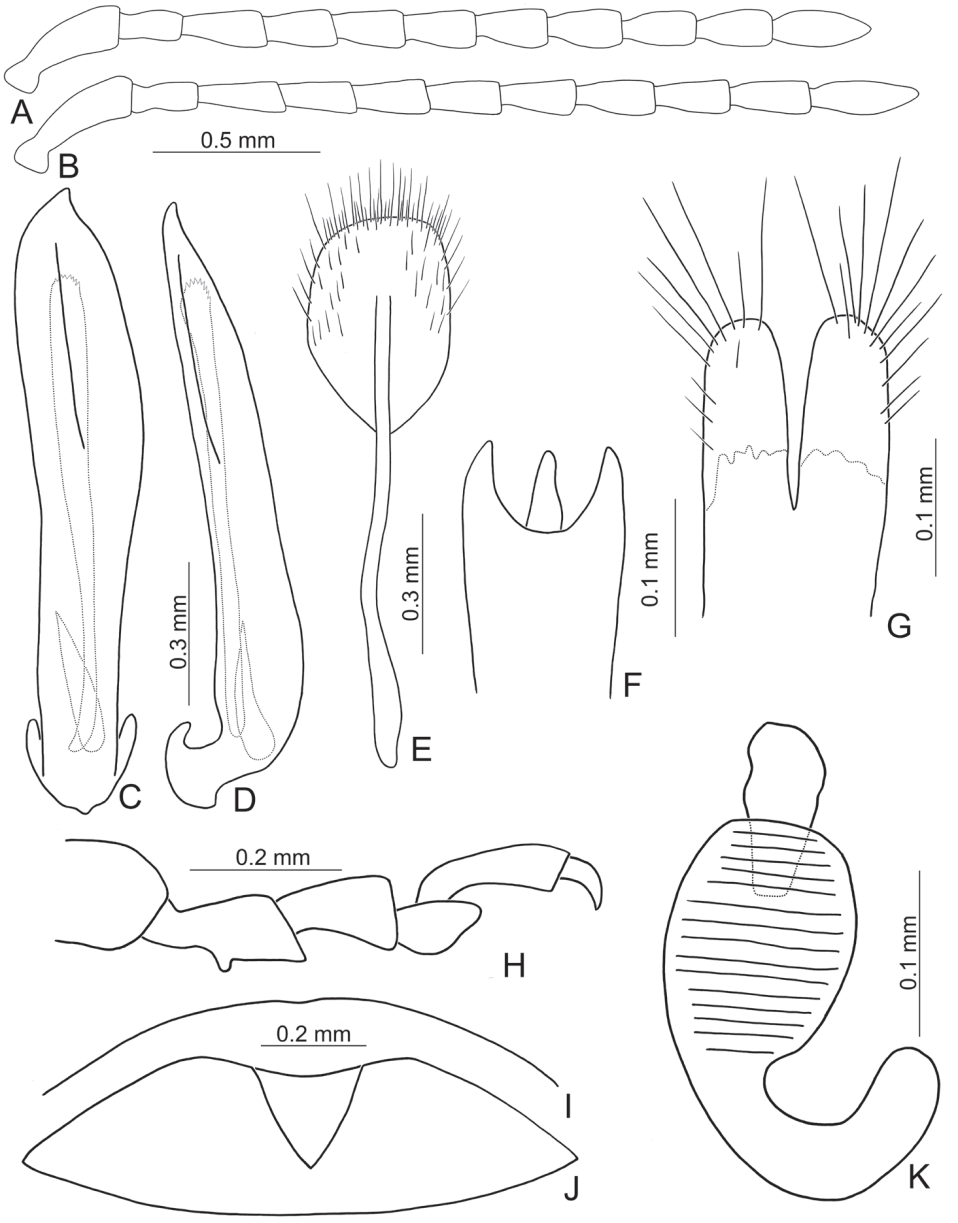
Diagnostic characters of *Pyrrhalta jungchani* sp. nov. **A** antenna, male **B** antenna, female **C** aedeagus, dorsal view **D** ditto, lateral view **E** abdominal ventrite VIII **F** apex of tibia of middle leg, male **G** gonocoxae **H** tarsi of middle leg, male **I** abdominal ventrite V, female **J** abdominal ventrite V, male **K** spermatheca.

adults small to medium sized (3.3–6.8 mm). Antenna slender, antennomere III longest, V–X similar in size. Body convex. Elytra relatively wider for *P. shorozui* 1.5 × longer than wide (Fig. 38D–I), or relatively narrow for *P. jungchani* sp. nov. and *P. lui* sp. nov., 1.7–1.8 × longer and wide (Figs 38A–C, 41). Aedeagus asymmetric; ostium longitudinal, covered by a membrane or without cover; endophallic sclerites composed of two slender sclerites, with several teeth on apex of primary sclerite (Figs 39C, D, 42C, D, 43C, D). Ventrite VIII in females apically sclerotized, with dense, mixed short and long setae along apical margin; spiculum long (Figs 39E, 42E, 43F). Gonocoxae apically sclerotized, with dense, long setae on apices (Figs 39G, 42I, 43G). Apical margin of abdominal ventrite V slightly concave medially and with deep depression in males (Figs 39J, 42H, 43J); depression broadly rounded in females (Figs 39I, 42G, 43I). Mesotibia with apical spine in males of *P. jungchani* sp. nov. (Fig. 39F) and *P. shirozui* (Fig. 43E). Mesotarsi with tarsomere I modified in males of *P. jungchani* sp. nov. (Fig. 39H) and *P. shirozui* (Fig. 43K).

#### Biology.

Larvae and adults feed on leaves of *Viburnum* species (Adoxaceae).

### Pyrrhalta jungchani sp. nov.

Taxon classificationAnimaliaColeopteraChrysomelidae

8C1AABBA-CC66-5D57-90CB-F697C337C4D6

http://zoobank.org/4C599464-5532-40EB-B379-B4C92C03ABE2

**Figure 40.**
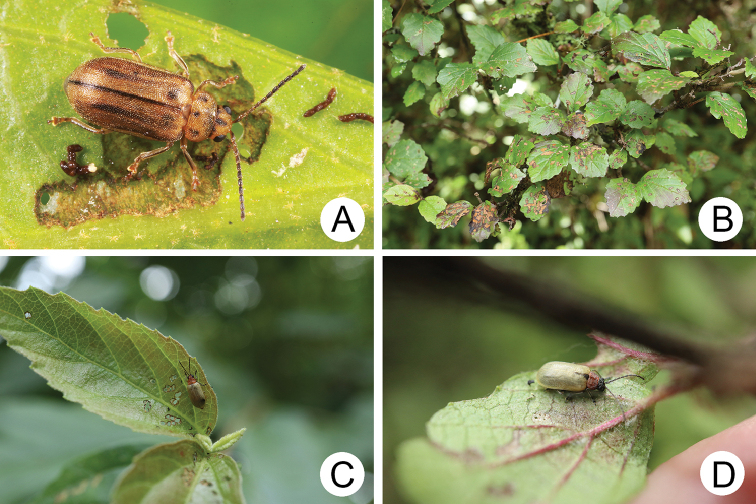
Field photographs of *Pyrrhalta jungchani* sp. nov. and *P. lui* sp. nov. on host plant **A** adult of *P. jungchani* sp. nov. **B** host plant for *P. lui* sp. nov., *Viburnum parvifolium* with feeding marks **C** adult of *P. lui* sp. nov. feeding on leaves of *V. luzonicum***D** adult of *P. lui* sp. nov.

Figs 38A–C , 39 , 40A

#### Types.

***Holotype*** ♂ (TARI), Taiwan. Pingtung, Tahantrail (大漢林道), 30.VII.2012, leg. J.-C. Chen. ***Paratypes*.** Chiayi: 1♀ (TARI), Zengwen Reservoir (曾文水庫), 2.IV.2016, leg. U. Ong; Nantou: 2♀ (NMNS), Juiyenhsi (瑞岩溪), Shuikuan Road (水管路), 19.II.2009, Beating,leg. M. L. Chan; 1♂ (NMNS), same locality, 19–21.II.2009, UV light trap, leg. H. H. Lian & C. D. Tang; 1♀ (TARI), Tsuifeng (翠峰), 23.V.1982, leg. L. Y. Chou; 1♀ (TARI), same locality, 20.IV.1983, leg. K. C. Chou & S. P. Huang; 1♀ (TARI), same as holotype but with “22.XI.2010”; 1♀ (TARI), same but with “13.IX.2012”; 1♀ (TARI), same but with “16.X.2013”; Pingtung: 1♀ (TARI), Lilungshan (里龍山), 5.XI.2009, leg. M.-H. Tsou; 1♀ (TARI), same locality, 28.XI.2009, leg. J.-C. Chen; 1♀ (TARI), same but with “8.I.2010”; 1♂, 1♀ (TARI), same but with “2.III.2012”; 3♀ (TARI), same but with “13.III.2012”; 1♀ (TARI), same but with “27.III.2012”; 1♀ (TARI), same but with “8.I.2013”; 2♂, 4♀ (TARI), Shuangliu (雙流), 14.III.2018, leg. Y.-T. Chung; 2♂, 2♀ (TARI), Tahanshan (大漢山), 18.IV.2018, leg. C.-F. Lee; 1♀ (TARI), same locality, 3.III.2020, leg. Y.-T. Chung; Taitung: 1♀ (TARI), Hsiangyang (向陽), 23.VI.2010, leg. M.-H. Tsou; 1♀ (TARI), same locality, 8.VII.2010, leg. J.-C. Chen; 1♂ (TARI), same but with “12.VII.2012”; 1♀ (TARI), same but with “9.V.2013”; 1♀ (TARI), same but with “17.V.2014”; 1♀ (TARI), Motien (摩天), 23.VI.2010, leg. M.-H. Tsou.

#### Diagnosis.

Small species, 4.3–5.0 mm in length. Pronotum with three large black spots, one at middle, two laterally. Elytra relatively narrow, 1.7 × longer than wide, disc with dense coarse punctures, with black stripes at humeral calli, with one additional pair of longitudinal dark stripes between humeral calli and suture.

#### Description.

Length 4.3–5.0 mm, width 1.9–2.3 mm. Body yellowish brown (Fig. 38A–C); vertex with median longitudinal dark stripe, expanding laterally at base; antennae dark brown, but five or six basal antennomeres paler; pronotum with one pair of large dark spots at lateral depressions, with three small dark spots medially, one pair at apical 1/3, the other at basal 1/3; elytra with three pairs of longitudinal dark stripes, first pair arising from humeral calli, abbreviated at apical 1/3, second pair halfway between first pair and suture, present from base to apical 1/3; third pair along suture, from apical 1/3 to base; those dark spots or stripes more or less reduced in different individuals; lateral margins of femora and tibiae dark brown. Eyes small, interocular space 2.34–2.68 × diameter of eye. Antennae filiform in males (Fig. 39A), length ratios of antennomeres I–XI 1.0: 0.5: 0.6: 0.5: 0.5: 0.5: 0.5: 0.5: 0.6: 0.6: 0.8, length to width ratios of antennomeres I–XI 2.9: 2.1: 2.2: 1.9: 1.9: 1.9: 1.9: 2.0: 2.0: 2.1: 2.7; similar in females (Fig. 39B), length ratios of antennomeres I–XI 1.0: 0.5: 0.6: 0.5: 0.5: 0.5: 0.5: 0.6: 0.5: 0.6: 0.8, length to width ratios of antennomeres I–XI 3.3: 2.2: 2.7: 2.3: 2.2: 2.1: 2.1: 2.4: 2.3: 2.4: 3.3. Pronotum and elytra convex. Pronotum 2.0–2.1 × wider than long, with transverse ridge along apical margin deflexed at antero-lateral angles; disc with reticulate microsculpture, and extremely coarse, dense punctures, with one extremely short seta at each puncture; with median longitudinal and lateral depressions; lateral margins moderately rounded, widest at middle, apical and basal margins slightly concave; anterior and posterior setiferous punctures not erect. Elytra broad, parallel-sided, 1.7 × longer than wide; disc smooth, with extremely coarse and dense punctures, and sparse, extremely short pubescence, usually located between punctures; with indistinct ridges along dark stripes. Apical spur of tibia of middle leg small (Fig. 39F), and tarsomere I modified, axe-shaped in lateral view, narrow basally, and extending to apical 2/3, angles of extended part narrowly rounded in males (Fig. 39H). Aedeagus (Fig. 39C, D) slender in dorsal view, 5.3 × longer than wide, asymmetrically lanceolate, slightly curved at middle, strongly narrowed and recurved near apex, apex narrowly rounded; ostium obliquely longitudinal, covered by a membrane; strongly curved near base in lateral view, recurved near apex, apex narrowly rounded; two endophallic sclerites elongate, primary sclerite 0.8 × as long as aedeagus, with dense teeth along apical margin. Secondar sclerite much shorter, 0.3 × as long as secondary sclerite, apex acute. Only apices of gonocoxae (Fig. 39G) sclerotized, longitudinal, few short setae near base, with six to seven long setae near apex of each gonocoxa. Ventrite VIII (Fig. 39E) well sclerotized, with dense, short setae along lateral and apical area, short and long marginal setae, spiculum long. Receptacle of spermatheca (Fig. 39K) slightly swollen and elongate; pump short and strongly curved; sclerotized proximal spermathecal duct narrow and short. Apical margin of abdominal ventrite V slightly concave medially and with deep depression in males (Fig. 39J); while broadly rounded in females (Fig. 39I).

#### Remarks.

Adults of *P. jungchani* sp. nov. (Fig. 38A), *X. aenescens* (Fairmaire) (Fig. 1D), and *P. lineatipes* (Takei) (Fig. 45G) are easily recognized by the three black spots on the pronota. This new species (Fig. 38C) is most similar to *P. lineatipes* (Fig. 45I) based on the brown elytra with a black stripe arising from the humeral calli and convex pronotum and elytra (entirely metallic green elytra and dorso-ventrally flattened pronotum and elytra in *X. aenescens* (Fig. 1F)). The new species is different from *P. lineatipes* in possessing sparse pubescence and extremely dense elytral punctures (dense pubescence with sparse elytral punctures in *P. lineatipes*), and modified tarsomere I of middle leg in males (Fig. 39H) (normal tarsomere I of middle leg in males of *P. humeralis*). In males of this new species, the aedeagus (Fig. 39C, D) is similar to that of *P. lui* sp. nov. (Fig. 40C, D) with the asymmetrically lanceolate shape and two endophallic sclerites but differs in the recurved apex and shorter secondary endophallic sclerite, 0.3 × as long as primary endophallic sclerite (the straight apex and the longer second endophallic, 0.6 × as long as primary endophallic sclerite, in *P. lui* sp. nov.).

#### Food plant.

adults feed on leaves of *Viburnum odoratissimum* Ker Gawl. (Adoxaceae) (Fig. 40A).

#### Distribution.

The species is widespread at mid-altitudes (1,500–2,500 m) in central and southern Taiwan.

#### Etymology.

Dedicated to Mr Jung-Chan Chen who was the first member of TCRT to collect specimens of this new species.

### Pyrrhalta lui sp. nov.

Taxon classificationAnimaliaColeopteraChrysomelidae

7DE39B0A-ABD8-5828-95F4-C082FB41121A

http://zoobank.org/22A6C67A-BA73-46F7-8E0D-A9F092F85B09

**Figure 41.**
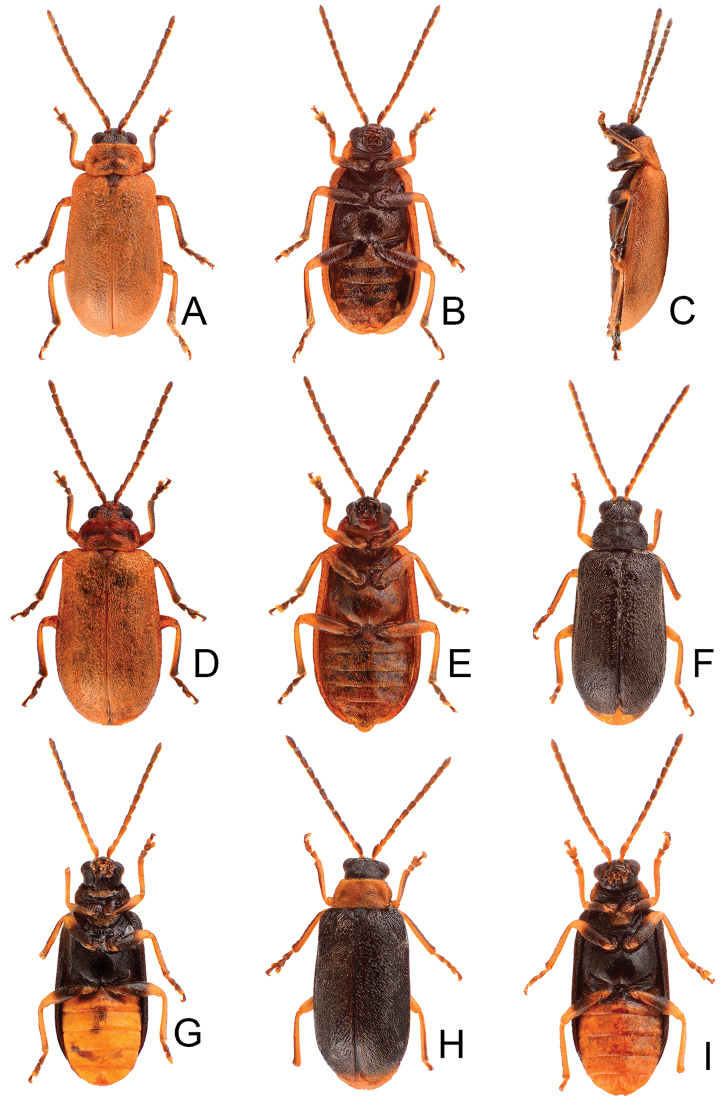
Habitus of *Pyrrhalta lui* sp. nov. **A** male, typical form, dorsal view **B** ditto, ventral view **C** ditto, lateral view **D** female, dorsal view **E** ditto, ventral view **F** male, color variation, dorsal view **G** ditto, ventral view **H** male, color variation, dorsal view **I** ditto, ventral view.

**Figure 42.**
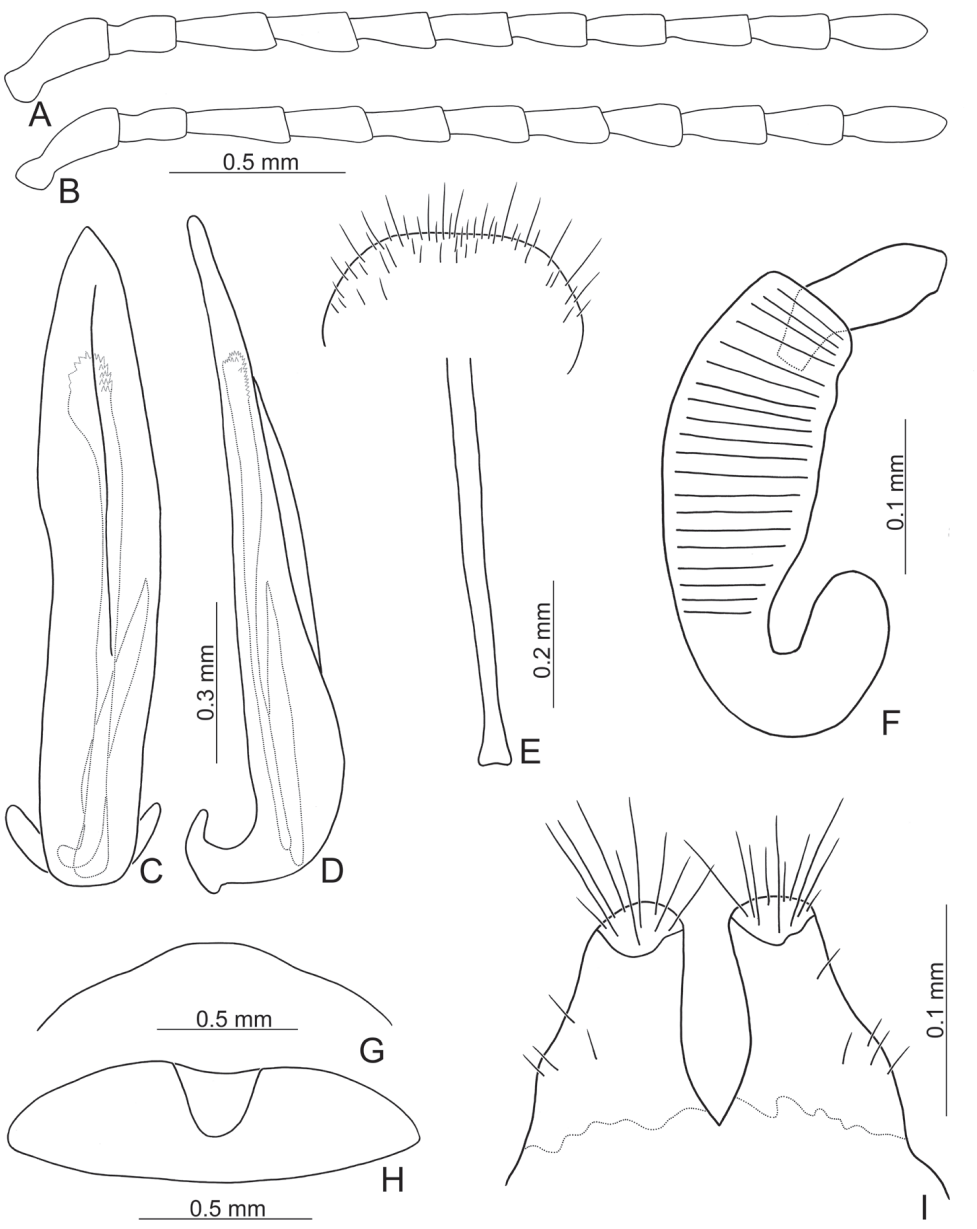
Diagnostic characters of *Pyrrhalta lui* sp. nov. **A** antenna, male **B** antenna, female **C** aedeagus, dorsal view **D** ditto, lateral view **E** abdominal ventrite VIII **F** spermatheca **G** abdominal ventrite V, female **H** abdominal ventrite V, male **I** gonocoxae.

Figs 40B–D , 41 , 42

#### Types.

***Holotype*** ♂ (TARI), Taiwan. Hualien: Hahuan Cross-Ridge (合歡越嶺古道), 4.VIII.2018, leg. H.-F. Lu. ***Paratypes*.** 16♂, 7♀ (TARI), same data as holotype; Hualien: 3♀ (TARI), Hutoushan (虎頭山), 21.IV.2018, leg. H.-F. Lu; Kaohsiung: 1♀ (TARI), Chungchihkuan (中之關), 17.IV.2012, leg. L.-P. Hsu; 4♂, 3♀ (TARI), same locality, 12.VI.2015, leg. C.-F. Lee; Miaoli: 1♂ (TARI), Hsuehchien (雪見), 7.VI.2013, leg. W.-B. Yeh; Nantou: 1♂ (TARI), Chingching (清境), 5.III.2007, leg. H.-C. Chen; 1♂ (TARI), Meifeng (梅峰), 2–4.VI.1980, leg. L. Y. Chou & C. C. Chen; 1♀ (TARI), same locality, 24–26.VI.1981, leg. K. S. Lin & W. S. Tang; 1♂ (TARI), Tatachia (塔塔加), leg. 21.VI.2009, leg. C.-F. Lee; Taichung: 1♂, 2♀ (TARI), Kukuan (谷關), 21.III.2014, leg. B.-X. Guo.

#### Diagnosis.

Elytra relatively narrow, 1.7–1.8 × longer than wide, entirely yellowish brown or black; disc smooth, lacking ridges; with sparse, fine punctures

#### Description.

Length 4.6–5.3 mm, width 2.0–2.4 mm. Body yellow, head and pronotum reddish brown, antenna blackish brown except ventral sides of five basal antennomeres, bases of femora and lateral margins of tibia black; tarsi darker in females (Fig. 41D, E); but head, scutellum, thoracic ventrites, and basal 2/3 of femora black in males (Fig. 41A–C). Eyes small, interocular space 2.55–2.85 × diameter of eye. Antennae filiform in males (Fig. 42A), length ratios of antennomeres I–XI 1.0: 0.6: 0.8: 0.7: 0.7: 0.7: 0.7: 0.7: 0.7: 0.7: 0.8, length to width ratios of antennomeres I–XI 2.6: 1.9: 2.5: 2.1: 2.2: 2.2: 2.4: 2.5: 2.8: 2.4: 2.9; similar in females (Fig. 42B), length ratios of antennomeres I–XI 1.0: 0.6: 0.9: 0.7: 0.7: 0.7: 0.7: 0.7: 0.7: 0.7: 0.9, length to width ratios of antennomeres I–XI 2.7: 2.1: 2.7: 2.2: 2.2: 2.2: 2.2: 2.1: 2.3: 2.1: 3.1. Pronotum and elytra convex. Pronotum 1.8–2.0 × wider than long, with transverse ridge along apical margin deflexed at antero-lateral angles; disc smooth on ridge, but with reticulate microsculpture below ridge, with extremely coarse punctures laterally, smaller medially, with one short seta at each puncture; with median longitudinal and lateral depressions; lateral margins moderately rounded, widest at middle, apical and basal margins slightly concave; anterior and posterior setiferous punctures not erect. Elytra broad, parallel-sided, 1.7–1.8 × longer than wide; disc smooth, with dense, coarse punctures, and extremely dense, short pubescence, some located between punctures. Apical spur of tibia of middle leg absent and tarsomere I not modified in males. Aedeagus (Fig. 42C, D) slender in dorsal view, 6.3 × longer than wide, asymmetrically lanceolate, slightly curved at middle, strongly narrower near apex, apex narrowly rounded; ostium obliquely longitudinal, covered by a membrane; strongly curved near base in lateral view, apex narrowly rounded; two endophallic sclerites elongate, primary sclerite 0.7 × as long as aedeagus, with dense teeth along apical margin. Secondary sclerite much shorter than primary sclerite, 0.6 × as long as primary sclerite, apex acute. Only apices of gonocoxae (Fig. 42I) sclerotized, longitudinal, few short setae near base, with eight to ten long setae near apex on each gonocoxa. Ventrite VIII (Fig. 42E) well sclerotized, with dense short setae along lateral and apical area, with short and long marginal setae, spiculum long. Receptacle of spermatheca (Fig. 42F) slightly swollen and elongate; pump short and strongly curved; sclerotized proximal spermathecal duct narrow and short. Apical margin of abdominal ventrite V slightly concave medially and with deep depression in males (Fig. 42H); broadly rounded in females (Fig. 42G).

#### Variation.

Males of *P. lui* sp. nov. display great variation in color. Some are totally black except for yellowish brown legs and abdomens (Fig. F, G); a few individuals are mainly black but pronota are reddish brown (Fig. 41H, I).

#### Remarks.

Adults of *P. lui* sp. nov. are distinguished within the species group by unicolorous elytra. In males, the aedeagus (Fig. 40C, D) is similar to that of *P. jungchani* sp. nov. (Fig. 39C, D), with an asymmetrically lanceolate shape and two endophallic sclerites. It differs by the straight apex and the longer secondary endophallic sclerite, 0.6 × as long as primary endophallic sclerite (recurved apex and shorter secondary endophallic sclerite, 0.3 × as long as primary endophallic sclerite, in *P. jungchani* sp. nov.).

#### Food plants.

Adults feed on leaves of *Viburnum parvifolium* Hayata (Fig. 40B) and *V. luzonicum* Rolfe (Adoxaceae) (Fig. 40C, D).

#### Distribution.

The species is widespread at mid-altitudes (1,500–2,500 m) in central and southern Taiwan.

#### Etymology.

Dedicated to Mr Hsi-Feng Lu, the member of TCRT who collected most specimens of this new species.

### Pyrrhalta shirozui

Taxon classificationAnimaliaColeopteraChrysomelidae

Kimoto, 1969

FAFFB9AC-DC8B-5015-B7EE-9F6095157D2E

**Figure 43.**
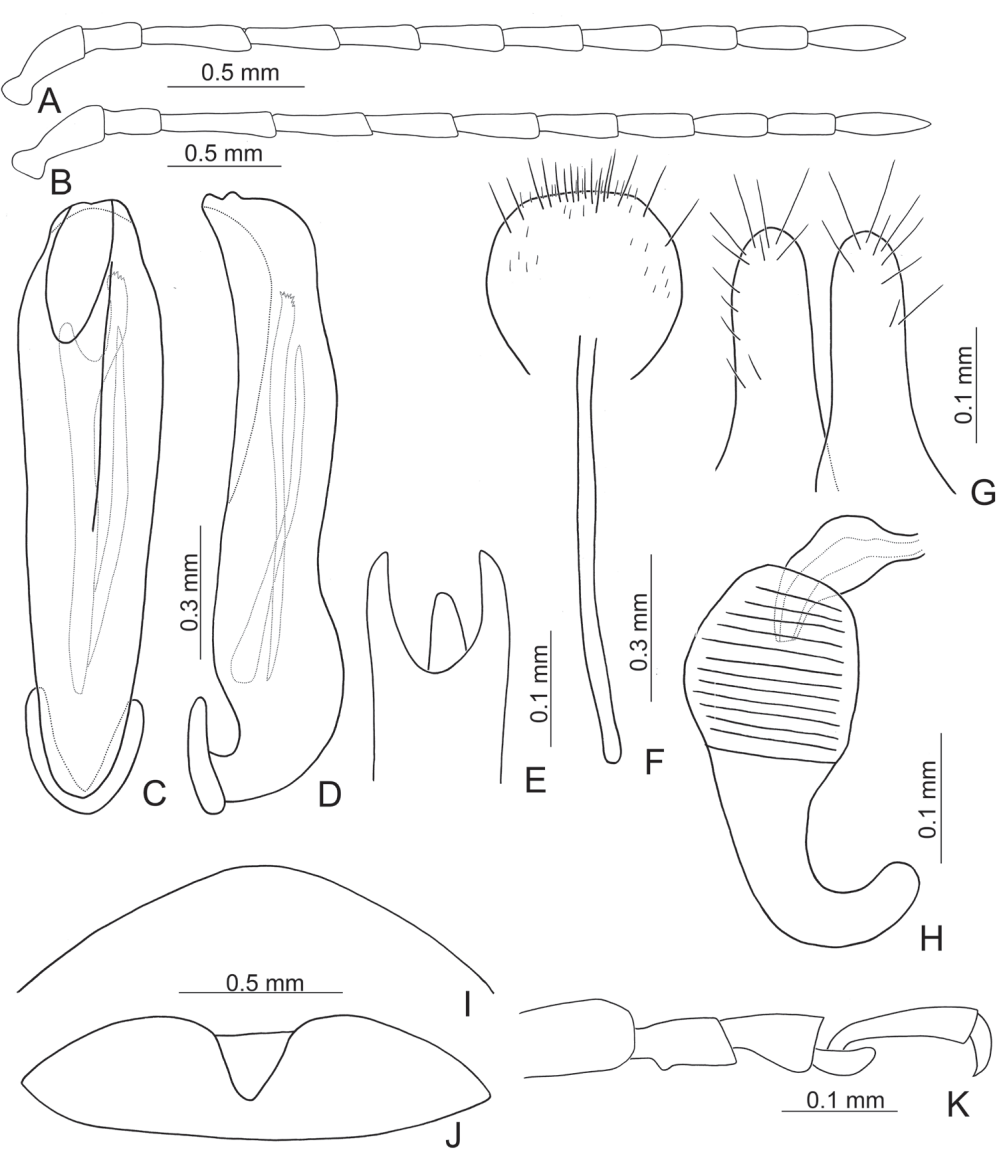
Diagnostic characters of *Pyrrhalta shirozui* Kimoto **A** antenna, male **B** antenna, female **C** aedeagus, dorsal view **D** ditto, lateral view **E** apex of tibia of middle leg, male **F** abdominal ventrite VIII **G** gonocoxae **H** spermatheca **I** abdominal ventrite V, female **J** abdominal ventrite V, male **K** tarsi of middle leg, male.

**Figure 44.**
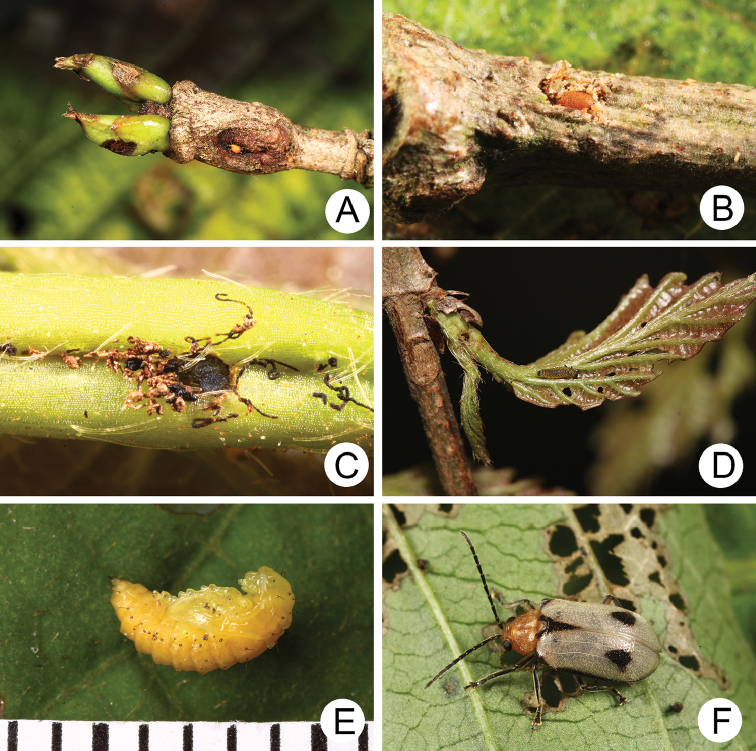
Field photographs of *Pyrrhalta shirozui* Kimoto on host plant **A** egg at crevice of small twig **B** egg at hole prepared by the female **C** Resting site (hole) prepared by larva **D** Larva **E** pupa **F** adult.

Figs 38D–I , 43 , 44

Pyrrhalta shirozui Kimoto, 1969: 26 (Taiwan); Kimoto and Chu 1996: 57 (catalogue); Kimoto and Takizawa 1997: 300 (key), 374; Beenen 2010: 453 (catalogue); Lee and Cheng 2010: 123 (redescription); Xue and Yang 2010: 129 (catalogue); Takahashi 2012: 324 (specimens at OMNH); Yang et al. 2015: 120 (catalogue).
Pyrrhalta (Pyrrhalta) shirozui : Wilcox 1971: 89 (catalogue).

#### Types.

***Holotype*** ♀ (KUEC, by original designation): “(Taiwan) / Sungkang [松崗] / Nantou Hsien [p, w] // 29.VI.[h] 1965 / T. Shirôzu [p, w] // Pyrrhalta / shirozui / Kimoto, n. sp. [h, w] // HOLOTYPE [p, r]”. ***Paratype*.** 1♂ (KMNH): “(TAIWAN) / Alishan [阿里山] / Chiai Hsien [p] / 29[h]. VII. 1966 / H. Kamiya leg. [p, w] // Pyrrhalta / shirozui / Kimoto, n. sp. [h, w] // PARATYPE [p, b]”.

#### Other material.

Taiwan. Chiayi: 1♂ (TARI), Yushan (玉山), 1.VII.2015, leg. J.-C. Chen; Hualien: 2♀ (TARI) Hahuan Cross-Ridge (合歡越嶺古道), 4.VIII.2018, leg. H.-F. Lu; 1♂ (TARI), Pilu (碧綠), 29.VI.2018, leg. H.-F. Lu; Ilan: 6♂, 3♀ (TARI), Mingchi (明池), 25.V.2008, leg. M.-H. Tsou; 4♂, 2♀ (TARI), same but with “16.VIII.2008”; 1♀ (TARI), Ssuyuan (思源), 11.VIII.2014, leg. J.-C. Chen; 1♂ (TARI), Taipingshan (太平山), 26–28.VII.1983, leg. L. Y. Chou; 1♂, 1♀ (TARI), same locality, 8.VII.2008, leg. H.-J. Chen; 3♂, 6♀ (TARI), same locality, 25.V.2009, leg. C.-F. Lee; 1♂ (TARI), Yingtzuling (鶯仔嶺), 3.VI.2011, leg. Y.-L. Lin; Nantou: 1♀ (TARI), Meifeng (梅峰), 5–9.X.1980, leg. C. C. Chen & C. C. Chien; 1♂, 1♀ (TARI), Nengkaoshan (能高山), 18.X.2011, leg. J.-C. Chen; 1♀ (TARI), Tatachia (塔塔加), 20.VII.2009, leg. S.-F. Yu; 1♀ (TARI), same but with “leg. H. Lee”; 1♀ (TARI), same but with “C.-F. Lee”; 1♂ (TARI), same locality, 21.IX.2009, leg. C.-F. Lee; 3♂♂, 2♀♀ (TARI), Tsuifeng (翠峰), 12–14.IX.1984, leg. K. S. Lin and S. C. Lin; Pingtung: 8♂♂ (TARI), Jinshuiying (浸水營), 12.VIII.2010, leg. J.-C. Chen; 1♀ (TARI), Tahanshan (大漢山), 1.VIII.2009, leg. U. Ong; 1♀ (TARI), same locality, 19.VII.2012, leg. C.-F. Lee; 1♂ (TARI), same locality, 29.VI.2018, leg. Y.-T. Chung; Taichung: 1♂ (TARI), Hassenzan (= Pahsienshan, 八仙山), 4.VI.1942, leg. A. Mutura; 1♂ (TARI), Wuwoweishan (屋我尾山), 5.VI.2012, leg. J.-C. Chen; Taipei: 2♂♂ (TARI), Fengkueitsui (風櫃嘴), 21.X.2007, leg. M.-H. Tsou; 6♂, 3♀ (TARI), Hsiaoyukeng (小油坑), 24.V.2008, leg. M.-H. Tsou; 4♀ (TARI), same but with “12.X.2008”; 9♀ (TARI), same locality and collector, reared from larvae, 21–29.III.2009; 1♀ (TARI), 5.XI.2006, Shihlin (士林), 5.XI.2006, leg. H.-T. Cheng; 2♂, 6♀ (TARI), Yangmingshan (陽明山), 12.V.2007, leg. M.-H. Tsou; 1♀ (TARI), same but with “27.V.2007”; Taitung: 3♂, 1♀ (TARI), Hsiangyang (向陽), 2.VII.2009, leg. S.-F. Yu; 1♀ (TARI), Liyuan (栗園), 19.VI.2013, leg. C.-F. Lee; 1♀ (TARI), Motien (摩天), 23.V.2011, leg. C.-F. Lee; Taoyuan: 4♂, 10♀ (TARI), Lalashan (拉拉山), reared form larvae, 27.IV.2009, leg. C.-F. Lee; 1♂, 9♀ (TARI), same but with “28.V.2009”; 1♀ (TARI), same locality, 15.VII.2009, leg. H.-J. Chen; 1♀ (TARI), Tamanshan (塔曼山), 25.VIII.2008, leg. H. Lee.

#### Redescription.

Length 4.9–6.8 mm, width 2.4–3.4 mm. Body color (Fig. 38D–F) yellowish brown; antennae blackish brown but inner sides of five basal antennomeres yellowish brown; slender black stripe along outer and basal margins of elytra, extending into humeral calli, surrounding scutellum and suture, abbreviated at basal 1/3 or middle, with one additional pair of large black spots inside middle of apical 1/3; legs black, but inner sides of femora and tibiae yellowish brown. Eyes small, interocular space 2.06–2.26 × diameter of eye. Antennae filiform in males (Fig. 43A), length ratios of antennomeres I–XI 1.0: 0.6: 1.1: 0.9: 0.9: 0.9: 0.8: 0.8: 0.8: 0.7: 1.0, length to width ratios of antennomeres I–XI 2.9: 2.2: 4.2: 3.7: 3.4: 3.4: 3.2: 3.1: 3.1: 2.9: 3.9; filiform in females (Fig. 43B), length ratios of antennomeres I–XI 1.0: 0.6: 1.1: 0.9: 0.9: 0.8: 0.8: 0.7: 0.7: 0.7: 0.9, length to width ratios of antennomeres I–XI 2.7: 2.1: 4.4: 3.8: 3.5: 3.3: 3.1: 3.3: 3.2: 3.2: 4.8. Pronotum and elytra convex. Pronotum 2.0 × wider than long, disc smooth; with coarse, dense punctures, and short pubescence; with median longitudinal and lateral depressions; lateral margins rounded, widest at apical 1/3, basal margin truncate, apical margin slightly concave; anterior and posterior setiferous punctures strongly erect. Elytra elongate and broad, parallel-sided, 1.5 × longer than wide; disc smooth, with extremely coarse, dense punctures, and short pubescence. Apical spur of tibia of middle leg small (Fig. 43E), and tarsomere I of middle leg axe-shaped in lateral view, with narrow basal half and expanded apical half in males (Fig. 43K). Aedeagus (Fig. 43C, D) wide in dorsal view, 4.5 × longer than wide, apex asymmetrical, widest at apical 1/6, gradually narrowed toward base, apex rounded but depressed at middle; strongly curved near base in lateral view, slightly and apically curved, apex truncate with a rounded process on left; ostium not covered by membrane; two endophallic sclerites elongate, apex of primary endophallic sclerite with several teeth, 0.6 × as long as aedeagus, with one short branch at apical 1/5, secondary sclerite slightly shorter, 0.8 × as long as the primary endophallic sclerite, apex acute. Only apices of gonocoxae (Fig. 43G) sclerotized and longitudinal, with a number of long setae along lateral and apical margins. Ventrite VIII (Fig. 43F) narrow; disc with several long setae and short setae along apical margin; spiculum long. Receptacle of spermatheca (Fig. 43H) slightly swollen; pump short and strongly curved; sclerotized proximal spermathecal duct wide and short. Apical margin of abdominal ventrite V slightly concave, with deeply rounded depression at middle in males (Fig. 43J); broadly rounded in females (Fig. 43I).

#### Variation.

Some specimens have a black stripe along the entire suture of the elytra (Fig. 38G); some have the black spot separated into two, sometimes connected (Fig. 38H); some have broad black stripe along suture, expanding laterally to connect with black spot (Fig. 38I); many specimens are intermediate between these color patterns.

#### Remarks.

adults of *P. shirozui* Kimoto are easily recognized by the characteristic color patterns on the elytra and sparse, coarse elytral punctures, as well as diagnostic shape of the aedeagus differing from all other species of *Pyrrhalta*.

#### Host plants.

Larvae and adults feed on leaves of *Viburnum formosanum* (Hance) Hayata, V. foetidum Wall. var. rectangulatum Rehder, *V. integrifolium* Hayata, *V. luzonicum* Rolfe, *V. taitoense* Hayata, and *V. urceolatum* Siebold and Zucc.

#### Biology.

The following life cycle information is based on Mr Mei-Hua Tsou’s (TCRT) observations (Lee and Cheng 2010). Females deposited single eggs in crevices of small twigs (Fig. 44A) or a hole prepared by the female (Fig. 44B) during autumn. The larvae hatched when plants sprouted during spring. They prepared a hole as a resting site (Fig. 44C). They exited the hole only when feeding on leaves (Fig. 44D). Larval duration was 14 days. mature larvae burrowed into soil and built underground chambers for pupation. Duration of the pupal stage (Fig. 44E) was 22–28 days. Newly emerged adults appeared during spring and were active (Fig. 44F) during summer and autumn.

#### Distribution.

This species is widespread in lowlands (0–1,500 m) in northern Taiwan and mid-altitudes (1,500–2,500 m) in central Taiwan.

### *Pyrrhalta* species currently unassigned to any species group

#### Pyrrhalta kobayashii

Taxon classificationAnimaliaColeopteraChrysomelidae

Kimoto, 1974

D90575C1-E61E-5414-A704-54133B1C7C97

Figs 45A–C , 46

Pyrrhalta kobayashii Kimoto, 1974: 25; Kimoto and Chu 1996: 56 (catalogue); Kimoto and Takizawa 1997: 301 (key), 373; Beenen 2010: 453 (catalogue); Xue and Yang 2010: 124 (catalogue); Yang et al. 2015: 117 (catalogue).

##### Types.

***Holotype*** ♀ (OMNH): “Mt. ALI / FORMOSA / 22.VII.1970 / T. KOBAYASHI [p, y] // Pyrrhalta / kobayashii / Kimoto [h, w] // HOLOTYPE [p, r]. ***Paratype*.** 1♀ (KMNH): “(TAIWAN) / Alishan (阿里山) / Chiai Hsien [p] / 29[h]. VII. 1966 / H. Kamiya leg. [p, w] // Pyrrhalta / kobayashii / Kimoto [h, w] // PARATYPE [p, b]”.

##### Other material.

Taiwan. Nantou: 1♀ (TARI), Huakang (華岡), 20.VII.2017, leg. J.-C. Chen; Taichung: 1♀ (TARI), Pilu (畢祿), 2.VII.2008, leg. M.-H. Tsou; Taitung: 1♀ (TARI), Hsiangyang (向陽), 12.VII.2012, leg. J.-C. Chen.

##### Redescription

**Figure 45.**
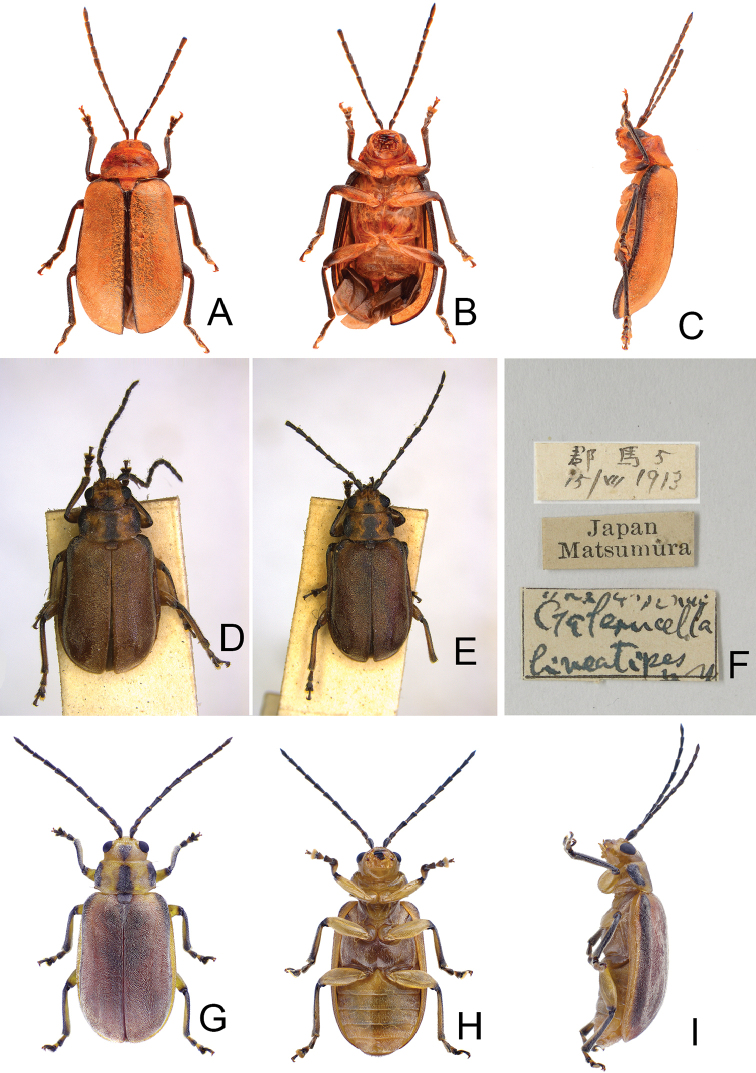
Habitus of *Pyrrhalta kobayashii* Kimoto and *P. lineatipes* (Takei) **A***P. kobayashii*, female, dorsal view **B** ditto, ventral view **C** ditto, lateral view **D***P. lineatipes*, lectotype, dorsal view **E***P. lineatipes*, paralectotype, dorsal view **F***P. lineatipes*, type labels **G***P. humeralis*, from Taiwan, female, dorsal view **H** ditto, ventral view **I** ditto, lateral view.

**Figure 46.**
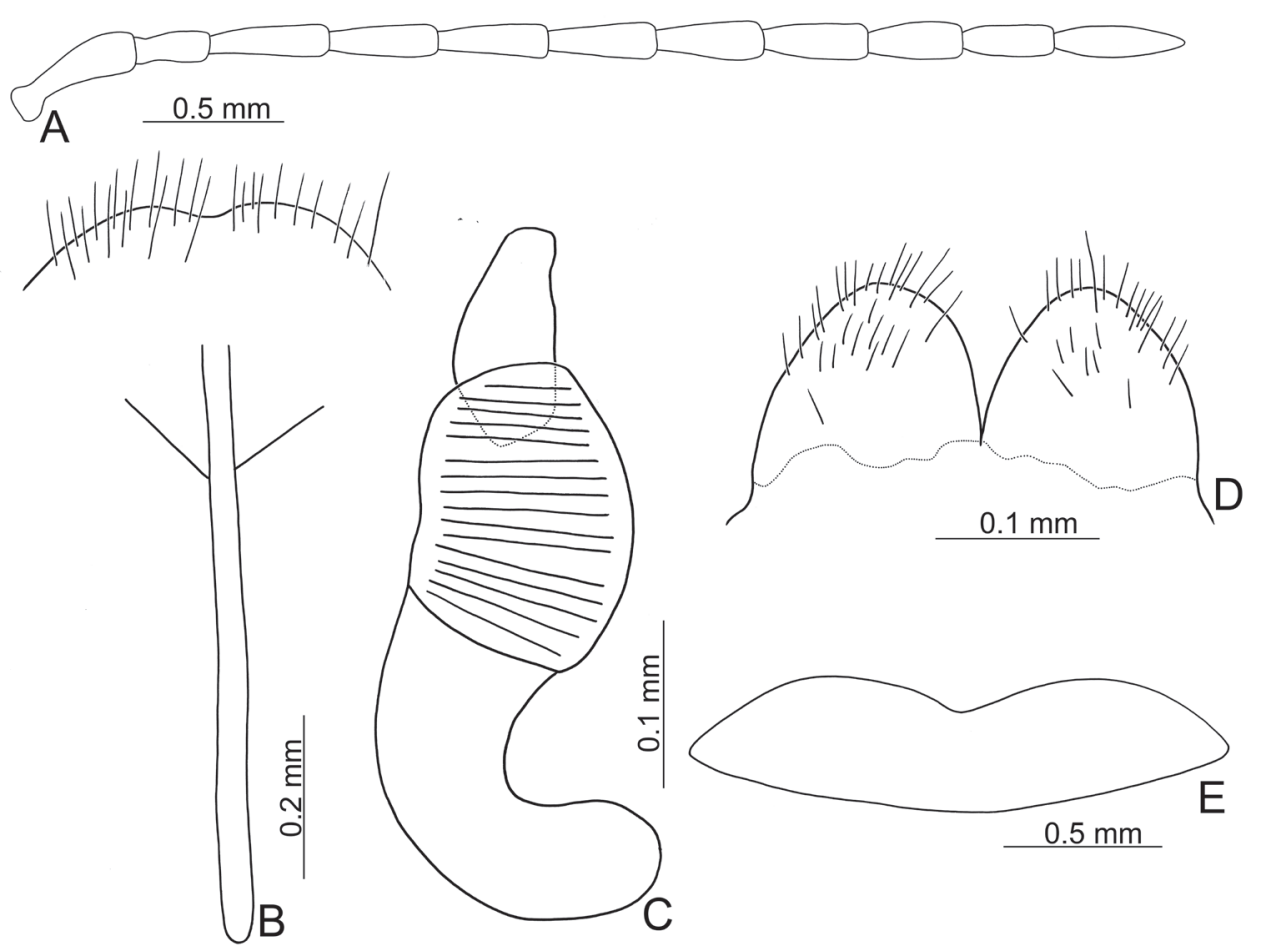
Diagnostic characters of *Pyrrhalta kobayashii* Kimoto, female **A** antenna **B** abdominal ventrite VIII **C** spermatheca **D** gonocoxae **E** abdominal ventrite V.

**(females).** Length 6.2–6.3 mm, width 3.2 mm. Body yellow (Fig. 45A–C); but antennae, lateral margins of elytra (sutures, basal and lateral margins), tibiae, and tarsi black; apices of femora darker. Eyes small, interocular space 2.05–2.09 × diameter of eye. Antennae filiform (Fig. 46A), length ratios of antennomeres I–XI 1.0: 0.5: 0.9: 0.8: 0.8: 0.8: 0.8: 0.8: 0.7: 0.7: 1.0, length to width ratios of antennomeres I–XI 3.2: 2.2: 3.6: 3.3: 3.2: 3.0: 2.9: 2.8: 2.5: 2.9: 4.1. Pronotum and elytra convex. Pronotum 1.8–2.0 × wider than long, with transverse ridge along apical margin deflexed at antero-lateral angles; disc smooth, with coarse punctures laterally, smaller medially; with dense short pubescence, but reduced above ridge, with median longitudinal and lateral depressions; lateral margins moderately rounded, widest at middle, apical and basal margins slightly concave; anterior and posterior setiferous punctures not erect. Elytra broad, parallel-sided, 1.5–1.6 × longer than wide; disc smooth, with dense, fine punctures, and dense, short pubescence, one pubescent seta in each puncture. Gonocoxae (Fig. 46D) apically sclerotized, small, broadly rounded, disc and apical margin with short dense setae. Ventrite VIII (Fig. 46B) transverse, with apical margin depressed at middle, a number of long setae near apical margin, spiculum long. Receptacle of spermatheca (Fig. 46C) slightly swollen and elongate; pump short and strongly curved; sclerotized proximal spermathecal duct narrow and short. Apical margin of abdominal ventrite V moderately concave medially (Fig. 46E).

##### Remarks.

The color pattern of adults of *P. kobayashii* (Fig. 45A) is similar to that of the typical form of *P. discalis* Gressitt & Kimoto (Fig. 31A). The species differs by the more slender antennae, antennomeres IV–X 2.8–3.6 × longer than wide (Fig. 46A) (antennomeres IV–X 1.7–2.4 × longer than wide in *P. discalis* (Fig. 32B)) and relatively narrow elytra, 1.6 × longer than wide (elytra 1.4 × longer than wide in *P. discalis*).

##### Food plant.

Unknown.

##### Distribution.

The species occurs at scattered localities at mid-altitudes (1,500–2,500 m) in central and southern Taiwan.

#### Pyrrhalta lineatipes

Taxon classificationAnimaliaColeopteraChrysomelidae

(Takei, 1916), resurrected

223C713D-BB25-53F6-B663-6E6ABB32E13D

**Figure 47.**
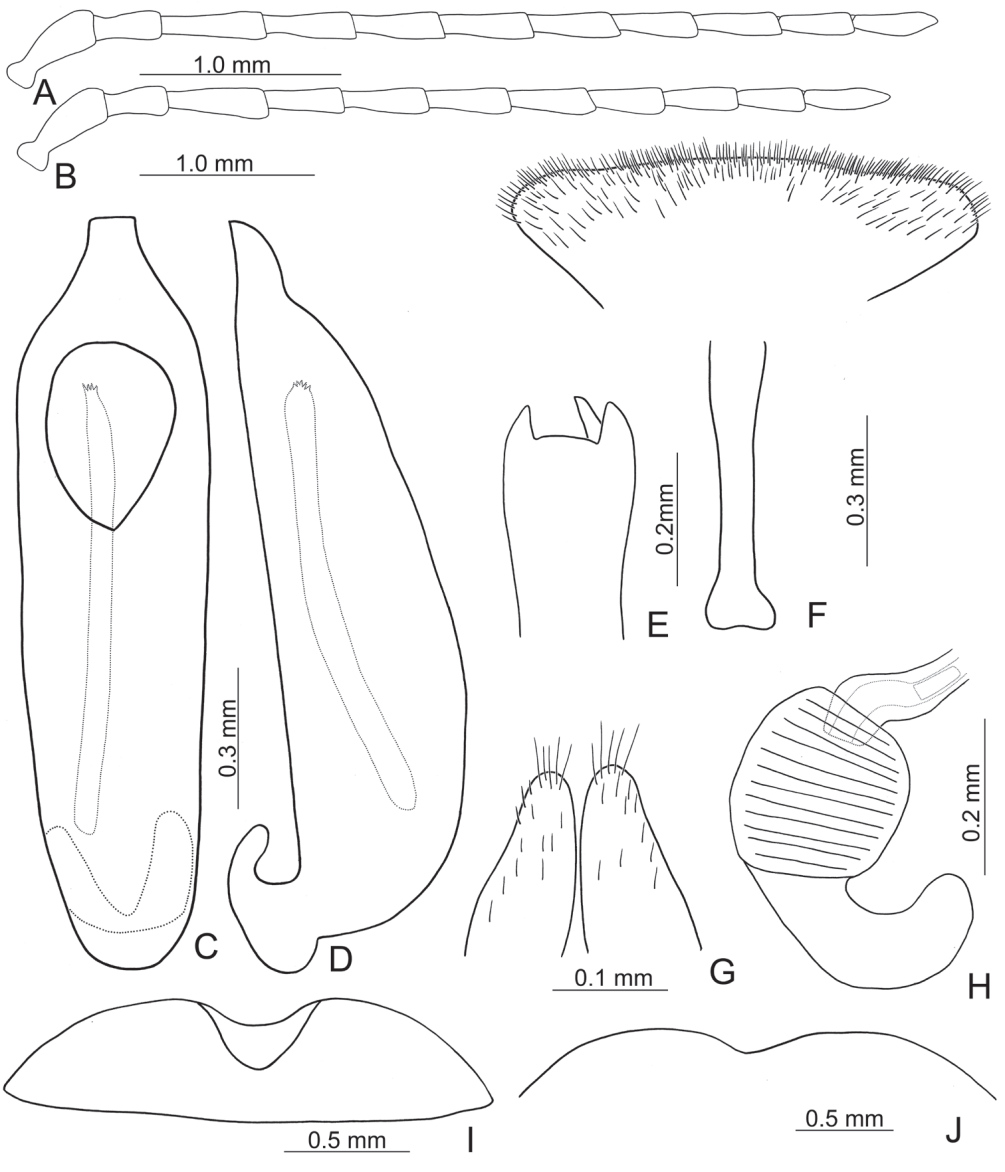
Diagnostic characters of *Pyrrhalta lineatipes* (Takei) **A** antenna, male **B** antenna, female **C** aedeagus, dorsal view **D** ditto, lateral view **E** apex of tibia of middle leg, male **F** abdominal ventrite VIII **G** gonocoxae **H** spermatheca **I** abdominal ventrite V, male **J** abdominal ventrite V, female.

**Figure 48.**
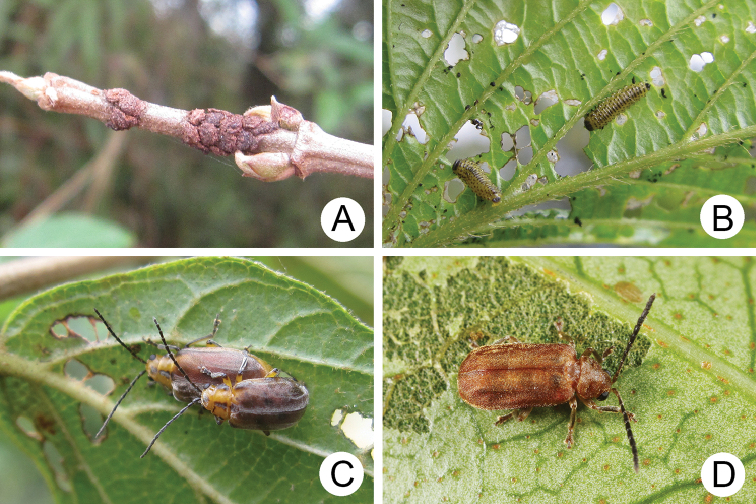
Field photographs of *Pyrrhalta lineatipes* (Takei) and *P. ohbayashii* Kimoto on host plant **A***P. lineatipes*, egg masses **B** same, third-instar larvae **C** same, adults **D***P. ohbayashii*, adult.

Figs 45G–I , 47 , 48A–C

Galerucella lineatipes Takei, 1916: 35 (Japan: Gumma).
Galerucella humeralis Chen, 1942: 17 (China: Guanxi, Liaoning). syn. nov.
Pyrrhalta humeralis : Nakane & Kimoto, 1961: 21 (Japan: Okinawa island); Gressitt and Kimoto 1963: 451 (China: Anhui, Hubei, Fujian, Guandong, Sichuan); Kimoto 1964a: 301 (Japan: Hokkaido, Honshu, Shikoku, Kyushu); Kimoto and Gressitt 1966: 477 (key), 520 (Ryukyus); Kimoto 1969: 28 (Taiwan); Kimoto and Hiura 1971: 15 (Japan); Kimoto 1985: 4 (catalogue); Lee 1990: 81 (larval description, Japan); Jiang 1992: 647 (China: Sichuan); Li 1992: 185 (China: Liaoning); Yang 1993: 332 (China: Hubei); Kimoto and Takizawa 1994: 234 (key), 306 (Japan); Kimoto and Chu 1996: 55 (catalogue); Kimoto and Takizawa 1997: 300 (key), 373; Yang et al. 1997: 865 (China: Sichuan); Wang and Yang 1998: 65 (China: Fujian); Lee and An 2001: 119 (South Korea); Mikhailov and Hayashi 2002: 34 (Sakhalin); Yang 2002: 627 (China: Fujian); Park and Lee 2004: 229 (larval description, Korea); Lee and Ho 2006: 82 (host plants); Wang and Yang 2006: 112 (China: Gansu); Beenen 2010: 452 (catalogue); Xue and Yang 2010: 123 (catalogue); Takahashi 2012: 323; Yang et al. 2015: 117 (China: Helongjiang, Jiangxi, Jilin, Gansu, Shaanxi, Zhejiang, Hunan, Guanxi); Matsumura et al. 2017: 85 (female reproductive system); Cho and An 2020: 22 (catalogue, South Korea).
Pyrrhalta (Pyrrhalta) humeralis : Wilcox 1971: 86 (catalogue).

##### Types.

*Gallerucella lineatipes*. ***Lectotype*** ♂ (SEHU) (Fig. 45A, C), here designaed: “Japan / Matsumura [p, w] // 群馬 [= Gumma] 5 / 15/VII 1913 [h, on the back of the same card] // Galerucella / lineatipes / n. sp. [h, w]”. ***Paralectotype*.** 1♂ (SEHU) (Fig. 45B), same data as holotype. Both specimens glued on separated cards but pined with the same pine originally. Now both are separated and the paratype mounted with copies of the labels.

*Galerucella humeralis*. Presumably deposited at the IZAS based on the original description (Chen 1942). However, the type seems to be lost (Ruie Nie, pers. comm., 26 Nov 2018).

##### Other material.

China. Fujian: 1♀ (CAS), Shaowu, Tachulan, 14.VII.1946, leg. T. C. Maa; Guangdong: 1♂ (CAS), Taiyong, 5.VIII.1936, leg. K, Gressitt, det. Gressitt and Kimoto, 1961; Heilongjiang: 1♀ (TARI), Dailing (岱岭), 23.VII.1958, leg. S. X. Zhou; Hubei: 1♀ (KMNH), Leong-Ho-Kow to Wang-Ga-Ying, 18.IX.1948, leg. Gressitt & Djou; Japan. Honshu: 1♀ (TARI), Nagano-Ken, Noziri, 10.VIII.1940, leg. T. Nakane; 2♀ (TARI), Yamaguchi, Tokusa, 16.VII.1922, leg. T. Shiraki; Kyushu: 1♂ (TARI), Mt. Korasan (Chikugo), 8.VIII.1934, leg. K. Yamauchi; Sikoku: 2♂, 2♀ (TARI), Kochi-Ken, 7.XI.1935, leg. I. Okubo; Ryukyu Islands: 1♂, 1♀ (CAS), 1♂ (NHMUK), Okinawa I., Nakijin, 26.IV.1964, leg. T. Takara; South Korea. 2♀ (TARI), Suigen, 11.VIII.1936, leg. K. Saito; Taiwan. Hualien: 4♂, 1♀ (TARI), Liyutan (鯉魚潭), 27.VIII.2016, leg. H.-F. Lu; 6♂, 10♀ (TARI), same but with “17.IV.2017”; Nantou: 1♀ (TARI), Meifeng (梅峰), 5–9. X.1980, leg. C. C. Chen & C. C. Chien; Taichung: 2♂ (TARI), Wuleng (武陵), 25.VII.2010, leg. S.-F. Yu; 2♂, 1♀ (TARI), same locality, 13.IX.2010, leg. M.-H. Tsou; 6♂, 9♀ (TARI), same locality, 6.XI.2016, leg. J.-C. Chen.

##### Redescription.

Length 6.0–7.9 mm, width 2.9–4.1 mm. Body color (Fig. 45G–I) yellowish brown; vertex with one longitudinal black spot at middle, antennae blackish brown; pronotum with three large black spots, one spot at center, elongate, extending from near apex to near base; two wide spots along lateral margins; scutellum dark brown or blackish brown; elytra with longitudinal black stripe from humerus to middle; legs yellowish brown, but apices of femora, outer sides of tibiae, and apical 2/3 of tarsi black. Eyes relatively small, interocular space 2.88–2.91 × diameter of eye. Antennae filiform in males (Fig. 47A), length ratios of antennomeres I–XI 1.0: 0.7: 1.1: 0.9: 0.9: 0.9: 0.9: 0.9: 0.9: 0.7: 0.9, length to width ratios of antennomeres I–XI 2.5: 2.4: 3.7: 3.2: 3.2: 3.4: 3.4: 3.4: 3.5: 3.1: 3.7; similar in females (Fig. 47B), length ratios of antennomeres I–XI 1.0: 0.6: 1.0: 0.8: 0.8: 0.8: 0.8: 0.7: 0.7: 0.7: 0.9, length to width ratios of antennomeres I–XI 2.7: 2.1: 3.3: 3.1: 3.2: 3.1: 3.1: 3.1: 3.2: 3.0: 3.8. Pronotum and elytra moderately convex. Pronotum 2.1–2.3 × wider than long, disc with transverse ridge along apical margin deflexed at antero-lateral angles, with dense, extremely coarse punctures, and long pubescence, punctures reduced on ridge; with median longitudinal and lateral depressions; lateral margins medially broadened, apical margin slightly concave, basal margin straight. Elytra elongate, parallel-sided, 1.5–1.6 × longer than wide; disc rough, with sparse fine punctures, and long, extremely dense pubescence. Apical spur of tibia of middle leg small (Fig. 47E), tarsomere I of middle leg not modified in males. Aedeagus (Fig. 47C, D) broad in dorsal view, 4.5 × longer than wide, sides slightly asymmetric, strongly broadened from apex to apical 1/10, slightly narrowed towards base, apex truncate; strongly curved at base in lateral view, moderately broadened from apex to basal 2/5, apex acute; ostium not covered by membrane; single endophallic sclerite long, 0.5 × as long as aedeagus, with several apical small teeth. Gonocoxae (Fig. 47G) longitudinal, base membranous, disc with sparse, short setae, several long setae along apical margin. Ventrite VIII (Fig. 47F) extremely transverse; disc with extremely dense, short setae along apical area; spiculum short. Receptacle of spermatheca (Fig. 47H) very swollen; pump short and strongly curved; sclerotized proximal spermathecal duct wide and short. Apical margin of abdominal ventrite V with rounded depression at middle, followed by shallow notch in males (Fig. 47I); only with shallow depression in females (Fig. 47J).

##### Remarks.

Adults of *P. lineatipes* (Takei) (Fig. 45G), *X. aenescens* (Fairmaire) (Fig. 1D), and *P. jungchani* sp. nov. (Fig. 38A) are easily recognized by the three black spots on the pronota. This species (Fig. 45I) is most similar to *P. jungchani* sp. nov. (Fig. 38C) based on the brown elytra with black stripes arising from humeral calli and convex pronotum and elytra (entirely metallic green elytra and dorso-ventral flattened pronotum and elytra in *X. aenescens* (Fig. 1F)). It differs from *P. jungchani* sp. nov. by the more dense pubescence, sparse punctures on elytra (sparse pubescence and extremely dense punctures on elytra in *P. jungchani* sp. nov.), and normal tarsomere I of middle leg in males (Fig. 39H) (modified tarsomere I of middle leg in males of *P. jungchani* sp. nov. (Fig. 39H)). In addition, the aedeagus (Fig. 47C, D) and abdominal ventrite VIII in females (Fig. 47F) are diagnostic.

Mr. Takei sent specimens to Dr. Matsumura for identification. He wrote a new species name on the identification card, *Galerucella lineatipes* sp. n., but that name was never published. Later, Takei (1916) described this new species collected by him as *Galerucella lineatipes* Mats. (n. sp.). Thus, the correct authorship is Takei. Two types at the SEHU fit the original description well; it is a distinct species that differs from *Galerucella calmariensis* and is regarded as a senior synonym of *P. humeralis*.

Although *Pyrrhalta lineatipes* feed on leaves of *Viburnum* spp., it does not belong to the *P. shirozui* species group due to a number of apomorphies in adults and arrangement of eggs. *Pyrrhalta lineatipes* differs from members of the *P. shirozui* species group with its symmetrical aedeagus (Fig. 47C) lacking a secondary endophallic sclerite (asymmetrical aedeagi (Figs 39C, 42C, 43C) and with the second endophallic sclerite in *P. shirozui* species group), the extremely transverse ventrite VIII in females, and with short speculum (Fig. 47F) (vs. narrow ventrite VIII in females and with long speculum in *P. shirozui* species group (Figs 39E, 42E, 43F), and egg mass on small twigs (Fig. 48A) (the single egg on small twigs in *P. shirozui* (Fig. 44A, B). Interestingly, females of *P. viburni* also deposited egg masses (Hilker 1992) on small twigs as those of *P. lineatipes*, and larvae and adults fed on leaves of *Viburnum* spp., so both might belong to the same species-group.

##### Host plants.

*Viburnum* sp. (Gressitt and Kimoto 1963), *V. odoratissimum* Ker. in Japan (Lee 1990), *V. sargentii* Koehne in the laboratory, Korea (Park and Lee 2004), *V. betulifolium* Batalin (present study), *V. parvifolium* Hayata (present study), *V. taitoense* Hayata (present study), *V. dilatatum* Thunb, *V. awabuki* Koch, *V. opulus*, *V. phlebotrichum*, *V. sieboldii* (Lee and Cho 2006), *Salix* sp. (Gressitt and Kimoto 1963; Lee and Cho 2006; need further confirmation).

##### Biology.

The overwintering eggs of *P. lineatipes* were deposited into the twigs of the hostplants (Fig. 48A), *Viburnum* sp., as observed by Mr. His-Feng Lu, 15 November 2016, in Liyutan, eastern Taiwan. Each egg mass was covered with feces and small fragments of chewed plant material. young larvae were found on 5 March of the following year. They were transferred to the laboratory for rearing and fed on leaves. mature larvae (Fig. 48B) burrowed into soil and built underground chambers for pupation. The newly emerged adults crawled out soil (Fig. 48C) April 7.

##### Distribution.

China (Anhui, Fujian, Gansu, Guandong, Guanxi, Helongjiang, Hubei, Hunan, Jiangxi, Jilin, Liaoning, Shaanxi, Sichuan, Zhejian; Yang et al. 2015), Japan (Hokkaido, Honshu, Shikoku, Kyushu; Okinawa island), Korea, Taiwan. It is only found in a few localities from lowlands to mid-altitudes in eastern Taiwan.

#### Pyrrhalta ohbayashii

Taxon classificationAnimaliaColeopteraChrysomelidae

Kimoto, 1984

E683AB8A-DAD2-5F90-B986-F980A1E57BEE

**Figure 49.**
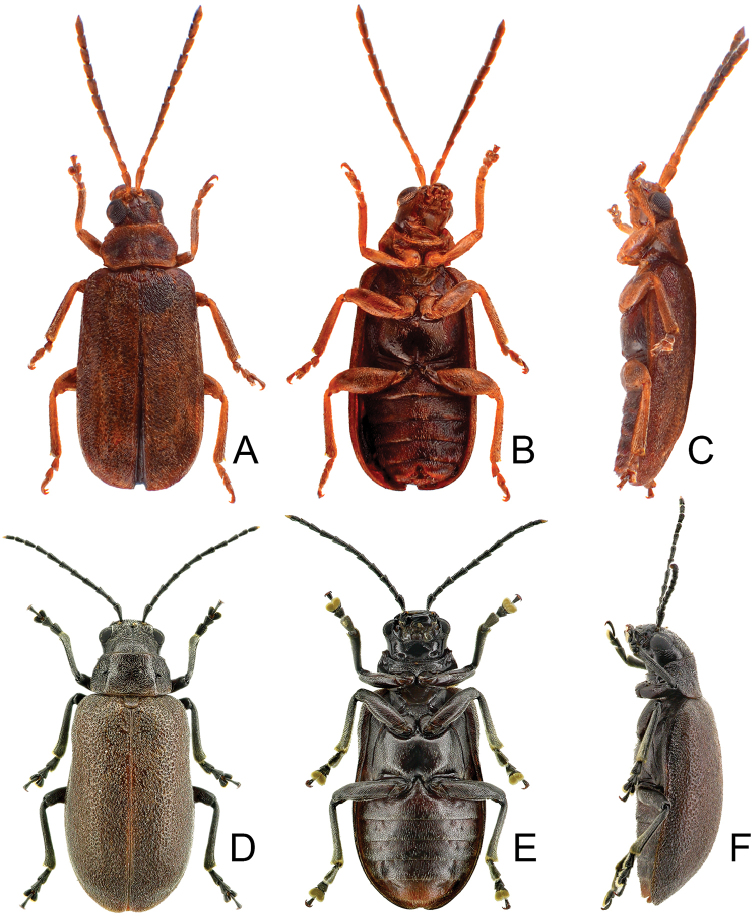
Habitus of *Pyrrhalta ohbayashii* Kimoto and *P. takizawai* Kimoto **A***P. ohbayashii*, male, dorsal view **B** ditto, ventral view **C** ditto, lateral view **D***P. takizawai*, female, dorsal view **E** ditto, ventral view **F** ditto, lateral view.

**Figure 50.**
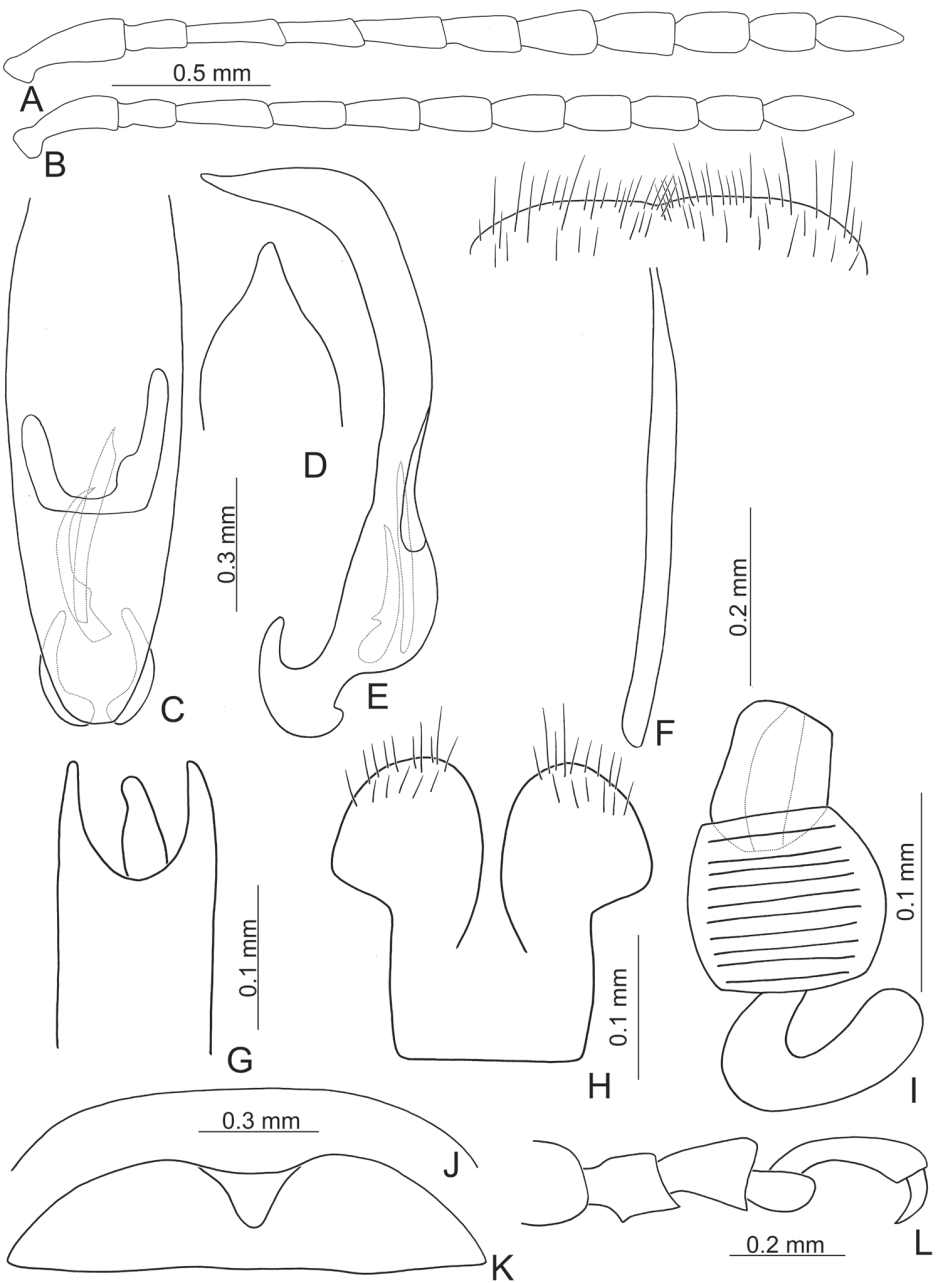
Diagnostic characters of *Pyrrhalta ohbayashii* Kimoto **A** antenna, male **B** antenna, female **C** aedeagus except apex, dorsal view **D** apex of aedeagus, dorsal view **E** aedeagus, lateral view **F** abdominal ventrite VIII **G** apex of tibia of middle leg, male **H** gonocoxae **I** spermatheca **J** abdominal ventrite V, female **K** abdominal ventrite V, male **L** tarsi of middle leg, male.

Figs 48D , 49A–C , 50

Pyrrhalta ohbayashii Kimoto, 1984: 46; Kimoto 1987: 188 (additional records); Kimoto 1991: 9 (additional records); Kimoto and Chu 1996: 57 (catalogue); Kimoto and Takizawa 1997: 300 (key), 373; Beenen,2010: 453 (catalogue); Xue and Yang 2010: 127 (catalogue); Yang et al. 2015: 119 (catalogue).

##### Types.

***Holotype*** ♀ (KUEC, by original designation): “(FORMOSA) / Mt. Lala-shan [拉拉山] / Taoyuan Hsien / 7, V 1982 / N. Ohbayashi leg. [p, w] // Pyrrhalta / ohbayashii / Kimoto, n. sp. [h, w] // HOLOTYPE [p, r] // KU. Type / No. 2438 [p, w]”. ***Paratype*.** 1♀ (KMNH): “(FORMOSA) / Mt. Lala-shan [拉拉山] / Taoyuan Hsien / 7, V 1982 / N. Ohbayashi leg. [p, w] // Pyrrhalta / ohbayashii / Kimoto, n. sp. [h, w] // PARATOPOTYPE [p, b]”.

##### Other material.

Taiwan. Kaohsiung: 1♀ (KMNH), Tayuenshan, near Liukui (六龜), 5.VI.1989, leg. K. Baba, det. S. Kimoto, 1990; 1♂ (TARI), Tengchih (藤枝), 10.VIII.2013, leg. W.-C. Liao; 1♂ (TARI), same locality, 8.V.2020, leg. Y.-C. Hsu; 1♀ (TARI), Tona trail (多納林道), 5.XI.2016, leg. W.-C. Liao; Pingtung: 1♀ (TARI), Peitawushan (北大武山), 28.V.2014, leg. Y.-T. Chung; 4♀ (TARI), same but with “1.IX.2016”; 1♂, 1♀ (TARI), same but with “30.IV.2017”; 2♀ (TARI), same but with “28.IX.2017”; 3♂, 1♀ (TARI), Shahsi trail (沙溪林道), 20.VII.2017, leg. B.-X. Guo; 5♂, 7♀ (TARI), Taiwu (泰武), 11.IX.2020, leg. Y.-T. Chung; Taipei: 3♂ (TARI), Yingzuling (鶯子嶺), 24.VII.2010, leg. Y.-L. Lin.

##### Redescription.

Length 4.5–4.6 mm, width 1.9–2.1 mm. Body color (Fig. 49A–C) dark brown; antennae black except three basal antennomeres. Eyes large, interocular space 1.77–1.91 × diameter of eye. Antennae filiform in males (Fig. 50A), gradually broadened from antennomere IV, broadest at VII and VIII, then gradually narrowed to apical antennomere, length ratios of antennomeres I–XI 1.0: 0.5: 0.8: 0.7: 0.7: 0.6: 0.6: 0.6: 0.6: 0.6: 0.7, length to width ratios of antennomeres I–XI 3.0: 2.2: 3.5: 3.0: 2.6: 2.0: 1.8: 1.9: 1.8: 1.7: 2.5; similar in females (Fig. 50B), length ratios of antennomeres I–XI 1.0: 0.5: 0.9: 0.7: 0.7: 0.7: 0.7: 0.6: 0.6: 0.6: 0.8, length to width ratios of antennomeres I–XI 3.3: 2.0: 3.5: 2.8: 2.4: 2.1: 2.0: 1.7: 1.9: 1.9: 2.6. Pronotum and elytra convex. Pronotum 1.8–1.9 × wider than long, disc with reticulate microsculpture; with extremely dense, coarse punctures, and short pubescence, with median longitudinal and lateral depressions; lateral margins slightly rounded, widest at middle, apical and basal margin slightly concave; anterior and posterior setiferous punctures slightly erect. Elytra elongate, parallel-sided, 1.7–1.8 × longer than wide; disc with reticulate microsculpture, with sparse, coarse punctures, and short pubescence. Apical spur of tibia of middle leg small (Fig. 50G), and tarsomere I of middle with narrow basal half and small acute process at basal 1/3 in lateral view in males (Fig. 50L). Aedeagus (Fig. 50C–E) broad in dorsal view, 4.2 × longer than wide, broadest at middle, symmetric, apex lanceolate; ostium transverse at apical 1/3, not covered by a membrane; strongly curved near base and at apical 1/5 in lateral view, apex narrowly rounded; two endophallic sclerites small and elongate, primary sclerite straight in lateral view, 0.3 × as long as aedeagus, secondary sclerite curved in lateral view, 0.7 × as long as primary sclerite. Gonocoxae (Fig. 50H) basally connected, short, with a number of long setae near apex. Ventrite VIII (Fig. 50F) with apical area well sclerotized, apical margin truncate but slightly concave at middle, with dense, long setae along apical area, spiculum extremely long. Receptacle of spermatheca (Fig. 50I) very swollen; pump long and strongly curved; sclerotized proximal spermathecal duct wide and short. Apical margin of abdominal ventrite V slightly concave medially and with deep depression in males (Fig. 50K); straight in females (Fig. 50J).

##### Remarks.

Adults of *P. ohbayashii* Kimoto (Fig. 49C) are similar to those of *P. ishiharai* Kimoto (Fig. 34A) and *P. wulaiensis* sp. nov. (Fig. 34D) in possessing longitudinal ridges on the brown elytra, but differ by the narrower elytra, 1.7–1.8 × longer than wide (elytra 1.5 × longer than wide in *P. ishiharai* and *P. wulaiensis* sp. nov.). Gonocoxae are similar to those species of *Xanthogaleruca*. In males of *P. ohbayashii*, the aedeagus is diagnostic; strongly curved at apical 1/3 and the extremely small endophallic sclerites.

##### Food plant.

Adults feed on leaves of Prunus phaeosticta var. phaeosticta (Hance) Maxim. (Fig. 48D).

##### Distribution.

The species is widespread at lowlands (0–1,500 m) in northern and southern Taiwan.

#### Pyrrhalta takizawai

Taxon classificationAnimaliaColeopteraChrysomelidae

Kimoto, 1996

DEDB15F9-C017-5291-9F5B-96D3951FC335

**Figure 51.**
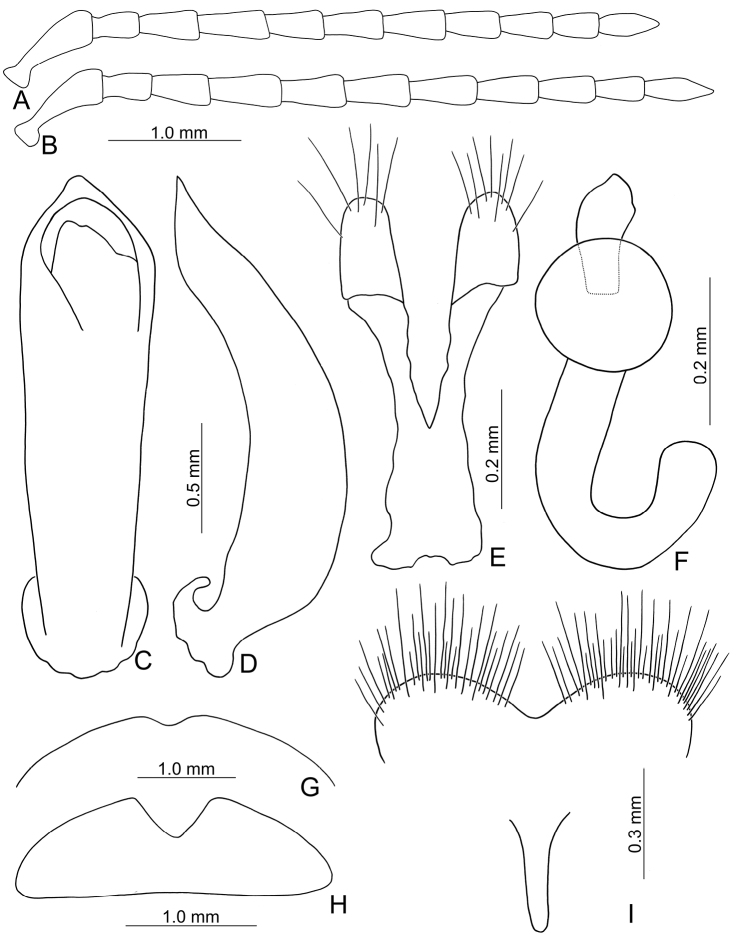
Diagnostic characters of *Pyrrhalta takizawai* Kimoto **A** antenna, male **B** antenna, female **C** aedeagus, dorsal view **D** ditto, lateral view **E** gonocoxae **F** spermatheca **G** abdominal ventrite V, female **H** abdominal ventrite V, male **I** abdominal ventrite VIII.

**Figure 52.**
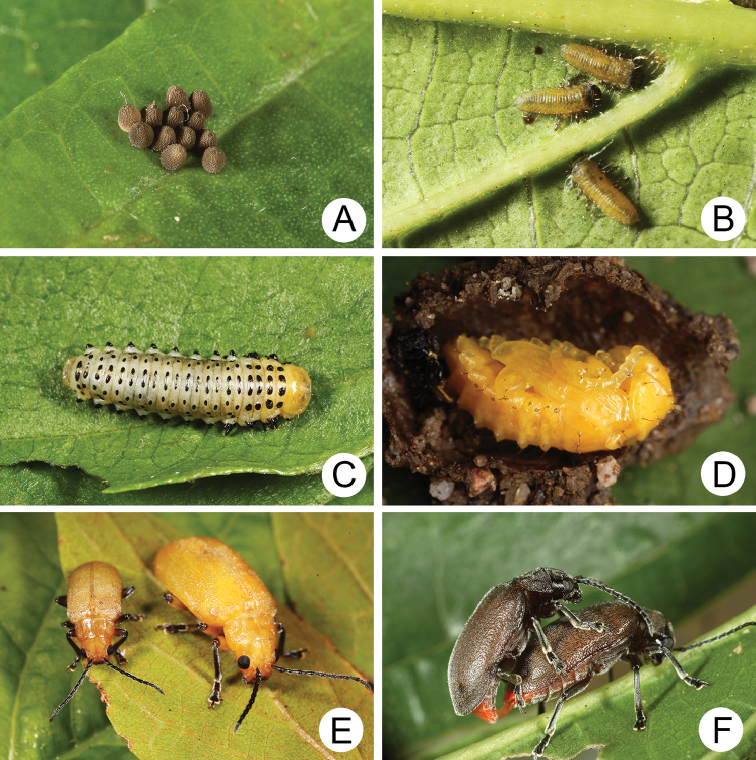
Field photographs of *Pyrrhalta takizawai* Kimoto on host plant **A** egg mass **B** First-instar larvae **C** Third-instar larva **D** pupa **E** young adults **F** older adults.

Figs 49D–F , 51 , 52

Pyrrhalta takizawai Kimoto, 1996: 32; Kimoto and Takizawa 1997: 300 (key), 374; Beenen 2010: 453 (catalogue); Lee and Cheng 2010: 124 (redescription); Xue and Yang 2010: 130 (catalogue); Yang et al. 2015: 121 (catalogue).

##### Types.

***Holotype*** ♀ (SEHU, by original designation): “Nanshanchi (南山溪) / Nantou, Taiwan / 7,12.VII.1983 / H. Takizawa [p, w] // Pyrrhalta [h] / Det. H. Takizawa [p, w] / Pyrrhalta / takizawai / Kimoto, n. sp. [h] / Det. S. Kimoto, 19 [p, w] // HOLOTYPE [p, r] // 00000000154 / Sys. Ent / Hokkaido Univ. / Japan [SEHU] [p, w]”. ***Paratype*.** 1♀ (KMNH): “Nanshanchi / Nantou, Taiwan [p] / 25.VIII [h] 1983 / K. Ra [p, w] // Pyrrhalta / takizawai / Kimoto, n. sp. [h] / Det. S. Kimoto, 19 [p, w] // PARATYPE [p, b] // PHOTO [p, r]”.

##### Other material.

Taiwan. Hsinchu: 1♂ (TARI), Feifengshan (飛鳳山), 5.III.2009, leg. S.-F. Yu; 1♀ (TARI), Kuanhsi (關西), 21.VI.2009, leg. W.-T. Liu; 4♂, 8♀ (TARI), same locality, 24.VII.2010, leg. H. Lee; 2♂, 1♀ (TARI), Peitelaman (北德拉曼), 26.VI.2008, leg. H. Lee; 1♂ (TARI), Shihlu trail (石鹿古道), 23.VIII.2014, leg. Y.-L. Lin; 1♀ (TARI), Talu trail (大鹿林道), 26.VIII.2012, leg. Y.-L. Lin; 1♂ (TARI), Wufeng (五峰), 17.III.2009, leg. S.-F. Yu; 1♂ (TARI), Tahunshan (大混山), 1.III.2009, leg. M.-H. Tsou; Ilan: 2 ♂ (JBCB, NMPC), 20 km N of Ilan city, 2.VI.2008, leg. F. & L. Kantner; Pingtung: 1♂ (TARI), Lilungshan (里龍山), 9.IV.2013, leg. J.-C. Chen; 1♂ (TARI), same locality, 24.III.2014, leg. Y.-T. Chung; 1♂ (TARI), same but with “23.III.2016”; 1♂ (TARI), Neiwen (內文), 12.IV.2013, leg. B.-X. Guo; 1♂ (TARI), Shouka (壽卡), 26.IV.2013, leg. Y.-T. Chung; 1♀ (TARI), same but with “13.VI.2013”; 1♀ (TARI), Shuangliu (雙流), 12.IV.2008, leg. Y.-T. Chung; 10♂, 7♀ (TARI), same but with “25.IV.2018”; 1♀ (TARI), Tahanshan (大漢山), 18.VII.2007, leg. S.-F. Yu; 1♀ (TARI), same locality, 6.VIII.2016, leg. Y.-T. Chung; 1♂ (TARI), Tungyuan (東源), 19.II.2007, leg. S.-F. Yu; Taipei: 1♂, 1♀ (TARI), Chiachiuliao (加九寮), 26.IV.2008, leg. H. Lee; 1♂ (TARI), Fushan (福山), 17.VI.2008, leg. S.-F. Yu; 1♀ (TARI), Pinglin (坪林), 17.VII.2010, leg. Y.-L. Lin; 4♂, 2♀ (TARI), Taipei Zoo, 6.VII.2006, leg. Y.-C. Yu; 1♀ (TARI), same but with “20.X.2006”; 3♀ (TARI), same locality, 10.II.2007, leg. S.-F. Yu; 2♂, 2♀ (TARI), same but with “24.V.2007”; 1♂ (TARI), same but with “27.VI.2007”; 2♂, 2♀ (TARI), same but with “19.I.2008”; 1♀ (TARI), same locality, 24.V.2007, leg. M.-H. Tsao; 2♂♂, 1♀ (TARI), same locality, 10.VII.2007, leg. C.-F. Lee; 4♂ (TARI), Takouhsi (大溝溪), 29.IV.2020, leg. L. Huang; 1♀ (TARI), Yuanshan (鳶山), 22.VIII.2014, leg. S.-F. Yu; Taoyuan: 2♂, 4♀ (TARI), Hsuanyuan (萱源), 21–23.IV.2008, leg. S.-F. Yu; 1♀ (TARI), Lalashan (拉拉山), 4.V.2010, leg. S.-F. Yu; 1♂, 1♀ (TARI), Yongfu (永福), 16.IV.2011, leg. M.-H. Tsou; 7♂, 4♀ (TARI), same but with “4.VI.2011”; 1♂, 1♀ (TARI), same but with “14.III.2015”.

##### Redescription.

Length 10.4–12.3 mm, width 4.3–5.4 mm. Body dark brown or blackish brown (Fig. 49D–F); antennae, tibiae, tarsi, and apices of femora black; teneral individuals with body yellowish brown. Eyes extremely small, interocular space 2.85–3.42 × diameter of eye. Antennae filiform in males (Fig. 51A; length ratios of antennomeres I–XI 1.0: 0.5: 0.6: 0.7: 0.6: 0.6: 0.6: 0.5: 0.5: 0.5: 0.6, length to width ratios of antennomeres I–XI 2.9: 1.8: 2.0: 2.3: 2.0: 2.0: 2.1: 2.1: 2.0: 1.8: 2.6; similar in females (Fig. 51B), length ratios of antennomeres I–XI 1.0: 0.5: 0.6: 0.7: 0.6: 0.6: 0.6: 0.6: 0.6: 0.5: 0.7, length to width ratios of antennomeres I–XI 2.9: 1.7: 1.8: 2.3: 2.0: 2.1: 2.2: 2.2: 2.0: 2.0: 3.1. Pronotum and elytra convex. Pronotum 1.9–2.0 × broader than long, disc smooth; and with extremely dense, coarse and fine punctures, and short pubescence; with median longitudinal and lateral depressions; lateral margins moderately rounded, widest at apical 1/3, apical and basal margins slightly concave; anterior and posterior setiferous punctures slightly erect. Elytra broad, parallel-sided, 1.6–1.7 × longer than wide; disc smooth, with dense, coarse punctures; and extremely dense short pubescence, some pubescence located between coarse punctures. Apical spur of tibia of middle leg absent, tarsomeres I of front and middle legs enlarged in males. Aedeagus (Fig. 51C, D) broad in dorsal view, 4.0 × longer than wide, broadest at apical 1/6, strongly narrowed from apical 1/6 to apex, apex narrowly rounded, gradually narrowed from apical 1/6 to base; symmetric; ostium covered by a membrane; strongly curved from apical 1/6 to base in lateral view, apex narrowly acute; no endophallic sclerites. Gonocoxae (Fig. 51E) connected at base, irregularly margined, with six to eight long setae near apex of each gonocoxa. Ventrite VIII (Fig. 51I) well sclerotized, apical margin moderately concave at middle, fringed with dense long and short setae; spiculum extremely short. Receptacle of spermatheca (Fig. 51F) very swollen; pump extremely long and strongly curved; sclerotized proximal spermathecal duct wide and short. Apical margin of abdominal ventrite V with deep notch at middle in males (Fig. 51H); shallow notch in females (Fig. 51G).

##### Remarks.

Adults of *P. takizawai* Kimoto are similar to those of *P. igai* Kimoto and *P. meihuai* sp. nov. in having large, brown bodies but differ by the sparse pubescence on the pronotum (vs. dense pubescence on pronotum in *P. igai* and *P. meihuai* sp. nov.), sparse, coarse punctures on elytra (vs. dense, coarse punctures on elytra in *P. meihuai* sp. nov.; sparse, fine punctures on elytra in *P. igai*). The form of the aedeagus, gonocoxae, and female abdominal ventrite VIII are also diagnostic.

##### Host plant.

Larvae and adults feed on leaves of *Celtis sinensis* Pers. (Cannabaceae).

##### Biology.

Adults were collected from Taipei City Zoo, January 19, 2008 and transferred to the laboratory for rearing. Females began to deposit an average of 10–20 eggs in single egg mass (Fig. 52A) during middle March. Larvae hatched in 7 days. The larvae (Fig. 52B) fed on leaves and the larval duration was 14 days. mature larvae (Fig. 52C) burrowed into soil and built underground chambers for pupation. Duration of the pupal stage (Fig. 52D) was 28–30 days. Newly emerged adults (Fig. 52E) were yellowish brown and appeared during spring and were active (Fig. 52F). They became darker during summer and autumn and were inactive during winter.

##### Distribution.

The species is widespread at lowlands (0–1,500 m) in Taiwan.

### Key to Taiwanese species of *Xanthogaleruca* and *Pyrrhalta* (*X. aenescens* excluded)

**Table:** 

1	Antenna extremely slender, antennomeres III–V more than 3.0 × longer than wide	**2**
–	Antenna long or stout, antennomeres III–V less than 3.0 × longer than wide	**8**
2	Antennae and legs black; elytra yellow with black margins	**3**
–	Antennae and legs yellowish brown; part of elytra green, or yellowish brown elytra with brown longitudinal stripes	**4**
3	Elytra with dense, fine punctures, and black stripes along suture; tibiae entirely black (Fig. 45A–C)	***P. kobayashii* Kimoto**
–	Elytra with sparse, coarse punctures, black stripes and spots variable; tibiae yellowish brown with lateral margin black (Fig. 38D–I)	***P. shirozui* Kimoto**
4	Elytra at least partly green, without brown longitudinal stripes	**5**
–	Elytra yellowish brown, with brown longitudinal stripes (Figs 5G–I, 7C, D)	***P. tahsiangi* sp. nov.**
5	Elytra with longitudinal ridges, apically brown	**6**
–	Elytra smooth, lacking longitudinal ridges, apices green	**7**
6	Elytra with coarse punctures and sparse pubescence (Fig. 5A–C)	***P. gressitti* Kimoto**
–	Elytra with fine punctures and dense pubescence (Fig. 11D–F)	***P. viridipennis* Kimoto**
7	Elytra green with yellow lateral margin (Fig. 11A–C)	***P. taiwana* Kimoto**
–	Elytra green with wide brown band along suture (Figs 5D–F, 9F)	***P. houjayi* sp. nov.**
8	Pronotum with three large black spots, one at middle, two laterally	**9**
–	Pronotum without black spots	**13**
9	Body flattened; elytra metallic green (Fig. 1D–F)	***X. yuae* sp. nov.**
–	Body convex; elytra brown, reddish brown, or dark brown	**10**
10	Body reddish brown; elytra with five pairs of black spots, one pair near base, two pairs near middle, two pairs at apical 1/3 (Fig. 23)	**11**
–	Body brown or dark brown, elytra with black stripes at humeral calli	**12**
11	Antennomere III elongate, 4.5 × longer than wide, apically expanded in males (Fig. 24A); tarsomere I of middle leg modified in males (Fig. 24K)	***P. maculata* Gressitt & Kimoto**
–	Antennomere III short, 2.7–3.4 × longer than wide, antennomere IV with small tubercle in males (Fig. 25A); tarsomere I of middle leg not modified in males	***P. tsoui* Bezděk & Lee**
12	Small species, 4.3–5.0 mm in length; elytra relatively narrow, 1.7 × longer than wide, disc with dense coarse punctures, with one additional pair of longitudinal dark stripes between humeral calli and suture (Fig. 38A–C)	***P. jungchani* sp. nov.**
–	Large species, 6.0–7.9mm in length; elytra relative broad, 1.5 × longer than wide, disc with sparse fine puncture, lacking longitudinal dark stripes between humeral calli (Fig. 45G–I)	***P. lineatipes* (Takei)**
13	Smaller species, less than 6.5 mm in length	**14**
–	Larger species, more than 6.5 mm in length	**21**
14	Elytra with ridges	**15**
–	Elytra smooth, lacking ridges	**17**
15	Elytra with regular dark spots between ridges (Fig. 34A–C)	***P. ishiharai* Kimoto**
–	Elytra unicolorous, without dark spots	**16**
16	Smaller species, 3.3–3.7 mm in length; elytra relatively broad, 1.5 × longer than wide (Fig. 34D–F)	***P. wulaiensis* sp. nov.**
–	Larger species, 4.5–4.6 mm in length; elytra relatively narrow, 1.7–1.8 × longer than wide (Fig. 49A–C)	***P. ohbayashii* Kimoto**
17	Elytra relatively narrow, 1.7–1.8 × longer than wide, entirely yellowish brown or black, disc with sparse, fine punctures (Fig. 41)	***P. lui* sp. nov.**
–	Elytra relatively broad, 1.4–1.6 × longer than wide, entirely reddish brown, or yellowish brown with black margin and suture, disc with dense, coarse punctures	**18**
18	Body entirely reddish brown	**19**
–	Elytra yellow or partly yellow	**20**
19	Legs reddish brown (Fig. 30A–C); tibia of middle leg with apical spine (Fig. 28G), tarsomere I modified (Fig. 28M), and sides of ventrite V strongly shortened in males (Fig. 28K)	***P. formosanensis* sp. nov.**
–	Legs black (Fig. 30D–F); tibia of middle leg lacking apical spine, tarsomere I not modified, and sides of ventrite V normal in males	***P. semifulva* (Jacoby)**
20	Elytra entirely yellowish brown (Fig. 15D–F)	***P. meifena* Kimoto**
–	Elytra yellowish brown with black margin and suture, sometimes black band along suture enlarged or with additional transverse black bands (Fig. 31)	***P. discalis* Gressit & Kimoto**
21	Larger species, 10.4–12.3 mm; elytra with sparse coarse punctures (Fig. 49D–F)	***P. takizawai* Kimoto**
–	Smaller species, 7.3–8.7mm; elytra with dense fine punctures	**22**
22	Body black (Fig. 15A–C)	***P. alishanensis* sp. nov.**
–	Body brown	**23**
23	Discs of pronotum and elytra with reticulate microsculpture (Fig. 18A–C)	***P. igai* Kimoto**
–	Discs of pronotum and elytra smooth, lacking reticulate microsculpture (Fig. 18D–F)	***P. meihuai* sp. nov.**

## Discussion

The taxonomic relationship of *Pyrrhalta*, *Tricholochmaea*, and *Xanthogaleruca* has been controversial for many decades. Laboissière (1934) proposed *Xanthogaleruca* as a subgenus of *Galerucella* characterized by antennomere III equal or slightly shorter than IV, with the following antennomeres twice as long as wide, and tibiae ridged. Bechyné (1961) listed *Xanthogaleruca luteola* from Afghanistan and implicitly treated *Xanthogaleruca* as a genus. Silfverberg (1974) examined the aedeagi of *X. luteola* (Müller, 1766) and *X. subcoerulescens* (Weise, 1884) and described a comb-shaped internal sclerite. Subsequent authors were not consistent with either genus or subgenus concepts of *Xanthogaleruca*, and it has been treated as a distinct genus (e.g., Beenen 2008, 2010; Beenen and Talpur 2019; Nie et al. 2017; Warchałowski 2003, 2010; Riley et al. 2002, 2003), a subgenus of *Pyrrhalta* (e.g., Wilcox 1965), or a synonym of *Pyrrhalta* (e.g., Wilcox 1971; Yang et al. 2015; Nie et al. 2012; Kimoto and Takizawa 1997).

*Tricholochmaea* was described by Laboissière (1932) as a subgenus of *Lochmaea* Weise, 1883. However, Gressitt and Kimoto (1963) synonymized *Tricholochmaea* with *Pyrrhalta*. Similar to the situation in *Xanthogaleruca*, the concept of *Tricholochmaea* has not been treated consistently by subsequent authors. It has been regarded as a genus (e.g., Beenen 2010; Warchałowski 2010; Riley et al. 2002, 2003), a subgenus of *Pyrrhalta* (e.g., Wilcox 1965, 1971), or a synonym of *Pyrrhalta* (e.g., Xue and Yang 2010). The characters used to distinguish *Tricholochmaea* and *Pyrrhalta* are superficial, including tibiae with ridges the entire length or with traces only (Warchałowski 2010), or the presence of an asymmetrical aedeagus (Wilcox 1965).

The *Pyrrhalta* genus complex badly requires comprehensive revision based on molecular data of species from the whole distributional area. The revision of Taiwanese species supports inclusion of *Tricholochmaea* as part of the *Pyrrhalta semifulva* species group within *Pyrrhalta*. This species group also comprises maculate species traditionally classified in *Pyrrhalta* (cf. Bezděk and Lee 2019). However, we treat *Xanthogaleruca* as a distinct genus based on the characteristic comb-like sclerite of the aedeagus and apparent phylogenetic distance from *Pyrrhalta* as proposed by Nie et al. (2017a), but the genus-level arrangement presented in this paper should be treated as tentative.

Some characters presumed to be important for generic diagnosis are not supported by the present study. The apical spur of the middle leg in males appears across whole genus and species groups, or in some species within different groups, including *Xanthogaleruca*; *Pyrrhalta gressitti*, *P. tahsiangi* sp. nov., and *P. viridipennis* within the *P. gressitti* species group; *P. maculata*, *P. tsoui*, *P. formosanensis* sp. nov., and *P. ishiharai* within the *P. semifulva* species group; and *P. jungchani* sp. nov. and *P. shirozui* within the *P. shirozui* species group. Some of these species have tarsomere I of the middle leg modified, including *P. tahsiangi* sp. nov. within the *P. gressitti* species group; *P. maculata*, *P. formosanensis* sp. nov., and *P. ishiharai* within the *P. semifulva* species group; *P. jungchani* sp. nov. and *P. shirozui* within the *P. shirozui* species group. Groups based on other morphological characters such as the ratio of length vs. width for each antennomere and elytra; sizes and genitalic characters in both sexes are more diagnostic for sorting species within the genus. Such groupings are corroborated by phylogenetic relationships of host plants and shared feeding behaviors. Members of *Xanthogalerucae* feed on leaves of *Ulmus* species or *Zelkova serrata* (Ulmaceae), those of the *Pyrrhalta gressitti* species group feed on leaves of leaves of *Rhododendron* species or *Vaccinium randaiense* (Ericaceae), those of the *P. meifena* species group feed on leaves of *Acer* species (Sapindaceae), those of the *P. semifulva* species group feed on flowers of *Meliosma rhoifolia* (Sabiaceae) or species of Rosaceae, and those of the *P. shirozui* species group feed on leaves of *Viburnum* species (Adoxaceae). This suggests that information about host plants and feeding behaviors may be helpful in grouping species of *Pyrrhalta*.

Species richness of *Pyrrhalta* may be underestimated based on the following reasons. Most *Pyrrhalta* species are monophagous; for example, four species of the *P. meifena* species group feed on *Acer* species (Sapindaceae), of which six species are found in Taiwan (Li and Lo 1993). This suggests 0.66 species of *Pyrrhalta* per species of *Acer*; similarly, five species of the *P. gressitti* species group feed on leaves of one or two species of the genus *Rhododendron* (Li et al. 1998), and 13 species of *Rhododendron* are recorded from Taiwan, suggesting only 0.38 species of *Pyrrhalta* per species of *Rhododendron*.

## Supplementary Material

XML Treatment for Xanthogaleruca

XML Treatment for Xanthogaleruca aenescens

XML Treatment for Xanthogaleruca yuae

XML Treatment for Pyrrhalta

XML Treatment for Pyrrhalta gressitti

XML Treatment for Pyrrhalta gressitti

XML Treatment for Pyrrhalta houjayi

XML Treatment for Pyrrhalta tahsiangi

XML Treatment for Pyrrhalta taiwana

XML Treatment for Pyrrhalta viridipennis

XML Treatment for Pyrrhalta meifena

XML Treatment for Pyrrhalta alishanensis

XML Treatment for Pyrrhalta igai

XML Treatment for Pyrrhalta meifena

XML Treatment for Pyrrhalta meihuai

XML Treatment for Pyrrhalta semifulva

XML Treatment for Pyrrhalta maculata

XML Treatment for Pyrrhalta tsoui

XML Treatment for Pyrrhalta formosanensis

XML Treatment for Pyrrhalta semifulva

XML Treatment for Pyrrhalta discalis

XML Treatment for Pyrrhalta ishiharai

XML Treatment for Pyrrhalta wulaiensis

XML Treatment for Pyrrhalta shirozui

XML Treatment for Pyrrhalta jungchani

XML Treatment for Pyrrhalta lui

XML Treatment for Pyrrhalta shirozui

XML Treatment for Pyrrhalta kobayashii

XML Treatment for Pyrrhalta lineatipes

XML Treatment for Pyrrhalta ohbayashii

XML Treatment for Pyrrhalta takizawai
